# vibe‐qc: An Open‐Source, General‐Purpose Electronic‐Structure Code for Molecules and Solids Developed With Generative AI

**DOI:** 10.1002/jcc.70525

**Published:** 2026-10-27

**Authors:** Michael F. Peintinger

**Affiliations:** ^1^ Mulliken Center for Theoretical Chemistry University of Bonn Bonn Germany

**Keywords:** electronic‐structure software, generative‐AI‐assisted development, periodic systems, quantum chemistry, vibe‐coding

## Abstract

We present vibe‐qc, a general‐purpose open‐source electronic‐structure code for molecules and solids, developed almost entirely with agentic AI under expert human direction. The code is released under the Mozilla Public License 2.0 (MPLv2). The implementation comprises a C++17 numerical core and Python calculation drivers. Molecular methods include Hartree–Fock, Kohn–Sham DFT, Møller–Plesset theory and double hybrids, coupled cluster, active‐space methods, and excited states. Dispersion corrections, continuum solvation, and effective core potentials are also available. Periodic calculations use Gaussian‐basis HF and DFT with Brillouin‐zone sampling. Molecular and periodic method coverage differs; the feature matrices specify analytic and numerical derivatives and distinguish validated and experimental implementations. The validation set is a key contribution of this work. It combines independent program comparisons, published benchmarks, and internal consistency tests. Molecular mean‐field energies agree at the nanohartree scale, and conventional CCSD(T) energies agree within $3.3\times 10^{-7}E_{h}$ for the tested systems. Local‐correlation and periodic limitations are reported with the comparisons. Numerical results, calculation metadata, and analysis scripts are supplied as machine‐readable Supporting Information. The independently maintained vibe‐view viewer, vibe‐queue scheduler, and qvf toolkit support visualization and job management; trexio provides wavefunction interchange.

## Graphical abstract

vibe‐qc is an open‐source, general‐purpose electronic‐structure code for molecules and solids, developed almost entirely with agentic AI under expert human direction. Molecular and periodic calculations use a common interface. Independent reference calculations form a reusable validation set. The vibe‐view viewer, vibe‐queue scheduler, and qvf toolkit are available as separate projects.

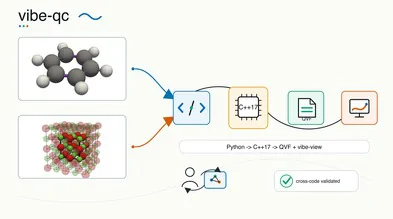

## 1 Introduction

Implementing and maintaining electronic‐structure methods requires substantial work beyond their mathematical formulation. Scientific software often outlives the students and postdoctoral researchers who wrote it, making continuity and reuse important concerns [1, 2]. Community libraries have reduced this burden. Integral engines, exchange–correlation libraries, crystal‐symmetry tools, and simulation frameworks let new programs build on established numerical implementations rather than recreate them.

Generative‐AI programming assistants allow a domain scientist to direct implementation, testing, debugging, and maintenance of scientific software. Independent reference calculations are required to assess the resulting code.

vibe‐qc began as an experiment in this development model. A first Hartree–Fock prototype was checked against an independent program after one day [3]; the initial broad implementation developed over roughly ten weeks, with validation and revision continuing afterward. Development used conversational programming, often called “vibe‐coding”, under the direction of one domain scientist. Its public development record [3, 4, 5, 6] and the workflow described in Section 6.1 document the division of work. Here, agentic AI refers to assistants that change code and execute tests under human direction. The author selected the physical models, assessed the results, and approved releases.

The resulting program treats molecules and solids through a common interface and includes numerical kernels, calculation drivers, file interchange, analysis, and documentation. Molecular methods span mean‐field theory, perturbation theory, coupled cluster, active‐space methods, and excited states. The correlated wavefunction and linear‐response methods discussed here are molecular; periodic calculations are assessed at the HF and KS‐DFT levels. Molecular ECPs and population analysis are included. The code relies on the reusable libraries described in Section 2.

We compare against orca [7], openmolcas [8, 9], pyscf [10], crystal 23 [11], and gpaw [12] because their formulations provide appropriate references for the selected methods. Other established packages include psi4 [13], turbomole [14], q‐chem [15], and gaussian [16]. Molecular and periodic coverage in one package is already an established aim, notably in pyscf and crystal.

We describe the development and release of this molecular and periodic quantum‐chemistry code, its methods, and its architecture. A key contribution is the validation set, which distinguishes agreement between programs under matched settings, accuracy against published benchmarks, and internal consistency. Completed comparisons that exceed the stated tolerance are included. The results and recorded settings are supplied in machine‐readable form.

The companion viewer vibe‐view, scheduler vibe‐queue, and qvf toolkit are maintained independently. This article serves as the common release reference for these projects. Sections 2 and 3 describe the methods and interfaces, Sections 4 and 5 report the comparisons and representative calculations, and Section 6 discusses the development method and limitations. Section 7 presents the conclusions. Detailed method references, support matrices, convergence data, and implementation notes are in the Supporting Information. The software identities are given in Section 8.

## 2 Theory and Methods

vibe‐qc combines a C++17 numerical core with Python calculation drivers. Gaussian integrals and their derivatives, exchange–correlation functionals, crystal symmetry, ECP integrals, and Fourier transforms use libint [17], libxc [18], spglib [19, 20], libecpint [21], and FFTW [22]. The molecular and periodic methods use these libraries for the corresponding numerical operations. Table 1 summarizes the scope of the reported evidence. Supporting Information S10 gives the detailed method‐support tables and original references. The support tables identify the systems, settings, and derivatives covered by the comparisons.

**TABLE 1:** Scope of the evidence reported in this paper. Detailed spin, derivative, and integral‐approximation support is given in Supporting Information S10.

Area	Evidence and limitation
Molecular mean field	HF and KS‐DFT comparisons against independent programs; DFT agreement depends on quadrature and integral approximations.
Molecular correlation	Small‐molecule MP2, CCSD(T), and active‐space comparisons; no general production‐size memory claim.
Local correlation	Pilot calculations quantify truncation errors relative to canonical results; matching a named PNO setting does not establish cross‐code parity.
Periodic methods	Selected GDF and BIPOLE comparisons and convergence ladders; these do not establish accuracy across all cells and meshes.
Derivatives	Analytic and numerical derivatives are identified separately. Energy validation does not validate a gradient or Hessian.

### 2.1 Molecular Methods and Derivative Scope

Molecular calculations cover restricted, unrestricted, and restricted open‐shell HF references and KS‐DFT (Supporting Information S10.1). The functional interface supports LDA, GGA, kinetic‐energy‐density‐dependent meta‐GGAs, global hybrids, and range‐separated hybrids with the VV10 correlation kernel. It does not support Laplacian‐dependent meta‐GGAs. For open shells, UKS is the default. The conventional restricted open‐shell ground‐state KS ansatz cannot represent negative spin densities [23]. Supporting Information S10.1 explains its optional role and distinguishes modern low‐spin excited‐state ROKS formulations. Initial guesses and convergence algorithms are also defined and cited there.

The B3LYP convention is explicit: b3lyp and b3lyp5 select the VWN5 form, corresponding to libxc HYB_GGA_XC_B3LYP5; b3lypg selects the VWN‐RPA form, corresponding to libxc HYB_GGA_XC_B3LYP, the B3LYP functional originally published by Stephens et al. [24]. The evaluated identifier is printed in the output. Comparisons must match this choice [25, 26]. Supporting Information S10.2 gives the functional definitions and conventions.

Analytic gradients cover HF and the supported semilocal and global‐hybrid KS families, with reference‐specific restrictions in Supporting Information S10.3. Range‐separated hybrid and VV10 gradients are unavailable; optimization uses numerical derivatives of the total energy. Native optimization supplies analytic gradients for a restricted single‐state CASSCF subset. The source audit shows that public MP2, CCSD(T), and DLPNO optimization and frequency requests follow the mean‐field reference surface; these paths do not provide derivatives of the correlated energy. The water frequencies reported here use finite differences of the analytic RHF gradient through the calculation interface. The derivative support audit distinguishes these numerical Hessians from routines labelled analytic whose response terms still require verification. No general analytic HF/KS Hessian coverage is claimed; in particular, the ASE UKS path uses finite differences. No analytic correlated, meta‐GGA, or range‐separated Hessian is claimed.

The molecular validation includes MP2 and spin‐scaled variants, B2PLYP (Supporting Information S10.4), conventional and density‐fitted CCSD(T) (Supporting Information S10.5), CASCI, CASSCF, NEVPT2 (Supporting Information S10.6), and linear‐response excitation energies (Supporting Information S10.7). D3(BJ) and D4 corrections (Supporting Information S10.8), continuum solvation, and molecular ECPs (Supporting Information S10.9) extend the tested scope. Their equations, reference implementations, and restrictions are documented with the individual Supporting Information comparisons. The local‐correlation hierarchy remains a pilot implementation; its measured truncation error is reported separately from canonical cross‐code agreement.

### 2.2 Periodic Methods

Periodic HF and KS‐DFT use Bloch‐summed Gaussian orbitals and explicit Brillouin‐zone sampling [27, 28] (Supporting Information S10.10). The molecular correlated‐method coverage does not extend to crystals. The periodic implementation offers Gaussian density fitting (GDF), a direct lattice‐sum route termed BIPOLE, and an experimental Gaussian‐and‐plane‐waves (GPW) route with a GAPW augmentation. Only the documented GDF and BIPOLE comparisons support the periodic claims made here.

GDF approximates Coulomb and exchange terms with an auxiliary Gaussian basis [29, 30] (Supporting Information S10.11). Its accuracy depends separately on the real‐space lattice cutoff and the reciprocal‐space fitting tail. BIPOLE evaluates the short‐range interaction directly and the long‐range interaction under an Ewald split [31, 32] (Supporting Information S10.12). Its production force path uses finite differences of the converged total energy at zero temperature, or Mermin free energy under Fermi smearing [33]; analytic forces remain a research preview. Matching the exchange‐divergence convention is essential when comparing periodic absolute energies. Supporting Information S4 reports the measured residuals and convergence limits. Supporting Information S10.13 describes the convergence aids and density mixing.

Periodic XC quadrature includes cross‐cell density contributions and a partition over lattice images, following the Gaussian‐basis treatment of Towler, Zupan, and Causà [34] (Supporting Information S10.14). Symmetry and mesh reduction use spglib (Supporting Information S10.15). Neither those algorithms nor a converged SCF alone establishes cross‐code energy agreement. Variable‐cell optimization, periodic ECP calculations through the public drivers, and the experimental GPW/GAPW routes are outside the validated scope.

## 3 Implementation and Companion Tools

### 3.1 Numerical Core and Calculation Interface

Figure 1 shows the numerical core, calculation drivers, and companion projects. The C++17 layer evaluates the integral‐driven Fock builds, lattice sums, derivative kernels, and XC grids. It uses eigen [35] for dense linear algebra and OpenMP for shared‐memory parallelism. Python handles calculation setup, file IO, properties, and workflow control across a pybind11 interface [36]. The high‐level entry points are run_job for molecules and run_periodic_job for crystals; Supporting Information S7 gives input examples. Geometry optimization uses the ase calculator interface, with a native optimizer available for installations without ase.

**FIGURE 1:**
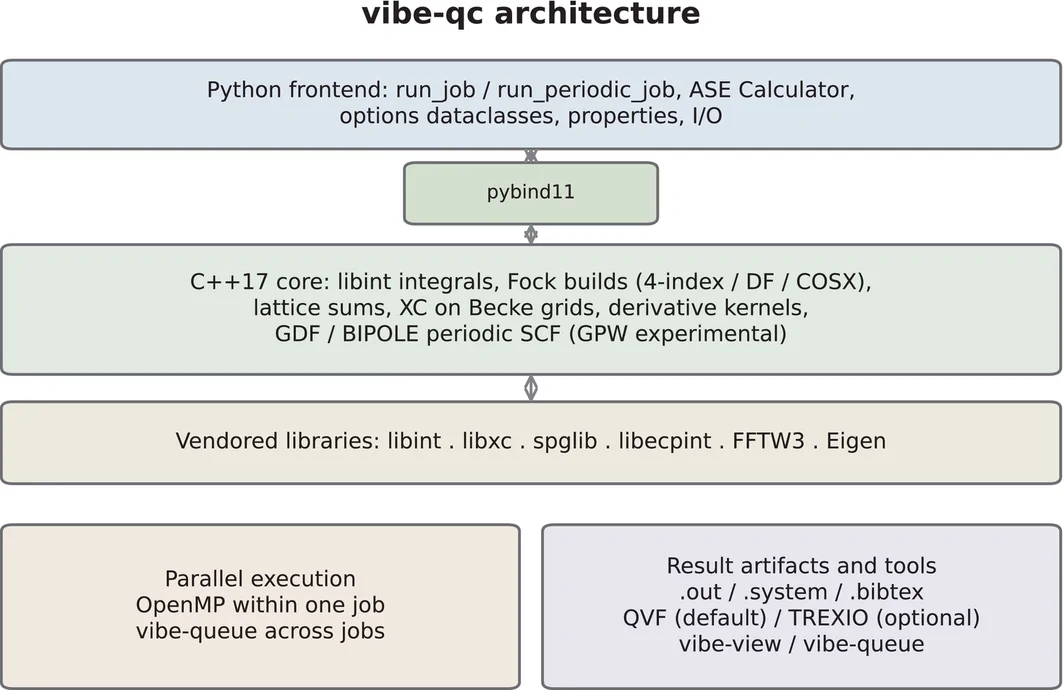
Python calculation drivers over a C++17 numerical core, with established numerical libraries beneath and independently maintained companion projects for visualization, scheduling, and the QVF specification.

Basis sets can be imported through the JSON interchange format of the Basis Set Exchange [37], as well as Gaussian‐94, orca, and crystal text formats. The crystal format is useful for the pob families [38, 39]; it does not define a distinct ECP capability. Installation and dependency versions are given in Section 8.

### 3.2 Interchange and the Release Toolset

vibe‐qc performs the calculations, vibe‐view displays the results, and vibe‐queue (command vq) schedules jobs. The three are independently maintained projects. The qvf specification and reference toolkit are maintained separately under Apache 2.0.

Optional wavefunction interchange uses trexio [40]. The writer exports molecular and periodic SCF wavefunctions, scalar ECPs, and molecular CI expansions; a general data API handles additional fields. Supporting Information S5 distinguishes automatic export, native reconstruction, and vibe‐view rendering, and lists the corresponding tests and limitations. The molecular interoperability checks passed for the development builds specified there. An actual water TREXIO file was opened and rendered in vibe‐view; its files, build identities, and screenshot are supplied in Supporting Information archive 3, vibe‐qc‐jcc‐v3‐si‐03‐trexio‐example.zip. These checks are distinct from the publicly tagged release snapshots.

qvf stores visualization and analysis results, calculation metadata, and citations in one archive. vibe‐view reads these archives to display structures, densities, spectra, and periodic properties. Conventional exports also support established tools, including Avogadro [41], IQmol [42], PyMOL [43], and VESTA [44]. The terminal viewer moltui by Szenes [45] inspired the terminal visualization feature in vibe‐view. Compatible exports also support the use of moltui with vibe‐qc.

vibe‐queue coordinates the calculation campaign. Its scheduler architecture is summarized in Supporting Information S6. Calculation inputs and outputs remain usable without this scheduler, and no dependency on it is required to use vibe‐qc. Implementation details are given in the Supporting Information and the project documentation.

## 4 Validation

We assess the code by comparison with independent programs, comparison with published benchmark data, and internal consistency tests. Program comparisons use matched physical and numerical settings. Published benchmarks assess method error, not implementation correctness; these results are retained in Supporting Information S3. The results of these three types of tests are reported separately.

The molecular comparisons use orca 6.1.1 and openmolcas; periodic Gaussian calculations use pyscf and crystal 23. Reference programs execute out of process. Inputs must match geometry, basis and ECP, charge and spin, frozen‐core treatment, integral approximations, quadrature, convergence settings, and, for crystals, cell and sampling conventions. The method name alone is insufficient to establish equivalent settings. Supporting Information S1–S4 documents these settings and the individual comparisons.

### 4.1 Molecular Comparisons

Table 2 summarizes representative results. Molecular HF energies agree at the nanohartree scale. Conventional CCSD(T) agrees within $3.3\times 10^{-7}E_{h}$ over eight system–basis combinations, whereas the DF‐CCSD comparison on H_2_O/cc‐pVDZ differs from its conventional reference by $1.4\times 10^{-4}E_{h}$. The distinction identifies the integral approximation as part of the comparison, rather than assigning one accuracy to all coupled‐cluster routes. CASSCF comparisons require matched active spaces and openmolcas orbital solutions. Internal FCI and finite‐difference checks are labelled separately in the Supporting Information.

**TABLE 2:** Representative molecular cross‐code comparisons. Values are absolute deviations unless stated otherwise. Supporting Information S3 gives the individual methods, settings, and recorded release identities.

Method or quantity	System/basis; reference	Agreement
RHF, UHF, ROHF	H_2_O, OH, NH_2_/def2‐TZVP; orca	$1.2\times 10^{-9}E_{h}$ maximum
KS‐DFT ladder	H_2_O/def2‐TZVP; orca	$1.5\times 10^{-4}E_{h}$ maximum
CCSD(T), conventional	H_2_O, CO/eight basis cells; orca	$\leq 3.3\times 10^{-7}E_{h}$
CASSCF	N_2_/cc‐pVDZ, (6e,6o); openmolcas	$\leq 3.0\times 10^{-10}E_{h}$
TDDFT, TDA	CH_2_O/def2‐SVP, five roots; orca	$4.1\times 10^{-3}\mathrm{eV}$
Molecular ECP	I_2_/dhf‐TZVP; orca	$1.32\times 10^{-8}E_{h}$
S22 interaction energies	22 complexes/PBE‐D3(BJ)/def2‐SVP; orca	$0.016\;\text{kcal}\;{\mathrm{mol}}^{-1}$ MAD

Molecular DFT requires particular care. A matched‐reference rerun with density fitting disabled in orca removed about 92% of the median deviation in one input family. The remaining difference depends on XC quadrature. Grid ladders on two cases and grid‐free HF controls distinguish these contributions but do not establish an explanation for every functional and system. Those historical inputs also selected a legacy grid and therefore do not measure the shipped default. Supporting Information S8 reports the failure groups and the limits of these diagnoses. Disagreements remain visible rather than being removed by a retrospective change of tolerance.

### 4.2 Periodic Comparisons and Convergence

Periodic agreement depends on both the numerical route and the reference convention. For MgO/STO‐3G at Γ, a GDF cutoff ladder reaches $2.7\times 10^{-5}E_{h}$ against pyscf with the tested automatic tail setting and $2.2\times 10^{-7}E_{h}$ with both cutoffs converged. These are archived ladder measurements, rather than scored database comparisons. On LiH the real‐space lattice cutoff is the limiting parameter. The two examples do not establish a universal default for all‐electron crystals.

The BIPOLE route has an independent diamond/PBE0 check at Γ: orbital energies agree with pyscf within $0.4\;mE_{h}$, the total energy within $1.9\;mE_{h}$, and the gap within 9 meV. The database also contains 1 scored periodic cross‐code total‐energy pass: LiF/pob‐TZVP‐rev2 RHF on a 2×2×2 mesh, with the Ewald exchange convention matched between BIPOLE and pyscf, agreeing to $0.25\;mE_{h}$. Supporting Information S8 quantifies the reference's fitting uncertainty for this comparison.

Other periodic comparisons retain unresolved cutoff, geometry, or convention differences. In particular, the MgO BIPOLE comparison with crystal 23 uses slightly different lattice constants and cannot be scored as a same‐geometry parity result. Supporting Information S4 presents these completed diagnostic calculations with their limitations. GPW/GAPW measurements are not used to support validated release capabilities.

### 4.3 Regression Tests and the Validation Database

The regression suite tests numerical kernels, interfaces, and failure handling; the independent‐program runner tests selected scientific calculations. Test collection counts are not counts of successful cross‐code calculations. Supporting Information S2 states the snapshot and scope of the test inventory, while Supporting Information S9 describes the validation workflow.

The machine‐readable database contains 5839 comparisons with 3861 registered case records and 8433 associated program runs. It reports 3329 passes, 1188 missed tolerances, 1073 unscored completed comparisons with stated scientific limitations, and 249 other paired numerical records. Incomplete attempts, superseded comparisons, and results with a disproven molecular identity are omitted from scientific totals; their original records remain recoverable. Completed comparisons with valid identities that miss a tolerance are retained.

Passing rows have different meanings: 1570 compare against an independent program, 1263 belong to benchmark‐accuracy sets, 451 compare vibe‐qc against itself, and 45 use stored consensus references. Only the first category directly measures agreement with an independent program. Most molecular comparisons use orca; fewer periodic comparisons are available. Supporting Information S8 gives the family and tolerance tables; Section 8 describes the results supplement.

## 5 Representative Calculations

The following calculations illustrate geometry optimization, vibrational analysis, molecular correlation, and effective core potentials. Numerical settings and input examples are given in the Supporting Information. The results supplement identifies the corresponding calculations.

### 5.1 Molecular Optimization and Vibrational Frequencies

The water dimer is optimized at PBE0‐D3(BJ)/def2‐TZVP through the ase bridge (script M07), exercising analytic KS gradients and the post‐SCF dispersion contribution to the forces under a force‐only convergence criterion (maximum force $0.02\;\mathrm{eV}\;{Å}^{-1}$). The optimized O⋯O separation is 3.0873 Å for the symmetric double‐donor arrangement preserved from the starting geometry. The case validates the gradient and dispersion forces on a fixed hydrogen‐bond topology and is not a conformational search for the global‐minimum dimer structure. Harmonic frequencies and IR intensities for the water monomer (script M14) are obtained from the run_job frequency driver, which differentiates the analytic RHF gradient by central finite differences (Section 2.1). The symmetric and antisymmetric stretches and the bend are reproduced to within $0.11\;{\mathrm{cm}}^{-1}$ of the analytic orca Hessian. The associated qvf archives retain the results for inspection in vibe‐view.

### 5.2 Benzene

Benzene is optimized at PBE0‐D3(BJ)/def2‐TZVP (script M20). The optimized C–C bond length is 1.3874 Å. Repeating the optimization across the release builds and optimizer generations archived with the paper gives values that spread over $1.8\times 10^{-4}\;Å$, which is why the value is quoted to four decimals.

### 5.3 A Colocated Timing Control

Timing is reported only from colocated controls, in which both programs run on the same node with the same allocated CPU count and memory. For hydrogen peroxide at DLPNO‐CCSD(T)/cc‐pVTZ with one CPU and 6144 MiB each, vibe‐qc required 699.88 s wall time against 156.67 s for orca 6.1.1, a factor of 4.47, with peak resident memory of 1015.5 MiB and 2007.2 MiB respectively. vibe‐qc is slower in this comparison; the implementation requires further optimization. A second control on canonical CCSD(T) is given in the Supporting Information.

### 5.4 Heavy Elements Through Effective Core Potentials

I_2_ at RHF/dhf‐TZVP (script M09) exercises the shipped dhf small‐core ECP through libecpint on a heavy main‐group element. The total energy differs from the matched orca calculation by $1.3\times 10^{-8}\;E_{h}$, placing the molecular ECP path close to the mean‐field parity floor of the all‐electron routes.

## 6 Discussion

### 6.1 The Development Methodology

The author selected the physical models, architecture, and validation criteria, reviewed the results, and approved releases. AI assistants translated equations into C++ kernels, implemented the Python calculation interface and tests, and drafted documentation. Anthropic Claude was used for the main development, DeepSeek for routine code generation, and OpenAI tools for debugging and refactoring. Implementations were often reviewed or re‐derived in a separate session. Supporting Information S9 gives the model versions and development chronology.

Small reference calculations were used during implementation. Agreement with an independent program under matched settings provided a regression target for subsequent optimization and refactoring. Tests also checked symmetry, conservation laws, convergence, and calculation metadata. The test inputs and physical assumptions required review as well as the calculated values.

For example, an apparent MP2 discrepancy arose from different frozen‐core conventions. Molecular DFT comparisons required separate assessment of density fitting and quadrature. Periodic comparisons required consistent exchange‐divergence corrections and convergence of the lattice and fitting cutoffs. A previously reported XC comparison could not be reproduced and was withdrawn.

The initial implementation was developed over roughly ten weeks, followed by further validation and revision. This observation describes the progress of one project; it is not a controlled estimate of productivity. The work depended on established numerical libraries and human scientific supervision. The validation set is a key contribution: it provides reference comparisons for assessing the code and for subsequent development.

### 6.2 Design Decisions

Separate repositories allow vibe‐view, vibe‐queue, and the qvf toolkit to be used with other calculation programs. trexio provides wavefunction interchange, while qvf stores visualization and analysis results. Established workflow systems include QCEngine and QCArchive [46, 47].

The reported calculations use libint for integral evaluation, including derivatives and periodic integrals. Although libcint [48] provides an alternative, no short‐term replacement of libint is planned. In the longer term, we aim to replace the current integral mechanism with an approach based on Neese's SHARK integral generation and digestion system [49]. This development is outside the scope of the present work. The reusable trust‐region solver libopentrustregion [50] and the Molpro iterative‐solver library [51] are possible alternatives to the current solvers. They have not been integrated into vibe‐qc.

Mulliken and Löwdin populations remain available for comparison with familiar outputs, but lack a basis‐set limit. IAO partial charges [52] are stored in QVF for the supported closed‐shell all‐electron calculations listed in Supporting Information S10.16; they are not included in the text population reports. Geometry optimization is driven through ase, with a native optimizer also available. The additional potential‐energy‐surface drivers are native code rather than ASE workflows. Their scope is described in the software documentation; they are not used as validated demonstrations in this paper.

### 6.3 Scope of the Claims

Molecular HF and ECP comparisons establish tight agreement on the reported cases. DFT and correlated comparisons require explicit grids, auxiliary bases, frozen‐core conventions, and numerical thresholds. The MP2, CCSD(T), and active‐space evidence is concentrated on small systems. It does not establish production‐size memory coverage or accuracy of every spin and derivative combination. Local correlation remains a pilot capability with measured truncation errors.

Periodic tests include GDF cutoff‐convergence calculations and BIPOLE comparisons, including the multi‐*k* LiF case. Appropriate cutoffs still need to be established for each crystal. GPW/GAPW, variable‐cell optimization, public periodic ECP execution, and correlated periodic methods are outside this paper's validated scope. The same applies to unsupported analytic derivatives and the excited‐state gradient whose energy surface has not been verified.

Shared‐memory OpenMP is the validated parallel execution mode. Independent calculations can be distributed through the queue. Production MPI execution, GPU compute kernels, and binary wheels are not included in the reported tests. Implementation updates are recorded in the issue tracker and software documentation.

### 6.4 Reproducibility and Provenance

The reported calculations used releases in the 0.15–0.17 series. Each result retains the recorded build identity. The supplementary CSV and JSON tables preserve stable case, run, and comparison identifiers, values, units, comparison outcomes, and available method and runtime metadata. Their scripts reproduce the reported aggregates. Empty historical metadata fields mean unrecorded information, not a default setting. The supplement supports reanalysis of the results; it is not a complete archive for rerunning every original calculation.

Reproduction of a calculation requires the source code, inputs, numerical settings, and software dependencies. The AI conversations used during development are not required to execute the resulting program.

## 7 Conclusions

We have presented vibe‐qc, a general‐purpose open‐source quantum‐chemistry code for molecules and solids developed almost entirely with agentic AI under expert human direction. The program provides molecular mean‐field, correlated, active‐space, and excited‐state methods, and periodic HF and DFT through a common Python interface and C++17 core. The implementation uses established numerical libraries.

The validation set is a key contribution of this work. Independent program comparisons, published benchmarks, and regression tests assess the implemented methods. The support matrices distinguish molecular and periodic coverage, analytic and numerical derivatives, and experimental implementations. Measured disagreements and limitations are included with the results.

The calculation code, vibe‐view viewer, vibe‐queue scheduler, and qvf toolkit are available as separate open‐source projects. trexio supports wavefunction interchange. Machine‐readable results, calculation metadata, and analysis scripts are supplied with the article for reuse and independent analysis.

## 8 Software and Data Availability

### 8.1 Software

vibe‐qc, vibe‐view, and vibe‐queue use the Mozilla Public License, version 2.0; the qvf specification and toolkit use Apache 2.0. Development remains on the project's self‐hosted server, where the development and validation tooling is integrated. Source access there was provided on request through deploy keys for server protection. Public GitHub mirrors provide direct anonymous access. An automatic mirroring mechanism updates the corresponding repository whenever a new version of any of the four codebases is released. Their public repositories are https://github.com/vibe‐qc/vibe‐qc, https://github.com/vibe‐qc/vibe‐view, https://github.com/vibe‐qc/vibe‐queue, and https://github.com/vibe‐qc/qvf. Anonymous source retrieval was verified on 16 September 2026. Documentation is hosted at https://vibe‐qc.com. The paper's source baseline is vibe‐qc v0.17.1. Its public GitHub snapshot resolves to 164542af38178da1c62717524a13ccc824075302; the original source identity is b4f6035e40d5f15e239c6c45845aba5e33f316f0. The public repository contains sanitized source snapshots rather than identical original Git history. Historical calculations retain their recorded builds. Supporting Information S1 lists the verified companion tags and distinguishes them from the later builds used for the TREXIO demonstration.

### 8.2 Results

The journal Supporting Information includes the numerical results as UTF‐8 CSV and newline‐delimited JSON, with stable identifiers, units, method and settings metadata, comparison outcomes, a schema, analysis scripts, and SHA‐256 checksums (Supporting Information archive 2, vibe‐qc‐jcc‐v3‐si‐02‐results.zip). Summary tables and benchmark statistics can be reproduced from this package without access to the calculation archive. A separate TREXIO water example contains the actual exported wavefunction, imported QVF files, numerical checks, build identities, and the recorded vibe‐view rendering. Supporting Information S8 explains the results selection and historical metadata limitations. The supplement is a results dataset, not a complete native calculation archive.

### 8.3 Installation

The source installation uses scripts/install.sh to build the native dependencies; the source can be retrieved with git clone https://github.com/vibe‐qc/vibe‐qc.git. There is no binary wheel. Supporting Information S1 gives the dependency versions, hardware, and comparison protocol. OpenMP is the validated parallel execution mode.

## Author Contributions

**Michael F. Peintinger**: conceptualization, methodology, software architecture, validation, formal analysis, and writing. The generative‐AI tools described in Section 6.1 (Anthropic Claude Opus 4.7, Opus 4.8, Opus 5, and Fable 5; DeepSeek Flash, Pro, and v4 Pro; and OpenAI Codex 5.5 and ChatGPT 5.6 Sol) assisted in code generation, test scaffolding, documentation, and manuscript drafting under the author's scientific control. The author reviewed, verified, and edited the AI‐assisted output and takes responsibility for the design, validation, interpretation, and manuscript. No AI tool is listed as an author.

## Conflicts of Interest

The author declares no conflicts of interest.

## Supporting information


**Data S1:** Computational details, test sets, validation and convergence tables, software and data provenance, input examples, and method‐support details.

**Data S2:** Machine‐readable validation results in CSV and JSON formats, with case and run identifiers, recorded calculation settings, comparison outcomes, analysis scripts, schema, and checksums.

**Data S3:** Water RHF/def2‐SVP TREXIO interchange and visualization example, including the exported wavefunction, imported QVF files, numerical checks, software‐build identities, and recorded vibe‐view rendering.

## Data Availability

The machine‐readable results and TREXIO rendering example accompany this article as journal Supporting Information. The dataset includes the reported values, recorded settings, analysis scripts, schema, and checksums; its scope and the software identities are described in Section 8.
