# Supplementary material for: vibe‐qc: An Open‐Source, General‐Purpose Electronic‐Structure Code for Molecules and Solids Developed With Generative AI
Source: J Comput Chem. 2026 Oct 27;47(28):e70525. doi: 10.1002/jcc.70525 (PMC13712201; doi:10.1002/jcc.70525)
Supplement: Supplementary file 1 [file JCC-47-0-s003.pdf]

Supporting Information

VIBE-QC: an open-source, general-purpose electronic-structure code for molecules and solids developed with generative AI

Michael F. Peintinger
Mulliken Center for Theoretical Chemistry, University of Bonn, Bonn, Germany
mpei@uni-bonn.de

This document collects the computational details, the test set, the per-method validation tables, the periodic convergence data, the per-job provenance and citation artifacts, the job scheduler as used for this work, representative input listings, and the summary of the validation database. Numerical comparison records are supplied separately as CSV and JSON with a schema, as described in Section S8 and Section 8 of the main text. The results supplement supports numerical reanalysis and includes available method and runtime metadata. It is not a complete native input/output archive. Historical values retain their recorded identities. Script identifiers refer to the original calculation set.

Contents

S1 Computational details **2**

S2 Test set **4**
S2.1 Large-molecule coverage 4
S2.2 Additional release-matrix coverage 6
S2.3 Automated regression-suite structure 6

S3 Per-method benchmarks **7**
S3.1 Molecular method benchmarks 8
S3.2 Two-code consensus reference energies 10
S3.3 Additional methods against ORCA 6 12
S3.4 Literature-benchmark artifacts 14
S3.5 RIJCOSX SCF and gradient against ORCA, per method 14
S3.6 Published benchmark sets 14

S4 Periodic benchmarks and convergence data **16**
S4.1 Periodic methods against the matched reference 16
S4.2 GDF fitting-cutoff convergence 17
S4.3 First multi-route band-gap comparison 18
S4.4 Internal check: Ewald lattice-sum primitive 18
S4.5 Comparison against published reference values 19

S5 Per-job artifacts **19**
S5.1 Provenance manifest (`.system`) 19
S5.2 Automatic citation manifest (`.references` / `.bibtex`) 20
S5.3 The QVF visualization archive 20

S5.4 TREXIO implementation and validation 20

S6 The VIBE-QUEUE job scheduler **21**

S7 Input examples **23**

S8 The validation database **23**

S9 Implementation details **28**
S9.1 Reproducibility artifacts and automatic citations 28
S9.2 Documentation, examples, and tutorials 28
S9.3 Development toolchain 28
S9.4 Development chronology 30

S10 Method support and methodological details **30**
S10.1 Molecular self-consistent field 33
S10.2 Exchange–correlation functionals 34
S10.3 Nuclear gradients and Hessians 34
S10.4 Møller–Plesset theory and double hybrids 35
S10.5 Coupled cluster: canonical, local, and frozen-natural-orbital 35
S10.6 Multireference and non-mean-field methods 36
S10.7 Excited states and electron propagators 36
S10.8 Dispersion and composite methods 37
S10.9 Implicit solvation and effective core potentials 37
S10.10 Periodic SCF: crystal orbitals and $\boldsymbol{k}$ sampling 37
S10.11 GDF: periodic Gaussian density fitting 38
S10.12 Bipolar-expansion Fock build 38
S10.13 Multi-$\boldsymbol{k}$ convergence aids and density mixing 39
S10.14 Periodic Becke partition for XC grids 39
S10.15 Space-group symmetry 39
S10.16 Population analysis, bonding analysis, and properties 40

S1 Computational details

Software. Unless a row states otherwise, calculations were produced with VIBE-QC releases of the 0.15 to 0.17 series, built from source by the bootstrap script against the vendored native dependencies in Table S1. Because the code is under active development, a version string alone does not identify a build. The results supplement preserves recorded build and runtime metadata. The `.system` manifest format is illustrated in Section S5. Historical metadata gaps are identified as unrecorded fields (Section S8); no release identity is inferred for them. Each comparison uses its recorded input and output settings; the listings in Section S7 provide representative examples.

Reference programs. Cross-code comparisons used ORCA 6.1.1 for molecular methods, OPENMOLCAS for CAS and multireference methods, PYSCF for periodic Gaussian density fitting, CRYSTAL 23 for the

Table S1: Native and build-time dependencies used by the release build. Header-only build dependencies are not reported as calculation runtime values.

Library	Version	License
LIBINT	2.13.1	LGPL-3.0
LIBXC	7.0.0	MPL-2.0
SPGLIB	2.7.0	BSD-3-Clause
LIBECPINT	1.0.7	MIT
FFTW3	3.3.9	GPL-2.0-or-later
EIGEN	5.0.1-compatible	MPL-2.0 (header-only)
`pybind11`	≥2.12	BSD-3-Clause (build-time, header-only)

bipolar-expansion route and the periodic-XC comparisons, and GPAW for the Gaussian-and-plane-waves route. Reference programs were executed as separate processes; VIBE-QC links to numerical libraries rather than to these programs. Parity comparisons require matching basis and auxiliary basis, integration grid, convergence thresholds, frozen-core treatment and k-mesh. Documented mismatches are retained as diagnostic comparisons. The result tables identify the recorded methods, settings, and comparison limitations.

Hardware. The reported calculations were run through `vq` on five workstation-class hosts and two university clusters. The workstations were an Intel Core i9-10885H (8 physical / 16 logical cores, 62 GB), an AMD Ryzen 9 9950X (16 / 32 cores, 64 GB), an AMD Ryzen 9 5950X (16 / 32 cores, 126 GB), and an Intel Core i5-8400 (6 cores, 32 GB), all under Manjaro Linux 7.0.10/7.0.13, together with an Apple M5 Max (18 cores, 128 GB) under macOS 26.5.1. The remainder ran on a PBS/Torque cluster whose nodes span Intel Xeon X5670 (12 cores, 94 GB), Xeon E5-2620 (12 cores, 252 GB), Xeon Silver 4114 (20 cores, 93 GB), and AMD Opteron 6376 (32 / 64 cores, 252 GB) partitions under openSUSE Tumbleweed 6.5.x, and on a Slurm cluster with Intel Xeon Platinum 8468 nodes (96 cores, 1008 GB) under AlmaLinux 9.6. Node-level hostnames are pseudonymized in the archived manifests. The manifests record 1–128 OpenMP threads for the reported calculations. The BLAS/LAPACK backend was OpenBLAS with LAPACKE on Linux and Accelerate on macOS.

Numerical settings and data identity. The case and run tables record available basis, auxiliary-basis, spin, route, and numerical settings. Initial-guess and backend choices matter because AUTO depends on the reference and system. PNO truncation settings must be matched for local-correlation parity. Historical fields that were not recorded remain empty. The SI states settings relevant to its selected comparisons, including the periodic cutoffs in Section S4.

Cross-platform reproducibility. Two rows were repeated on two independent clusters from the same build to separate host effects from method effects. The M01 RHF/def2-TZVP and M02 UHF/def2-TZVP decks were each run on a Slurm host and on a PBS/Torque host. The two VIBE-QC total energies agree to 1.4×10^{-13} E_h for M01 and are bit-identical for M02, and each run separately matches its own ORCA 6.1.1 reference on its own host, by 1.231×10^{-9} E_h and 2.831×10^{-10} E_h. These two calculations test reproducibility across the two clusters; parallel scaling was not measured.

Timing measurements. Timing comparisons were performed separately because the accuracy calculations used different resource allocations. For a timing pair, the VIBE-QC run and its reference run are executed on the same host and node with one allocated CPU per process and identical requested memory and wall-time limits; the inputs and numerical settings remain those of the accepted accuracy comparison. A timing row is reported only when both processes yield elapsed wall time, active CPU time, peak resident-set size, allocated CPU count, and the primary solver iteration count, taken from program output or from scheduler accounting. Reference runs shorter than 10 s are excluded as startup-dominated, and archived outputs that carry internal component timings but no process-level resource measurement are excluded.

Public source identities. Anonymous GitHub retrieval was verified on 16 September 2026. Public tags include VIBE-QC v0.17.1 and v0.17.2, VIBE-VIEW v2.17.0, VIBE-QUEUE v0.26.7, and QVF v0.1.0. Their exact public commit identities and the mapping to original source identities are supplied in `software_identities.json` in the results supplement. These are source-availability checks, not revalidation of all historical calculations. The water rendering uses the unreleased VIBE-QC 0.17.3 candidate and an original VIBE-VIEW v2.17.1 build whose tag was not present in the public repository at this cutoff. The example retains those actual identities and does not claim that the public v2.17.0 tag produced the render.

S2 Test set

The test set comprises molecular (Table S2) and periodic (Table S5) validation scripts, driven entirely through the high-level `run_job` and `run_periodic_job` API. Each script writes the standard artifact bundle (Section S5); API examples are in Section S7; the calculation package retains the executed decks.

S2.1 Large-molecule coverage

Larger molecular calculations were performed with ORCA single-point references. These tests cover calculation metadata, QVF, Molden, population analysis, and cube output for larger atomic-orbital spaces. Table S3 gives the results. The ORCA references were rerun serially after its parallel startup failed on that cluster, so only the RHF rows are used for matched program comparisons.

The four alkane-formula rows (LM01–LM04) are *synthetic straight-chain structures*, not relaxed molecular geometries. They were produced by an internal chain generator that places the hydrogens of each internal methylene from a carbon–carbon-derived offset; the resulting coordinates carry over-long internal C–H bonds (up to 1.18 Å, against a physical 1.09 Å) and short H $\cdots$ H contacts (down to 1.14 Å). We therefore treat these rows strictly as VIBE-QC-vs-ORCA agreement tests on a fixed, identical set of atomic coordinates in a large AO space, and make no physical claim about *n*-octane or *n*-dodecane. LM05, a benzene dimer, is a genuine physical geometry and is unaffected.

The LM01, LM03, and LM04 rows were subsequently repeated on a later release build of the 0.15 series, with matched ORCA 6.1.1 references. All three reproduce the VIBE-QC total energies tabulated above at the precision displayed, and reproduce the tabulated reference differences 2.50×10^{-8}, 3.71×10^{-8}, and 5.48×10^{-3} E_h; the LM03 and LM04 differences agree with their original values to 8.8×10^{-12} and

Table S2: Molecular test set. Each row is one input script.

ID	Script	System / basis	Method(s)
M01	`m01_rhf_h2o.py`	H_2O / def2-TZVP	RHF
M02	`m02_uhf_oh.py`	OH($^2\Pi$) / def2-TZVP	UHF
M03	`m03_dft_functional_ladder.py`	H_2O / def2-TZVP	PBE, PBE0, B3LYP, r^{2}SCAN, ωB97X
M04	`m04_mp2_family.py`	H_2O / cc-pVTZ	MP2, SCS-MP2, SOS-MP2 (RI)
M05	`m05_double_hybrid_b2plyp.py`	H_2O / cc-pVTZ	B2PLYP
M06	`m06_composite_3c.py`	$(H_2O)_2$	hf-3c, r^{2}SCAN-3c
M07	`m07_dispersion_geomopt.py`	$(H_2O)_2$ / def2-TZVP	PBE0-D3(BJ) + opt
M08	`m08_solvation_cpcm_cosmo.py`	CH_3COOH / def2-TZVP	B3LYP + CPCM
M09	`m09_ecp_heavy.py`	I_2 / dhf-TZVP	RHF + ECP
M10	`m10_wavefunction_solvers.py`	H_2/6-31G; N_2/cc-pVDZ	FCI
M11	`m11_cas_multireference.py`	N_2 / cc-pVDZ	CASCI, CASSCF, NEVPT2, CASPT2 (diagnostic)
M12	`m12_coupled_cluster.py`	H_2O / cc-pVDZ	DF-CCSD, DF-CCSD(T) (density-fitted default; the conventional-route rows come from the explicit `density_fit=False` pairs)
M14	`m14_hessian_ir.py`	H_2O / def2-TZVP	RHF Hessian (finite difference of the analytic gradient) + IR
M15	`m15_rohf_roks.py`	NH_2 / def2-TZVP	ROHF, ROKS
M16	`m16_dlpno.py`	n-butane / cc-pVDZ	DLPNO-MP2/CCSD/CCSD(T)
M17	`m17_excited_states.py`	CH_2O / def2-SVP	TDDFT (Casida), TDA/CIS
M18	`m18_ovgf_ip.py`	H_2O / def2-TZVP	GF2 / OVGF (IP; smoke)
M19	`m19_new_functionals.py`	H_2O / def2-SVP, cc-pVDZ, cc-pVTZ†	r^{2}SCAN01, ωB97X-V, ωB97M-V, revDSD-PBEP86-D4
M20	`m20_benzene_application.py`	C_6H_6 / def2-TZVP	PBE0-D3(BJ) optimization

† M19 runs each functional in the basis of its reported row: r^{2}SCAN01 in def2-SVP, the two VV10-completed range-separated hybrids in cc-pVDZ, and the double hybrid in cc-pVTZ.

Each script reproduces the calculation this paper reports for its identifier. Four of them (M09, M16, M19, P01) were corrected during revision, when a sweep against the archived outputs found that they ran a different system, basis or method from the row they illustrate.

$2.1 \times 10^{-7}\,E_\mathrm{h}$. The LM04 comparison is grid-convention-limited rather than SCF-limited, because its ORCA deck carries no `DEFGRID` keyword and the row therefore compares the two programs' default integration grids. The LM02 and LM05 entries are from the original build.

Reproducibility caveat: initial-guess dependence of LM01. That repeat also established that the LM01 system supports at least three distinct closed-shell RHF/STO-3G stationary points. Varying only the initial guess on a single release binary gives $-306.679\,393\,523\,1\,E_\mathrm{h}$ (`SAP`, and `AUTO`, which selects it), $-306.638\,682\,864\,5\,E_\mathrm{h}$ (`PATOM`, `SAD`, `HCORE`, `MINAO`), and $-306.494\,698\,185\,0\,E_\mathrm{h}$ (`HUECKEL`). All three converge to distinct SCF solutions with positive orbital-energy gaps. The value tabulated above is the lowest of the three, the `SAP/AUTO` solution, which is also the one the matched ORCA reference reproduces. The initial guess must therefore be specified to reproduce this result. We attribute the near-degeneracy to the strained synthetic geometry rather than to any physical property of an alkane: the over-long internal C–H bonds and 1.14 Å H$\cdots$H contacts noted above give a compressed, near-degenerate manifold of low-lying closed-shell solutions, so reproducing the tabulated LM01 number requires the `SAP` guess specifically. We make no claim that any one guess is preferable in general, nor that the list above is exhaustive.

Table S3: Fetched large-molecule coverage. Energies are total VIBE-QC energies from the archived `.out` files. LM01–LM04 are synthetic straight-chain structures with a known geometry defect (see text) and are used only as fixed-coordinate VIBE-QC-vs-ORCA agreement tests, not as physical alkane benchmarks.

ID	System	Method / basis	Status	E_{tot} / E_h
LM01	synthetic C_8H_{18} chain	RHF / STO-3G	converged, QVF	−306.6793935231
LM02	synthetic C_8H_{18} chain	RKS-PBE / def2-SVP	converged, QVF	−313.1624959631
LM03	synthetic $C_{12}H_{26}$ chain	RHF / STO-3G	converged, QVF	−459.1244786257
LM04	synthetic $C_{12}H_{26}$ chain	RKS-PBE / STO-3G	converged, QVF	−462.5765005417
LM05	benzene dimer	RKS-PBE0-D3(BJ) / def2-SVP	converged, QVF	−463.6017562931

The `.system` manifests record the exact VIBE-QC build and branch. ORCA 6.1.1 serial fallback references give RHF total-energy differences of 2.50×10^{-8} and 3.71×10^{-8} E_h for LM01 and LM03. The DFT rows remain coverage/convention checks with differences 6.44×10^{-4}, 5.48×10^{-3}, and 2.64×10^{-2} E_h for LM02, LM04, and LM05.

S2.2 Additional release-matrix coverage

Additional method–system combinations were tested for successful execution and output generation. Table S4 lists these calculations from an earlier build in the series. Independent reference calculations are not available for these entries.

Table S4: Additional release-matrix coverage. All values are total energies taken from the archived `.out` files.

Case	System	Method	Basis	E / E_h
`A_rhf_h2o`	H_2O	RHF	STO-3G	−74.9644538631
`A_pbe_h2o`	H_2O	RKS-PBE	STO-3G	−75.2342885566
`A_b3lyp_h2o`	H_2O	RKS-B3LYP	STO-3G	−75.2826476567
`A_pbe0_h2o`	H_2O	RKS-PBE0	STO-3G	−75.2519152837
`A_r2scan_h2o`	H_2O	RKS-r^{2}SCAN	STO-3G	−75.2937248441
`A_wb97x_h2o`	H_2O	RKS-ωB97X	STO-3G	−75.3129220111
`A_rhf_nh3`	NH_3	RHF	STO-3G	−55.4550218289
`A_pbe_nh3`	NH_3	RKS-PBE	STO-3G	−55.7105508231

Table S5: Periodic inputs associated with the reported completed calculations.

ID	Script	System / basis	Route	k-mesh
P01	`p01_mgo_gamma_gdf.py`	MgO, fcc primitive / STO-3G	GDF, RHF	Γ
P02	`p02_lih_multik_gdf.py`	LiH / STO-3G	GDF, KRHF	(2, 2, 2)
P03	`p03_mgo_multik_bipole.py`	MgO / STO-3G	BIPOLE, KRHF	(2, 2, 2)
P06	`p06_periodic_dispersion.py`	fcc Ne / def2-SVP	GDF + D3(BJ)	(4, 4, 4)

S2.3 Automated regression-suite structure

The automated test infrastructure has two distinct layers. The first is the repo-wide `pytest` suite, which is broad engineering and scientific regression coverage. It verifies numerical kernels, API behavior, physics conventions, data provenance, failure gates, and output artifacts. The second is the standalone `examples/regression` scientific parity runner, a curated set of end-to-end calculations with retained inputs and outputs. Only a subset of the `pytest` suite is direct cross-code parity, so the counts in Table S6 should not be read as thousands of independent reference calculations.

Table S7 gives an approximate first-match classification of the `v0.15.132` collection by test-module name, generated by the classifier script distributed with the paper (`scripts/classify_tests_v0151`

Table S6: Regression-suite and parity-runner counts at the `v0.15.132` snapshot (collection only; no tests executed).

Item	Count
`pytest` test files present	733
`pytest` files with collected tests	726
Collected `pytest` test items	15576
Active `examples/regression` cases	44
Active molecular parity cases	40
Active periodic parity cases	4
Full default parity-run program rows, when ORCA and PySCF are available	128

`32.py`). It is intended to show the breadth of the regression surface, not to replace the method-by-method validation tables in Section S3.

Table S7: Approximate first-match classification of collected `pytest` items by test-module name at the `v0.15.132` snapshot. Tests of research code outside the scope of this paper are not listed.

Area	Tests
Periodic solids: $\boldsymbol{k}$ meshes, Ewald, GDF/GPW/BIPOLE, slabs, bands	4450
Molecular SCF, DFT, XC, grids, convergence controls	1822
Citations, provenance, documentation, release hygiene	2746
Post-HF, coupled cluster, multireference, CAS/MRPT/FCI	1770
Basis sets, ECPs, integrals, DF/RI, numerical kernels	648
Output files, QVF, cubes, visualization, I/O formats	972
Molecular gradients, Hessians, optimization, properties	439
ASE, NEB, MD, MLIP, workflow integration	253
Dispersion and corrections	383
Symmetry and structural utilities	188
Other focused regression tests	425

The full suite includes tests or runners that mention external implementations at the file level, including PySCF (117 files), ORCA (40), CP2K (12), CRYSTAL (77), GPAW (6), Psi4 (5), DFTD3 (7), DFTD4 (9), and ASE (391). These mentions include true parity checks, parser tests, optional-interface tests, fixtures, and documentation/provenance tests.

The 44 active standalone parity cases cover atomic and diatomic smoke systems (H_2, Ne, HF), small closed-shell molecules (CH_4, NH_3, H_2O, H_2CO), RHF and RKS-LDA/PBE/BLYP/B3LYP, MP2, density-fitted RHF and RKS-PBE examples, benzene, open-shell O_2 and O_3 stress cases, and a noncovalent S22 subset. The active periodic cases are Ne fcc, LiH, NaCl, and MgO rocksalts. At this snapshot the active periodic parity cases all use STO-3G/RKS-LDA/$1 \times 1 \times 1$ settings and are used to detect implementation errors. Some periodic comparisons exceed the stated tolerances, as reported in the result tables.

S3 Per-method benchmarks

This section gives benchmarks for methods with completed measurements, each against a single reference program matched to the formalism: ORCA 6 for molecular methods wherever ORCA implements the feature; OPENMOLCAS for the complete-active-space and multireference methods; PySCF for the periodic Gaussian-density-fitting route; and CRYSTAL 23 for the periodic bipolar-expansion route. Table S8 records the reference-program versions and settings. All VIBE-QC numbers come from the archived test set; ref-

erence programs are run out-of-process. The comparisons use method-specific tolerances. Mean-field rows are SCF-limited and agree at the nanohartree scale: RHF/UHF/ROHF to 1.2×10^{-9} E_h and ECP energies to 1.4×10^{-8} E_h against ORCA. The reported active-space energies agree with OPENMOLCAS for the matched cases. The retained CASSCF rerun reproduces its registered energy, a case-specific reproducibility result. The full-configuration-interaction solver is checked for cross-release reproducibility, spanning a development build and the tagged release, rather than against an independent reference program. Correlated and density-functional rows are limited by density fitting and integration grids rather than by SCF convergence, and agree at the tens-of-microhartree to sub-millihartree scale when those approximations define the comparison: MP2 reaches 2.5×10^{-5} E_h, SOS-MP2 6.5×10^{-5} E_h, the single H_2O DF-CCSD benchmark is 1.4×10^{-4} E_h, and the KS-DFT ladder is 1.5×10^{-4} E_h. The route matrix separates this from conventional all-electron CCSD(T), which agrees with ORCA to 3.3×10^{-7} E_h or better over H_2O and CO basis-set checks. Periodic rows are reported in Section S4: the point-charge Madelung foundation is exact to about 1×10^{-11}, the density-fitting route reaches 2.7×10^{-5} E_h against PYSCF at the Γ point with the tested automatic-tail setting and 2.2×10^{-7} E_h with both lattice cutoffs converged, and the multi-$\boldsymbol{k}$ rows remain held under the two truncation mechanisms measured there.

Table S8: Reference programs used as independent references, with the version and the key settings matched to the VIBE-QC run.

Program	Version	Matched settings
ORCA 6	6.1.1	same basis/aux, `TightSCF`, grid
OPENMOLCAS	26.02 (`pymolcas` py2.30)	same basis, active space, `RASSCF`/`CASPT2` settings
PYSCF	2.13.1	same basis/aux, k-mesh, `exxdiv`
CRYSTAL 23	23	same basis, TOLINTEG, shrinking factor
GPAW	26.7.0	same basis, k-mesh, functional

S3.1 Molecular method benchmarks

Table S9: Molecular methods validated against matched reference programs, with the explicitly labelled DF–conv. rows reporting paired VIBE-QC route contrasts; the CASCI, CASSCF, NEVPT2, and CASPT2 rows are validated against OPENMOLCAS. $|\Delta E|$ is the absolute difference in total energy, or correlation energy where the row notes "corr", in hartree.

Method	System / basis	Quantity	Agreement
RHF	H_2O / def2-TZVP	total	1.2×10^{-9}
UHF	OH / def2-TZVP	total	2.8×10^{-10}
ROHF	NH_2 / def2-TZVP	total	3.4×10^{-10}
ROKS-PBE	NH_2 / def2-TZVP	total	9.1×10^{-5}
RKS-PBE	H_2O / def2-TZVP	total	1.4×10^{-4}
RKS-PBE0	H_2O / def2-TZVP	total	1.3×10^{-4}
RKS-r^{2}SCAN	H_2O / def2-TZVP	total	1.5×10^{-4}
RKS-r^{2}SCAN01	H_2O / def2-SVP	total	3.9×10^{-6}
RKS-ωB97X-V	H_2O / cc-pVDZ	total	7.8×10^{-5}
RKS-ωB97M-V	H_2O / cc-pVDZ	total	8.1×10^{-5}
MP2 (all-e corr)	H_2O / cc-pVTZ	corr	2.5×10^{-5}
SCS-MP2 (all-e corr)	H_2O / cc-pVTZ	corr	5.2×10^{-5}
SOS-MP2 (all-e corr)	H_2O / cc-pVTZ	corr	6.5×10^{-5}

Method	System / basis	Quantity	Agreement
CCSD(T) (all-e conv.)	H_2O, CO / cc-pVXZ, def2-XVP	total	$\leq 3.3 \times 10^{-7}$
DF-CCSD (all-e corr)	H_2O / cc-pVDZ	corr	1.37×10^{-4}
DF-CCSD(T) (all-e)	H_2 / def2-SVP	DF–conv. total	2.0×10^{-5}
FNO-CCSD(T) (matched chemical core, no trunc.)	H_2O / def2-SVP	DF–conv. total	9.4×10^{-6}
DF-UCCSD(T) (matched chemical core)	OH / def2-SVP	DF–conv. total	2.3×10^{-5}
CASCI	N_2 / cc-pVDZ (6e,6o)	total	8.2×10^{-9}
CASSCF	N_2 / cc-pVDZ (6e,6o)	total	$\leq 3.0 \times 10^{-10}$
CASSCF grad.	N_2 / cc-pVDZ	$\lvert\nabla\rvert$	$3.8 \times 10^{-5}\, E_\mathrm{h}\, a_0^{-1}$
NEVPT2	N_2 / cc-pVDZ	total	2.0×10^{-6}
CASPT2	N_2 / cc-pVDZ	total	held: 8.9×10^{-5} residual in the second-order correction
FCI	H_2 / 6-31G	total	1.4×10^{-11}
TDDFT (five lowest roots)	CH_2O / def2-SVP	ω / eV	4.1×10^{-3}
CIS (1^1B_2)	CH_2O / def2-SVP	ω / eV	7.6×10^{-4}
RHF Hessian (FD of analytic gradient)	H_2O / def2-TZVP	freq / cm^{-1}	$0.11\, cm^{-1}$
ECP (dhf-ECP)	I_2 / dhf-TZVP	total	1.32×10^{-8}

The I_2/dhf-TZVP ECP row above is a matched small-core comparison. It must not be conflated with a separate H_2S/LANL2DZ comparison in the database, whose case label requested an ECP and whose VIBE-QC leg ran all-electron: the runner of that release did not attach the LANL2DZ ECP sidecar that the basis label implies, while ORCA applied the sulfur ECP and correlated the 8-valence-electron problem. The resulting $-78.628\, E_\mathrm{h}$ difference is a runner defect of the releases before v0.15.108, at which the automatic sidecar attachment was introduced. The row is kept in the database as a fail and is excluded from numerical validation.

For the M18 propagator row the archived VIBE-QC smoke value is 11.2155 eV. ORCA 6.1.1 does not implement the OVGF or GF2 propagator methods, so no direct cross-code comparison exists for this row. The underlying Hartree–Fock orbital energies are compared against ORCA, and the accuracy of the OVGF first ionization energies against an IP-EOM-CCSD reference on nine molecules (0.60 eV mean, 1.22 eV maximum at def2-TZVP) is reported below as a method-accuracy result.

The method-column label "all-e" denotes correlation of all explicitly represented electrons on both sides, without restoring electrons replaced by an ECP. Frozen-core controls are available for MP2, CC and DLPNO at the numerical baseline; their count and orbital conventions are described in Section S10.4. Defaults depend on the method, interface and release. Each accepted comparison must therefore retain its explicit frozen-orbital count and selection instead of inferring them from a program's current defaults. Historical rows retain their actual inputs.

For the M12 closed-shell and corresponding open-shell coupled-cluster comparisons, the original archived comparison mixed ORCA `NoFrozenCore` references with VIBE-QC's default frozen-core convention. The reported DF row in Table S9 therefore uses the later explicit all-electron density-fitted VIBE-QC rerun, with a residual difference against conventional ORCA of $1.37 \times 10^{-4}\,E_h$ for H_2O DF-CCSD. The corresponding triples routes are now also measured in explicit, same-input density-fitted-versus-conventional pairs. The total-energy differences are $1.99 \times 10^{-5}\,E_h$ for H_2/def2-SVP DF-CCSD(T), $2.32 \times 10^{-5}\,E_h$ for OH/def2-SVP DF-UCCSD(T), and $9.35 \times 10^{-6}\,E_h$ for the no-truncation H_2O/def2-SVP FNO-CCSD(T) route. The H_2 pair is all-electron; the OH and H_2O pairs use the same chemical-core freeze in both calculations. These paired calculations isolate the fitting and route difference within VIBE-QC; they are not presented as independent external-code parity. For comparison, VIBE-QC's canonical CCSD reproduces ORCA's canonical CCSD on H_2O/cc-pVDZ to $2.65 \times 10^{-8}\,E_h$. This indicates that essentially all of the $1.37 \times 10^{-4}\,E_h$ DF-CCSD residual arises from density fitting. Re-execution of fourteen correlated cases on the corrected route reproduces every earlier default-route total energy to within $1.1 \times 10^{-9}\,E_h$, so the correction moved no default-route number. The all-electron conventional CCSD(T) comparisons use matched SCF settings: exact-SCF conventional VIBE-QC CCSD(T) agrees with exact-SCF conventional ORCA to $3.3 \times 10^{-7}\,E_h$ or better over H_2O and CO with cc-pVDZ, cc-pVTZ, def2-SVP, and def2-TZVP bases.

The M17 PBE0-TDA excitation energies are 4.0354 eV, 8.5374 eV, 9.2501 eV, 9.9332 eV and 10.3588 eV. The maximum difference over the five roots against the six-decimal ORCA 6.1.1 absorption-spectrum values is 4.1×10^{-3} eV (root 2, 8.5374 against 8.533 337 eV).

S3.2 Two-code consensus reference energies

Alongside the per-method benchmarks, a two-code consensus reference suite (ASCENT) is maintained with the input library and will be described separately. A consensus value is created only when VIBE-QC and ORCA, executed independently at identical geometry, basis set, and protocol, agree within a per-atom tolerance; when the spread exceeds the tolerance, no value is stored. Table S10 lists the complete citable set at the 2026-08-14 snapshot: 44 canonical RHF total energies (38 distinct system–basis pairs, with protocol-variant contracts collapsed onto their shared value) and one exact FCI energy. The largest cross-code spread in the set is $3.1 \times 10^{-9}\,E_h$. Three contract checks are open at this snapshot and produce no consensus value: the LiH/cc-pVDZ cross-code contract ($1.1 \times 10^{-4}\,E_h$, attributed to a cc-pVDZ basis-revision difference between the reference program's built-in library and the BSE-sourced set; the same comparison passes at $5.1 \times 10^{-11}\,E_h$ with the revision matched explicitly), an MgO-cluster cross-code contract ($1.1 \times 10^{-1}\,E_h$), and a RIJCOSX-versus-canonical approximation contract (H_2O/STO-3G, $7.8 \times 10^{-4}\,E_h$ against a $6 \times 10^{-4}\,E_h$ window). A fourth, the GFN2-xTB hydrogen-atom contract, was open at the submitted snapshot and has since been re-measured on the current build and closed exactly. Two of the three open contracts are canonical RHF, which is the count the main text gives. System identifiers are the input-library identifiers; `n-alkane:2` is ethane. These values carry no external archive identifier yet. As an independent check, the release-line VIBE-QC runs recorded in the validation database reproduce all 44 consensus cells within their windows, with a largest deviation of $1.6 \times 10^{-9}\,E_h$ from the stored values; those comparisons are rows of the deposited database (Section S8).

Table S10: Two-code consensus reference energies: canonical RHF total energies on which VIBE-QC and ORCA agree at identical geometry, basis, and protocol, plus one exact FCI energy. The spread column is the absolute difference between the two codes' energies.

System	Basis	E / E_h	Spread / E_h
`atom-mg`	3-21G	−198.468103018	2.1×10^{-10}
`atom-mg`	6-31G**	−199.595219247	4.6×10^{-10}
`atom-mg`	cc-pVDZ	−199.608297028	3.5×10^{-10}
`atom-mg`	def2-SVP	−199.534810664	2.8×10^{-10}
`atom-mg`	STO-3G	−197.007353900	1.5×10^{-10}
`c2h4`	STO-3G	−77.072178778	9.1×10^{-10}
`ch2o`	STO-3G	−112.353831892	7.0×10^{-12}
`ch4`	3-21G	−39.976830650	1.7×10^{-10}
`ch4`	STO-3G	−39.726809171	1.6×10^{-10}
`co`	3-21G	−112.093296738	4.2×10^{-11}
`co`	STO-3G	−111.224558696	3.9×10^{-10}
`co2`	STO-3G	−185.064695683	1.3×10^{-9}
`h2`	3-21G	−1.122928328	9.0×10^{-12}
`h2`	6-31G**	−1.131277627	2.5×10^{-11}
`h2`	aug-cc-pVDZ	−1.128793349	7.6×10^{-11}
`h2`	cc-pVDZ	−1.128714959	6.5×10^{-11}
`h2`	def2-SVP	−1.128906379	1.8×10^{-11}
`h2` (7 protocol variants)	def2-TZVP	−1.132530993	8.0×10^{-12}
`h2`	STO-3G	−1.116684387	2.2×10^{-10}
`h2o`	3-21G	−75.585408880	1.1×10^{-10}
`h2o`	STO-3G	−74.963023139	5.3×10^{-10}
`h2o-dimer`	STO-3G	−149.934156936	6.4×10^{-10}
`h2o2`	STO-3G	−148.673974044	2.5×10^{-9}
`hf`	3-21G	−99.459751767	1.2×10^{-10}
`hf`	STO-3G	−98.570779986	3.9×10^{-10}
`lih`	3-21G	−7.929586512	7.1×10^{-11}
`lih`	STO-3G	−7.862023860	2.3×10^{-10}
`n-alkane:2`	STO-3G	−78.305264172	3.9×10^{-10}
`n2`	3-21G	−108.300238170	7.1×10^{-10}
`n2`	STO-3G	−107.495975031	1.3×10^{-9}
`nacl-cluster:1`	STO-3G	−614.480650190	2.6×10^{-9}
`nh3`	3-21G	−55.870191524	1.5×10^{-10}
`nh3`	STO-3G	−55.454507814	3.7×10^{-10}
`s22-ammonia-dimer`	STO-3G	−110.786163275	3.1×10^{-9}
`s22-methane-dimer`	STO-3G	−79.441572974	4.9×10^{-10}
`s22-water-dimer`	STO-3G	−149.935375927	5.3×10^{-10}
`water-cluster:1`	STO-3G	−74.962928247	5.8×10^{-10}
`water-cluster:2`	STO-3G	−149.813556355	1.7×10^{-9}
`h2` (FCI)	STO-3G	−1.137270175	exact

S3.3 Additional methods against ORCA 6

These are the ORCA-specific method families beyond the core molecular rows in Table S9. Table S11 reports the glycine/def2-TZVP RIJCOSX set (SCF energy and analytic gradient), the DLPNO pilot hierarchy, the composite "-3c" methods, and continuum solvation component audits.

Table S11: Methods benchmarked or audited against ORCA 6.1.1.

Method	System / basis	Quantity	Agreement
RIJCOSX (RHF/UHF/B3LYP/PBE0)	glycine / def2-TZVP	E_{SCF}	$\leq 2.7 \times 10^{-4}\ E_h$
RIJCOSX analytic gradient	glycine / def2-TZVP	$\lvert\nabla\rvert$	held: $4.3 \times 10^{-3}\ E_h\ a_0^{-1}$ RMS
DLPNO-MP2 (corr)	n-butane / cc-pVDZ	corr	pilot: $3.762 \times 10^{-3}\ E_h$
DLPNO-CCSD (corr)	n-butane / cc-pVDZ	corr	pilot: $-3.813 \times 10^{-3}\ E_h$
DLPNO-CCSD(T) (corr)	n-butane / cc-pVDZ	corr	pilot: $-5.909 \times 10^{-3}\ E_h$
HF-3c	$(H_2O)_2$ / MINIX	total	$1.7 \times 10^{-9}\ E_h$
HF-3c gCP + SRB	$(H_2O)_2$ / MINIX	corr. terms	$4.0 \times 10^{-9}\ E_h$
r^2SCAN-3c	$(H_2O)_2$ / def2-mTZVPP	total	held: $1.3 \times 10^{-3}\ E_h$
r^2SCAN-3c, SCF half	$(H_2O)_2$ / def2-mTZVPP	E_{SCF}	held: $1.2 \times 10^{-3}\ E_h$ RI conv.
r^2SCAN-3c, D4 term	$(H_2O)_2$ / def2-mTZVPP	E_{disp}	defect: $6.0 \times 10^{-5}\ E_h$
r^2SCAN-3c, gCP	$(H_2O)_2$ / def2-mTZVPP	gCP	$2.0 \times 10^{-9}\ E_h$
B2PLYP	H_2O / cc-pVTZ	total	$1.4 \times 10^{-6}\ E_h$
B2PLYP, KS half	H_2O / cc-pVTZ	E_{SCF}	$1.3 \times 10^{-6}\ E_h$
B2PLYP, scaled MP2	H_2O / cc-pVTZ	E_{corr}	$7.7 \times 10^{-8}\ E_h$
revDSD-PBEP86-D4	H_2O / cc-pVTZ	total	pass (1 mE_h conv.): $3.982 \times 10^{-4}\ E_h$
D4 dispersion (PBE0)	$(H_2O)_2$ / def2-TZVP	E_{disp}	$2.0 \times 10^{-9}\ E_h$
PBE0-D3(BJ) optimization	$(H_2O)_2$ / def2-TZVP	E, r_{OO}	$3.1 \times 10^{-4}\ E_h$, 3.5×10^{-3} Å
CPCM (water)	CH_3COOH / def2-TZVP	in-solvent E	held: $-9.3 \times 10^{-4}\ E_h$, CPCM vs CPCM

Exact-algorithm agreement. Where VIBE-QC and ORCA run the same algorithm with the same settings, the total energies agree at the numerical floor. Across the matched RHF, UHF, and ROHF comparisons the largest absolute difference is $1.54 \times 10^{-9}\ E_h$; a five-basis H_2O_2 RHF series from STO-3G through cc-pVTZ agrees within $2.55 \times 10^{-9}\ E_h$. Matched ROKS-PBE comparisons are grid-limited rather than SCF-limited and span 2.5×10^{-5}–$1.3 \times 10^{-4}\ E_h$. The MP2 family spans a wider range, 7.17×10^{-9}–$1.016 \times 10^{-4}\ E_h$, whose upper end is a density-fitting effect: the rows that avoid an auxiliary basis mismatch, O_2 across five bases and LiH/def2-TZVP, stay within $1.40 \times 10^{-7}\ E_h$. The ECP path was validated after a provenance failure, when a reconstructed input silently omitted the ECP declaration; with the ECP applied explicitly the I_2 energy is $-589.344\,400\,824\,577\ E_h$ against $-589.344\,400\,837\,791\ E_h$ from ORCA 6.1.1, a difference of $1.32 \times 10^{-8}\ E_h$.

Coupled cluster. The canonical, non-density-fitted route is the parity claim; the matched density-fitted CCSD comparison carries a residual of 0.137 mE_h. The density-fitted triples routes have now been rerun as explicit density-fitted–conventional pairs on the corrected path. Their values in Table S9 are internal route contrasts, not independent external-code parity. Two earlier comparisons were rejected as settings mismatches rather than implementation differences, at 2.808 mE_h and 35.373 mE_h, because VIBE-QC froze

one orbital where Orca froze none; the corrected all-electron reruns are the values reported in Table S9.

Local correlation. The DLPNO hierarchy is reported as convention-held rather than as parity. Against Orca TightPNO the released vibe-qc defaults differ by $-3.813 \times 10^{-3}\,E_\mathrm{h}$ for DLPNO-CCSD and $-5.909 \times 10^{-3}\,E_\mathrm{h}$ for DLPNO-CCSD(T) on the M16 system, and by 0.43–1.18 mE_h on the smaller library cases. The truncation defaults differ on both sides, so these differences measure the default settings, not the correctness of the local-correlation implementation, and we do not present them as a parity result.

Conventions that must be matched before a comparison is valid. Three classes of row are held for definitional rather than numerical reasons. First, basis-set revision: for LiH the Orca basis *label* selects a different primitive contraction than vibe-qc resolves, giving apparent differences of 1.14×10^{-4}, 1.45×10^{-5}, and $-1.54 \times 10^{-7}\,E_\mathrm{h}$ across cc-pVDZ, cc-pVTZ, and cc-pVQZ; regenerating the Orca basis explicitly from the same primitives brings the same comparisons to within $5.1 \times 10^{-11}\,E_\mathrm{h}$, and those explicit controls are the values we report. Second, integration grid: a thirteen-row LiH series comparing Orca DefGrid3 against the vibe-qc default grid differs by 4.10×10^{-6}–$4.02 \times 10^{-4}\,E_\mathrm{h}$, which is a grid specification difference and is excluded from parity claims. Third, solvation and double hybrids: the M08 CPCM in-solvent total differs from Orca by $-9.28 \times 10^{-4}\,E_\mathrm{h}$ and the original M19 revDSD-PBEP86-D4 total by $2.185 \times 10^{-3}\,E_\mathrm{h}$. The M08 comparison was audited after submission: the Orca deck is a genuine CPCM calculation (not SMD), and the vibe-qc number is the in-solvent total, not the SCF banner energy, which sits 55.5 mE_h lower. Solvated total energies embed a model-dependent reference state and must be compared through matched solvent-response components, so the row remains held at that residual. The original revDSD difference was localized by term-by-term decomposition to the local-correlation composition flavor of the Kohn–Sham half (PZ81 against the VWN5 of the defining paper's reference implementation). The post-fix release rerun uses VWN5 and reduces the total residual to $3.982 \times 10^{-4}\,E_\mathrm{h}$, which passes the declared 1 mE_h convention tolerance. This is not a same-parameter-set parity claim: vibe-qc retains the cited 2019 coefficient set, whereas the explicit Orca keyword selects its 2021 revision. The comparison therefore reports the remaining convention difference rather than interpreting it as an implementation error.

Timing. A colocated control illustrates the timing protocol of Section S1. For LiH CCSD(T)/cc-pVQZ, run sequentially in one allocation with one CPU and 8 GiB each, vibe-qc used 21.71 s wall, 21.03 s active CPU, and 932.47 MiB peak RSS, against 35.30 s, 17.03 s, and 369.16 MiB for Orca 6.1.1: symmetric ratios of 1.63, 1.24, and 2.53, all within the factor-of-ten gate. The pair is not algorithmically matched, and the label must say so: the vibe-qc leg ran the density-fitted CCSD(T) that was the default at that release, while the Orca leg ran conventional CCSD(T), which flatters vibe-qc on wall time. The two total energies differ by $2.6 \times 10^{-5}\,E_\mathrm{h}$, the size of the fitting error. A strategy-matched timing pair on the conventional route is below the 10 s reporting floor for this system and is therefore not reported. Two efficiency defects are open and disclosed. The DLPNO routes run 6–11 times slower than Orca at one core on the same inputs, and the RIJCOSX SCF is similarly slow while remaining accurate, the two newly measured UKS rows differing from Orca by $2.079 \times 10^{-4}\,E_\mathrm{h}$ (B3LYP) and $2.431 \times 10^{-4}\,E_\mathrm{h}$ (PBE0). The static memory pre-flight is conservative by a wide margin, with a median requested-to-peak ratio of 26.3 across the successful runs, which indicates conservative allocation in that sample.

S3.4 Literature-benchmark artifacts

Table S12 reports the water-dimer interaction energy from the S22 set [1] computed with and without the D3(BJ) correction, as a check that the dispersion correction enters the interaction energy with the expected magnitude and sign.

Table S12: Water-dimer PBE/def2-TZVP interaction energies from the S22 calculation set.

Route	E_{dimer} / E_{h}	E_{monomer} / E_{h}	ΔE_{int} / kcal mol^{-1}
PBE, no dispersion	−152.7627101655	−76.3765380594	−6.0455
PBE-D3(BJ)	−152.7653804355	−76.3772423994	−6.8371

S3.5 RIJCOSX SCF and gradient against Orca, per method

Table S13 resolves the RIJCOSX comparison by method. The SCF energies agree to a few times 1×10^{-4} E_{h} throughout, which is the expected size of a COSX grid difference, while the analytic gradient does not yet reach parity and is reported as such.

Table S13: Per-method RIJCOSX parity against Orca 6.1.1, glycine/def2-TZVP. Maximum absolute SCF-energy difference across the current refresh is 2.649×10^{-4} E_{h}. The RHF analytic gradient is measured against an Orca `EnGrad` reference on the same geometry and does not reach parity.

Method	$\lvert\Delta E_{\mathrm{SCF}}\rvert$ / E_{h}	max $\lvert\Delta(\partial E/\partial R)\rvert$ / ($E_{\mathrm{h}}\, a_0{}^{-1}$)
RHF	2.648×10^{-4}	1.04×10^{-2}
UHF	2.649×10^{-4}	held
RKS-B3LYP	2.079×10^{-4}	held
RKS-PBE0	2.431×10^{-4}	held
UKS-B3LYP	2.079×10^{-4}	held
UKS-PBE0	2.431×10^{-4}	held
Maximum	2.649×10^{-4}	held

S3.6 Published benchmark sets

Cross-code parity establishes that we solve the same equations as an established program. It says nothing about whether the resulting chemistry is of the quality the method is known to deliver. For that we compare against published benchmark data, and Table S14 reports three such sets. These are accuracy statements about the method as implemented here, not parity statements about the implementation, and the two must not be read as the same claim. Every scored entry in the stated method and setting subset enters the statistics below. Over 409 vertical excitation energies from QUESTDB [2], spanning 32 molecules and compared against the theoretical best estimates at aug-cc-pVTZ, the mean absolute deviation is 0.24 eV for TDA-B3LYP, 0.26 eV for full TD-B3LYP, 0.21 eV and 0.26 eV for TDA- and TD-PBE0, 0.25 eV and 0.28 eV for TDA- and TD-PBE, and 0.42 eV for CIS. That ordering, and those magnitudes, are what the adiabatic linear-response formalism is documented to give for GGAs, global hybrids, and CIS on valence excitations, so the implementation reproduces the known behavior of the methods it provides. Seventy-seven of the 409 entries deviate by more than 0.5 eV and are included. A further 130 QUESTDB entries are excluded for reasons of principle rather than of numerics: doubly excited states, which the adiabatic approximation cannot describe, and fluorescence entries, which refer to a different geometry.

Table S14: Validation against published benchmark data. These are accuracy measures for the method as implemented, not cross-code parity measures. Reference values are the published ones cited in each row. All scored entries are included, and n counts them.

Set	Quantity	Method	n	MAD
QUESTDB [2]	vertical excitation	TDA-B3LYP	59	0.24 eV
		TD-B3LYP	57	0.26 eV
		TDA-PBE0	61	0.21 eV
		TD-PBE0	62	0.26 eV
		TDA-PBE	59	0.25 eV
		TD-PBE	59	0.28 eV
		CIS	52	0.42 eV
BH9 [3]	barrier, forward	PBE	309	7.19 kcal mol^{-1}
	barrier, reverse	PBE	331	9.05 kcal mol^{-1}
	reaction energy	PBE	311	4.93 kcal mol^{-1}
S22 subset [1, 4]	DLPNO truncation error (TightPNO)	DLPNO-CCSD(T)	5	0.17 kcal mol^{-1}
		DLPNO-MP2	22	0.75 kcal mol^{-1}

The barrier heights require the same care in the opposite direction. Against BH9 [3], PBE gives a mean absolute deviation of 7.19 kcal mol^{-1} on forward barriers, 9.05 kcal mol^{-1} on reverse barriers, and 4.93 kcal mol^{-1} on reaction energies over 951 comparisons, retaining 20 outlier rows whose deviations exceed three times the published reference-method error. Those are large numbers, and they are the expected ones: a semilocal GGA systematically underestimates barrier heights by several kcal mol^{-1}, and BH9 is referenced to a DLPNO-CCSD(T_0)/CBS focal point. These benchmark deviations measure PBE accuracy; they do not alone establish implementation parity. We report only PBE here because that is the functional the present campaign covered. A ladder study across BH9 belongs with the functional-assessment literature rather than with a code release paper.

The local-correlation truncation error is measured directly against the canonical result on the S22 subset used by Liakos *et al.* [4], per method and PNO setting. At the TightPNO setting the DLPNO-CCSD(T) interaction-energy truncation error has a mean absolute value of 0.17 kcal mol^{-1} and a maximum of 0.46 kcal mol^{-1} over the five complexes computed, and the DLPNO-MP2 truncation error has a mean absolute value of 0.75 kcal mol^{-1} and a maximum of 2.76 kcal mol^{-1} over 22 complexes, with the largest errors on dispersion-dominated complexes. NormalPNO and LoosePNO results remain in the database with their recorded outcomes but are outside this TightPNO statistic, and DLPNO-CCSD is excluded from this statistic under a tracked convergence defect. The density-fitting error on the corresponding total energies is below the reporting threshold. This quantifies the truncation error left open by the pilot designation of the DLPNO hierarchy in the molecular support table.

The electron propagator illustrates the distinction between the two kinds of claim directly. The underlying Hartree–Fock orbital energies match Orca to 1×10^{-4} eV, which is implementation parity. The OVGF-corrected first ionization energies, measured against IP-EOM-CCSD on the same nine molecules at def2-TZVP, deviate by 0.60 eV on average with a maximum of 1.22 eV, which is method accuracy against that reference. The same calculation is therefore a tight parity result and a loose accuracy result at once, depending on which question is asked of it.

S4 Periodic benchmarks and convergence data

S4.1 Periodic methods against the matched reference

Table S15 gives the completed periodic comparisons against the corresponding reference programs: PySCF.pbc for the Gaussian-density-fitting (GDF) route and Crystal 23 for the bipolar-expansion route. The Γ-point GDF row has a converged-cutoff comparison. The multi-$\boldsymbol{k}$ rows are held under the two truncation mechanisms discussed below. The bipolar-expansion comparison uses a revised reference, with a remaining difference in the lattice constant.

Table S15: Periodic-method benchmarks, one reference per route.

Route / quantity	System / basis	k-mesh	Reference	Agreement
GDF **Γ** RHF[1]	MgO / STO-3G	Γ	PySCF	archived default $-4.596 \times 10^{-1}\,E_\mathrm{h}$; tested automatic-tail setting $2.7 \times 10^{-5}\,E_\mathrm{h}$; both cutoffs converged $-2.2 \times 10^{-7}\,E_\mathrm{h}$ (see text)
GDF multi-$\boldsymbol{k}$ KRHF	LiH / STO-3G	$(2,2,2)$	PySCF	held: $1.585 \times 10^{-3}\,E_\mathrm{h}$ at the default lattice cutoff; lattice-cutoff-limited (see text)
Periodic XC energy	MgO / STO-3G, LDA	$(2,2,2)$	Crystal 23 SHRINK 2	held: $-1.033\,E_\mathrm{h}$ at the archived release, untailed multi-$\boldsymbol{k}$ fit (see text)
Periodic XC energy	MgO / STO-3G, PBE	$(2,2,2)$	Crystal 23 SHRINK 2	held: $-1.034\,E_\mathrm{h}$, as above
Periodic XC energy	MgO / STO-3G, r^{2}SCAN	$(2,2,2)$	Crystal 23 SHRINK 2	held: $-1.034\,E_\mathrm{h}$, as above
BIPOLE multi-$\boldsymbol{k}$ KRHF	MgO / STO-3G	$(2,2,2)$	Crystal 23 SHRINK 8 (k-converged)	$5.19 \times 10^{-3}\,E_\mathrm{h}$, no tolerance class (cells differ by 0.002 Å; see text)

[1] This row is the two-atom primitive MgO/STO-3G Hartree–Fock calculation of the validation database. It is not the test-set script `p01_mgo_gamma_gdf.py` before its revision, which used an eight-atom conventional cell, RKS-PBE, and def2-SVP. The revised input inventory identifies the primitive-cell RHF calculation.

Cutoff-convergence tests identify two sources of error in the density-fitted calculations. For MgO at **Γ**, the reciprocal-space tail of the Coulomb fit is the main contribution: suppressing the tail reproduces the archived $-4.596 \times 10^{-1}\,E_\mathrm{h}$ hold, the release's tested automatic-tail setting on the Γ-point driver, which sizes the tail automatically, closes the difference against PySCF to $2.7 \times 10^{-5}\,E_\mathrm{h}$, and with both the tail and the real-space lattice cutoff converged the comparison reaches $-2.2 \times 10^{-7}\,E_\mathrm{h}$. The archived value additionally carried about $4 \times 10^{-2}\,E_\mathrm{h}$ from a superseded electrostatic route. The energy is insensitive to the real-space lattice cutoff for this cell (constant to $1 \times 10^{-12}\,E_\mathrm{h}$ from 21 to 30 a_0). For LiH, the main error instead arises from the real-space lattice cutoff; the reciprocal tail has little effect, and the $1.585 \times 10^{-3}\,E_\mathrm{h}$ row converges to below $1.5 \times 10^{-6}\,E_\mathrm{h}$ once that cutoff reaches 24 a_0 on the driver-level ladder. The cutoff

is not yet available through `run_periodic_job`; the driver-level result is therefore reported separately from the original comparison.

On the multi-$\boldsymbol{k}$ MgO cell the tail accounts for a measured 0.505 E_h of the roughly 1.03 E_h offset against CRYSTAL 23 on the Hartree–Fock probe. The remainder is not a difference of reference convention: the sealed CRYSTAL 23 values agree with a same-cell PySCF calculation to 0.71 mE_h on all three functionals, so the remaining difference cannot be attributed to disagreement between the reference programs. At the archived release the tail correction was not applied on the multi-$\boldsymbol{k}$ Kohn–Sham path at all, which is why the three XC rows sit at −1.03 E_h. That defect has since been fixed: with the tail applied on the Kohn–Sham path, the LDA row closes to −3.6 mE_h on the fixed release, in a separate diagnostic measurement. The historical rows remain labelled by their original settings.

The P03 BIPOLE KRHF MgO calculation completed and wrote `.out`, `.system`, population sidecars, density, DOS, bond-order, and QVF artifacts. The total energy is −271.212 957 677 3 E_h. The submitted version of this table compared it against a sealed CRYSTAL 23 Hartree–Fock value obtained with SHRINK 2, giving $E_{\mathrm{vibe-qc}} - E_{\mathrm{CRYSTAL23}} = 6.434\,388 \times 10^{-1}$ E$_h$ and describing the difference as a gauge offset. That reference has since been retired by the maintainers of the sealed table. CRYSTAL 23 applies no finite-size exchange correction, so its SHRINK-2 exchange samples the $q \to 0$ term at the coarse mesh's smallest $|q|$ and over-binds its own SHRINK-8 value by 0.638 E_h, whereas the BIPOLE route applies the probe-charge Ewald correction and its $(2, 2, 2)$ energy is already k-converged. Against the k-converged SHRINK-8 value in the same sealed table (−271.218 143 749 82 E_h, 29 irreducible $\boldsymbol{k}$) the difference is 5.19 mE_h. No tolerance class is assigned to that residual, because the two legs are not at one geometry: the sealed CRYSTAL 23 cell has $a = 4.21$ Å and the VIBE-QC leg $a = 4.212$ Å. The three DFT rows of the same table are unaffected: they carry no exact exchange, and their SHRINK-2 references agree with PySCF on the identical cell to 0.65 mE_h. The property outputs give Mulliken charges of ±0.843566 e, Löwdin charges are ±0.703709 e, the Mg–O Mayer order is 1.645 991, and the ordinary three-dimensional bulk dipole is marked unsupported.

The historical MgO GDF/XC total energies are −271.531 535 514 7 E_h (LDA), −272.799 650 390 4 E_h (PBE), and −272.963 026 737 0 E_h (r^{2}SCAN). They do not establish agreement with CRYSTAL 23: the absolute offsets against the sealed SHRINK-2 references remain about −1.033 E_h, −1.034 E_h, and −1.034 E_h. Cross-build repeats reproduce the P01, P02, and MgO GDF/XC totals at table precision, confirming that those values are stable across builds.

The archived $\boldsymbol{\Gamma}$ GDF baseline for this row is $E = -271.509\,106\,034\,5$ E$_h$, -4.596×10^{-1} E_h from PySCF at the archived release's default; the tested automatic-tail setting and the converged-cutoff values quoted above supersede it.

S4.2 GDF fitting-cutoff convergence

Table S16 reports the multi-$\boldsymbol{k}$ KRHF energy of primitive LiH at a $(2, 2, 2)$ mesh as a function of the GDF fitting cutoff, against the PySCF `KRHF.density_fit` reference. The tight cutoff is stable but remains $1.585\,179 \times 10^{-3}$ E_h above the archived PySCF reference, and does not meet the stated agreement criterion.

Table S16: Primitive LiH (2, 2, 2) KRHF energy versus GDF fitting cutoff, against PySCF.

Cutoff	E / E_h	ΔE vs PySCF/ E_h
recorded default	−7.9203949568	$1.608\,278 \times 10^{-3}$ E_h
tight	−7.9204180555	$1.585\,179 \times 10^{-3}$ E_h

S4.3 First multi-route band-gap comparison

The first held band-gap comparisons across two Fock-build routes are recorded at the 2026-08-14 database state. Table S17 reports sampled-$\boldsymbol{k}$-set gaps for LiF (pob-TZVP-REV2, PBE, (2, 2, 2) mesh) from the bipolar-expansion route against three independent reference programs, together with the diamond density-fitted value. The three references themselves span 0.145 eV on this system, which sets the scale at which a gap comparison can discriminate between implementations at all. No band-gap tolerance is assigned; these are unscored diagnostic comparisons. The bipolar-expansion gap is read from the post-rewrite artifact; the pre-rewrite and post-rewrite values agree to 1.5×10^{-10} E_h in the total energy.

Table S17: Sampled-$\boldsymbol{k}$-set band gaps (eV) from the VIBE-QC Fock-build routes against three independent reference programs, PBE, (2, 2, 2) mesh, pob-TZVP-REV2. Every row is a diagnostic rather than a scored parity result; the bipolar-expansion comparison against PySCF is drawn from cells that differ within a recorded bound.

System	Route	Gap	Reference	Ref. gap	Δ
LiF	BIPOLE	8.752	PySCF	8.718	+0.034
LiF	BIPOLE	8.752	CRYSTAL 23	8.791	−0.039
C (diamond)	GDF	4.532	GPAW	4.559	−0.027

S4.4 Internal check: Ewald lattice-sum primitive

The following is an internal unit check of the reciprocal-space lattice-sum primitive, not a cross-code validation. The Madelung constant of a lattice is an exact geometric sum, so reproducing it confirms only that the Ewald machinery converges to the right mathematical limit; it says nothing about the self-consistent electrostatic convention that the single-point comparisons in Table S15 probe. It is recorded here for completeness because a failure of this check would invalidate everything built on top of it.

Table S18: Internal convergence check of the Ewald lattice-sum primitive against the exact geometric Madelung constants.

Lattice	VIBE-QC	Accepted	Deviation
NaCl (rocksalt)	1.7475645946	1.7475645946	6.34×10^{-12}
CsCl	1.7626747729	1.7626747758	2.86×10^{-9}
ZnS (zincblende)	1.6380550534	1.6380550533	3.41×10^{-11}
2D square lattice	1.6155426267	1.6155426267	1.24×10^{-11}

The zincblende ZnS entry uses the sphalerite Madelung constant normalized by the nearest-neighbor separation, 1.63805505338879. Comparison against the rounded value 1.6380 would give an artificial 5.51×10^{-5} residual.

S4.5 Comparison against published reference values

Beyond the matched out-of-process reference runs, the periodic test systems are also compared against values published in the literature. The pob-TZVP basis-set paper [5] tabulates periodic total energies per unit cell for a set of cubic ionic solids computed with Crystal, at both the Hartree–Fock and the PW1PW level, for the Crystal standard basis sets and for pob-TZVP. Two of those compounds, LiH and NaCl, are also part of the periodic test set of this paper, which makes them a direct external check that is independent of the reference programs executed here.

Table S19 reproduces the published values and pairs them with the corresponding vibe-qc results. Reproducing these numbers requires the pob-TZVP basis and the matching lattice constants and truncation settings of the original work, so the vibe-qc column is filled from dedicated pob-TZVP runs (bipolar-expansion route, $(2, 2, 2)$ mesh, real-space cutoffs of 20–25 a_0, energy convergence 1×10^{-9} E_h) rather than from the STO-3G rows of Table S15. The vibe-qc values agree with the published Crystal values to between 1.0 mE_h and 19.6 mE_h. These are external consistency checks rather than parity claims: the two codes differ in their lattice-sum truncation schemes, and the quoted values are not converged with respect to the real-space cutoff, whose confirmation runs did not complete. The agreement is nevertheless three orders of magnitude tighter than the held STO-3G dense-core rows of Table S15, and the reason is the route rather than the system: these rows are computed on the bipolar-expansion route, which sums the two-electron lattice sum directly and so never forms the Gaussian density fit whose truncation produces that deficit. The same systems are not immune to it. Measured after this table was written, all-electron pob-TZVP-rev2 cells taken through the density-fitting route at the tested default cutoffs sit in the held class themselves, and the steep core functions of the heavier cations put them among the worst affected, so the deficit scales with the basis's steepest exponent rather than with the nominal quality of the basis. The corresponding MgO calculations did not complete within their allocated wall time and are not reported.

Table S19: Periodic total energies per unit cell (E_h) compared against the published values of SI Ref. [5], which were obtained with Crystal. HF denotes Hartree–Fock; the PW1PW columns use the same hybrid functional as the original work. vibe-qc values use the pob-TZVP basis and the published structures.

	HF		PW1PW	
Compound	Ref. (pob-TZVP)	vibe-qc	Ref. (pob-TZVP)	vibe-qc
LiH	−8.060732	−8.059734	−8.149050	−8.129497
NaCl	−621.504227	−621.519103	−622.617190	−622.624367

S5 Per-job artifacts

Each job writes calculation results, metadata, and citations. This section shows the provenance and citation artifacts for a representative molecular job; the structure is identical for periodic jobs.

S5.1 Provenance manifest (`.system`)

The `.system` file is a TOML manifest pinning the build, host, and runtime so a reported result is traceable to an exact code state. A representative manifest is shown below (illustrative format; the live values are filled at run time).

Listing S1: Representative `.system` manifest.

```
[build]
version = "<filled at run time>"
commit = "<filled at run time>"
libint = "2.13.1"
libxc = "7.0.0"
spglib = "2.7.0"
libecpint = "1.0.7"

[host]
cpu = "<filled at run time>"
cores = "<filled at run time>"
memory_gb = "<filled at run time>"
os = "<filled at run time>"

[run]
omp_threads = "<filled at run time>"
fingerprint = "<filled at run time>"
```

S5.2 Automatic citation manifest (`.references` / `.bibtex`)

Each job assembles, from a single citation database, the list of papers for every method, functional, basis set, ECP, and linked library it used, and writes them as a human-readable `.references` file and a ready-to-use `.bibtex` file. For the closed-shell PBE0-D3(BJ)/def2-TZVP job (M07) the manifest contains, among others, the def2 basis [6], the PBE0 functional [7, 8], the D3(BJ) correction [9, 10], libint [11], and libxc [12]. The files identify the references associated with the methods used in the calculation.

S5.3 The qvf visualization archive

Every job writes a qvf archive by default (`output_qvf` defaults to true). The archive follows specification v1.2; the manifest integer stays at 1 across the v1.x line, and a periodic section is an optional member rather than a version bump. It bundles, as available, the structure, molecular orbitals, density, band structure and density of states, spectra, and optimization trajectory. The vibe-view viewer reads the archive directly and renders each component interactively in the browser. The archive is a versioned container so that older files remain readable as the schema evolves. The conventional formats (`.molden`, extended XYZ, POSCAR, XSF, BXSF, CIF, Gaussian cube) are written alongside for interoperability, and wavefunction data intended for another program can optionally be written as a trexio file (main text, Section 3.2).

S5.4 TREXIO implementation and validation

The molecular SCF writer and reader support HDF5 and text backends through the official TREXIO library [13]. TREXIO output is opt-in; QVF remains the default result archive. The previously archived H_2O/RHF and OH/UHF def2-SVP checks recover orbital orthonormality to 3×10^{-15}. Independent PySCF energy reconstruction from the stored orbitals and integrals differs by $2.4 \times 10^{-13}\,E_\mathrm{h}$ and $1.7 \times 10^{-13}\,E_\mathrm{h}$, respectively. These tests concern molecular all-electron SCF wavefunctions with

spherical Gaussian AOs. They do not establish ECP, reduced-density-matrix, periodic, complex-orbital, or file-seeded calculation support.

The expanded TREXIO implementation was tested in development version `0.17.3.dev0`. Automatic export includes molecular and complex periodic SCF orbitals with spin, occupations, and $\boldsymbol{k}$ metadata; applied scalar ECPs; molecular one-particle density matrices; and CASCI/CASSCF/FCI determinants in their actual orbital basis, including frozen core and the selected state. Molecular and periodic READ restarts check the target basis, spin populations, and mesh. Native basis reconstruction requires real spherical Gaussian primitives with zero radial power. General data transport also handles other basis representations, sparse data, and explicitly supplied higher density matrices or correlation amplitudes; these are not all automatic solver exports. Multi-$\boldsymbol{k}$ AO matrices are omitted, and periodic optimization wavefunction sidecars describe the initial SCF.

The retained producer test report contains 149 passing checks with TREXIO 2.6.1 and PySCF 2.14.0, including independent-integral ECP and CASSCF reconstructions. This is development-build evidence, separate from the v0.17.1 numerical campaign. The viewer reader imports geometry and real nonperiodic Gaussian orbitals through f, but refuses periodic or complex orbital rendering and higher angular momenta. General file transport does not imply support for every visualization.

Tests of file exchange between the calculation code and viewer passed for H_2O/RHF and OH/UHF with def2-SVP, using both HDF5 and text. Every molecular orbital was compared at 27 asymmetric points; the largest absolute orbital-value difference was below 3.34×10^{-16} in atomic units. Geometry, orbital energies, and QVF member checksums were also checked. These establish conversion consistency; independent energy reconstruction is assessed separately. Source TREXIO files, converted QVF archives, validation records, and exact build identities are retained together. These are development-build checks, not claims about an unverified public release tag.

The rendering example uses a converged H_2O/RHF def2-SVP result from the v0.17.3 candidate, written as HDF5 TREXIO by VIBE-QC. Opening that file directly in VIBE-VIEW v2.17.1 renders the HOMO at $|\psi| = 0.05$ atomic units on a $60 \times 60 \times 60$ grid, with positive and negative lobes. The calculation fixture, unchanged TREXIO file, imported QVF, version records and unmodified browser screenshot are supplied in the `trexio-water-render` supplement. The producer candidate and original viewer v2.17.1 build are identified in its `example.json`; neither is represented as a verified public tag at this cutoff. Figure S1 shows the retained display.

S6 The VIBE-QUEUE job scheduler

The calculations were submitted and monitored with VIBE-QUEUE (command `vq`), which is maintained as a separate project (main text, Section 3.2). The scheduler also collected the results and resource-use records. Unit and interoperability tests were run separately.

Each job receives its own workspace and scratch directory, and its declarative specification is written to disk before any process starts, so a restart of the scheduler reattaches a running job rather than running it again. On a workstation a watchdog samples the resident memory and CPU usage of each job, enforces its requested limits, and records the peak memory, the wall time, and the terminal reason with the job.

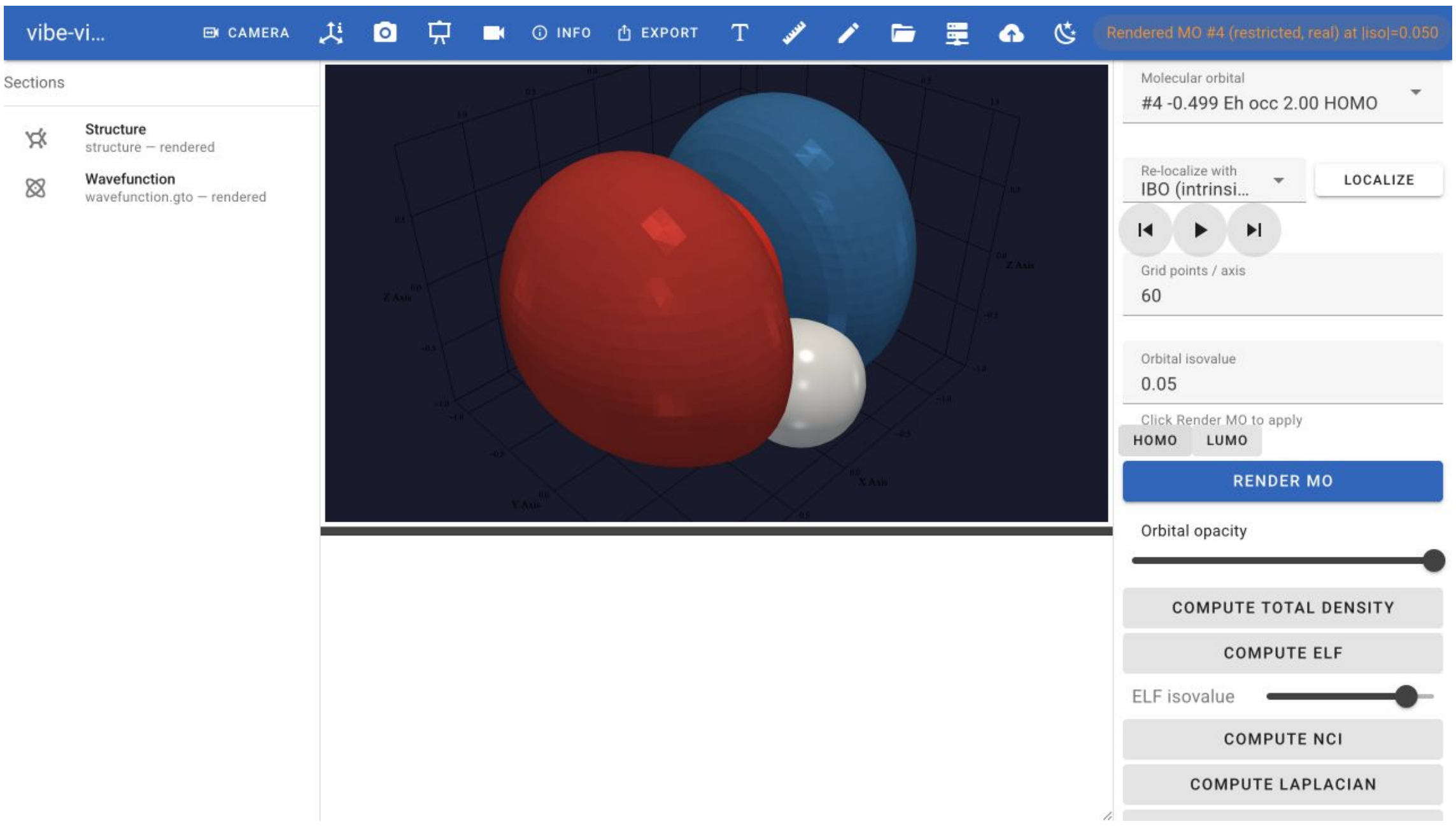

Figure S1: Recorded VIBE-VIEW display after opening the actual water RHF/def2-SVP HDF5 TREXIO output. Blue and red lobes show the signs of the HOMO at $|\psi| = 0.05$ atomic units, sampled on a 60^3 grid. The unchanged screenshot is part of the supplementary example.

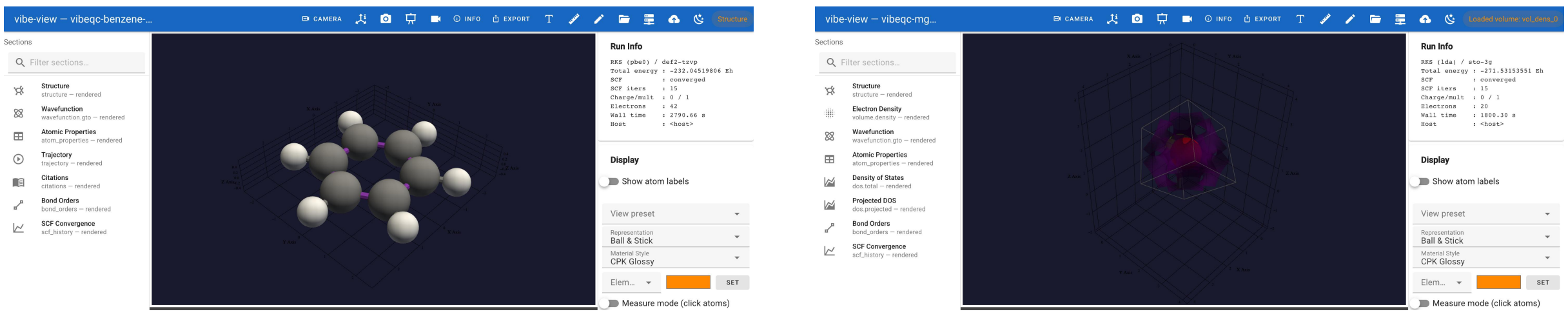

Figure S2: The VIBE-VIEW browser viewer. Left: the optimized structure of benzene (M20, RKS PBE0/def2-TZVP) with CPK spheres and inferred bonds. Right: the electron density of MgO (KRKS LDA/STO-3G, GDF, $k = (2, 2, 2)$) as an isosurface inside the periodic cell. Both are unmodified screenshots captured from archives produced by the release-paper calculation set.

On the university clusters used here, one PBS/Torque and one Slurm system, the same specification is translated into a batch submission and the site scheduler enforces the limits. The job still records the same event log, and its workspace is fetched back with the `.system` manifest, the QVF archive, the output files, and the scheduler logs. Each result retains its calculation identifier.

A twenty-water RHF/STO-3G probe converged in 19 iterations with either 64 or 128 OpenMP threads. Both runs gave

$$E = -1499.343\,640\,976\,0\,\mathrm{E_h}.$$

This checks OpenMP execution and scheduler integration. It is not a scaling study or evidence of production MPI execution.

S7 Input examples

The following examples illustrate the molecular and periodic entry points; they are API examples, not additional validation results.

Listing S2: The high-level API. The same `Molecule` drives every molecular method; the periodic route is selected by one keyword.

```
import vibeqc as vq

# Molecule: a closed-shell DFT single point with dispersion.
mol = vq.Molecule([vq.Atom(8, [0.0, 0.0, 0.0]),
 vq.Atom(1, [0.0, 1.43, -0.98]),
 vq.Atom(1, [0.0, -1.43, -0.98])])
vq.run_job(mol, basis="def2-tzvp", method="rks", functional="pbe0",
 dispersion="d3bj", output_qvf=True)

# Crystal: multi-k periodic HF via Gaussian density fitting.
import numpy as np
a = 7.72
lat = (a/2) * np.array([[0,1,1],[1,0,1],[1,1,0]])
sysp = vq.PeriodicSystem(3, lat, [vq.Atom(3, [0,0,0]),
 vq.Atom(1, [a/2, a/2, a/2])])
basis = vq.BasisSet(sysp.unit_cell_molecule, "sto-3g")
vq.run_periodic_job(sysp, basis, method="RHF",
 jk_method="gdf", kpoints=(2, 2, 2))
```

S8 The validation database

This section summarizes the numerical comparison records as UTF-8 CSV and newline-delimited JSON, with a column schema and checksums. It contains 3861 registered cases, 8433 associated runs, and 5839 numerical comparisons. The scripts distributed with the tables reproduce the summary tables and statistical classifications. Incomplete attempts, one-sided reference measurements without a paired result, superseded records, and work outside the paper's scope are omitted. Separate qualification records exclude results with a disproven molecular identity while preserving the original values, verdicts and input hashes. Completed comparisons with valid identities that miss a tolerance remain in the data.

Some historical comparisons name case identifiers without a separate case metadata record; these identifiers and missing joins are listed in the results schema. All comparison-to-run joins resolve. The supplement preserves the measured values and available settings for reanalysis. Historical empty metadata fields remain unrecorded; they are not inferred from a filename or a newer release. Repository-relative source locators describe the retained evidence record and do not promise that the native files are included. Selected printed tables are also supplied as editable rows with their labels, values, units, and notes. The package does not claim complete calculation rerun reproducibility.

Verdicts. Table S20 gives the verdicts of the supplied comparisons, split into molecular and periodic rows.

Table S20: Verdicts of the comparisons in the deposited validation database. Each comparison contains a measured difference or two finite operands. Every row, with its recorded reason, is in the machine-readable database (Section S8).

Verdict	Molecular	Periodic
pass	3328	1
pass-pending-tolerance	158	76
held	1002	71
fail	1111	77
recorded-no-tolerance-class	9	6
Total	5608	231

Reading the verdict column. The verdict records the numerical outcome of a comparison. `pass` and `fail` score the deviation against the recorded tolerance. A missed tolerance is not itself proof of a method defect. A recorded verdict also does not certify convergence or complete provenance; those conditions are distinct from the numerical verdict. `held` means both codes completed but the comparison is retained under a stated convention, gauge, or by-design exclusion rather than scored. `pass-pending-tolerance` means the deviation is recorded but no tolerance class has been fixed for the quantity. `recorded-no-tolerance-class` records a paired numerical value without a verdict.

Coverage. The database contains 5839 comparisons. Of those, 3329 pass (57.0 %), 1188 fail (20.3 %), 1073 are held (18.4 %), and 249 carry paired numerical values without a scored verdict. Table S21 gives the same counts per method family. Deviations with different physical quantities or units are not pooled.

The meaning of a passing comparison depends on the reference. Of the 3329 passing rows, 1570 (47 %) agree with an independent program within a parity tolerance; 1263 (38 %) belong to the published-benchmark sets, where the recorded tolerance is a historical outlier threshold; both passing and failing scored entries enter the accuracy statistic; 451 (14 %) compare VIBE-QC against itself, including reproducibility tests across releases and hosts; and 45 are against a stored two-code consensus value. Only the first group is cross-code parity. Each comparison is classified using the associated reference run. Classification by case name alone would incorrectly assign some reproducibility tests to the published-benchmark group.

Most program comparisons concern molecular methods, with one reference program selected for each method family. Of the 1570 cross-code passing rows, 1535 (98 %) are against ORCA, 34 against OPENMOLCAS for the complete-active-space family. The three periodic reference programs, PYSCF, CRYSTAL 23 and GPAW, carry 1 passing row. One periodic cross-code total-energy comparison now exists, RHF LiF in the pob-TZVP-rev2 basis on a $2 \times 2 \times 2$ mesh, the bipolar route against PYSCF with both legs correcting the exchange divergence by an Ewald term; it agrees to 0.25 mE_h and passes the periodic total-energy class of 0.5 mE_h. The floor that class rests on is not the grid-converged DFT reference-to-reference spread above, which does not transfer to an exchange-divergence comparison; the relevant uncertainty is the reference's own density-fitting error. Across 9 PYSCF legs on the same cell and mesh that differ only in the auxiliary basis, the reference energy spans 0.12 mE_h and moves toward the bipolar-route value as the fit is densified. Two independent routes to the fitting limit bracket it. An even-tempered Gaussian ladder flattens near 0.100 mE_h below the registered reference, and mixed density fitting, which adds a plane-wave

correction to the fitted Coulomb metric, lands 0.019 mE_h further at three plane-wave meshes that agree with each other to 0.0006 mE_h. The reference's own fit error on this cell is therefore 0.12 mE_h with an uncertainty of 0.02 mE_h, the separation between the two routes, and the remaining difference between implementations is 0.13 mE_h, not the 0.25 mE_h of the registered pair. The class is therefore set above the reference's own fit error rather than fitted to the registered pair.

Most molecular comparisons use Orca as the reference. The molecular mean-field, DFT, perturbation-theory, active-space, and excited-state families are densely covered. The periodic families have a single scored pass: their other outcomes are held or failed under the conventions of Section S4, and the two closed comparisons that the main text reports for the density-fitting route at the Γ point are ladder measurements registered as runs rather than as scored rows.

Table S21: Validation records by method family. Other denotes paired numerical records without a scored verdict. Deviations are not pooled across different quantities or units.

Family	Total	Pass	Fail	Held	Other
Hartree–Fock	195	165	24	0	6
Self-consistent field	790	406	378	0	6
Kohn–Sham DFT	1223	912	173	38	100
Noncovalent DFT	616	85	3	528	0
Dispersion-corrected DFT	5	3	2	0	0
Double hybrids	7	4	3	0	0
Møller–Plesset	327	248	52	22	5
Coupled cluster	268	109	128	5	26
Local correlation (DLPNO)	637	189	179	267	2
Active-space CI	30	30	0	0	0
Multireference perturbation theory	12	10	2	0	0
Other post-Hartree–Fock	13	6	7	0	0
Excited states	579	352	77	140	10
Electron propagator	112	93	19	0	0
Molecular properties	243	213	28	1	1
Add-on corrections	29	10	19	0	0
Periodic: Gaussian density fitting	9	0	9	0	0
Periodic: bipolar expansion	88	1	1	4	82
Periodic: Gaussian and plane waves	134	0	67	67	0
BH9 barrier heights	489	479	10	0	0
Other	33	14	7	1	11
All families	5839	3329	1188	1073	249

Comparisons exceeding the tolerance. A failing numerical record misses the tolerance its case declared. The failures are not uniform, and Tables S22 and S23 give two views of them: by the tolerance class the row was scored against, and by the size of the deviation. Read together with the recorded reasons, which `scripts/validation_taxonomy.py` classifies, the 1188 failures fall into the following groups.

- 213 *matched-reference DFT reruns* from deck family `dft-grid-default-comparison`. The original comparisons used different integral approximations and integration grids. For the Coulomb

and exchange terms, the original Orca decks carry no RI keyword, so Orca 6.1.1 applied its default Split-RI-J for the GGAs and RIJCOSX for the hybrids while vibe-qc computed the exact four-index Coulomb and exchange terms. Re-running the family with density fitting disabled on the Orca side removes about 92 % of the deviation, identifying density fitting as the dominant contribution. For the exchange–correlation quadrature, convergence tests on two cases separate the remaining difference into each program's grid error and a residual between the two calculations. Grid-free Hartree–Fock controls on the same geometry and basis agree to a few nanohartree. These pairs were scored against the $1 \times 10^{-8}\ E_\mathrm{h}$ class that the SCF-limited rows use, and the matched rerun does not bring them inside it.

These comparisons have two limitations. First, the vibe-qc calculations did not use the shipped default grid: the staged inputs pass an empty options object, and a defect in the runner keys the grid selection on whether an object was passed rather than on what it contains, so the runs took the legacy preset instead. Those rows therefore compare a legacy grid against Orca DefGrid3, and no statement about the shipped default may be drawn from them. Second, a $1 \times 10^{-8}\ E_\mathrm{h}$ tolerance is too tight for a τ-meta-GGA against a reference computed at DefGrid3, because the reference's own energy there is some hundreds of microhartree from its converged value and still moving. Both are tracked in the issue tracker.

- 61 *matched DFT comparisons* (DefGrid3 on both sides, conventional integrals on both sides after the Orca decks were amended with `NORI`) that miss the $5 \times 10^{-5}\ E_\mathrm{h}$ class, with a median deviation of $8.7 \times 10^{-5}\ E_\mathrm{h}$ and a maximum of $2.9 \times 10^{-4}\ E_\mathrm{h}$. These residual differences remain unresolved.
- 1 additional comparison uses unmatched default grids and is reported separately from the registered NORI rerun family.
- 95 rows of the *open-shell DFT ladder* at the $5 \times 10^{-5}\ E_\mathrm{h}$ class, median $7.8 \times 10^{-5}\ E_\mathrm{h}$, together with 33 held rows of the same ladder. They are held under a tracked convergence-floor defect of the unrestricted DIIS path and are diagnosis-only by design.
- 93 *controlled internal comparisons* of vibe-qc against vibe-qc (local against canonical correlation, dimer against monomers), which measure a truncation and are recorded as fails because the truncation exceeds the class, not because two programs disagree.
- 92 *solvation response components*, scored component by component at the $1 \times 10^{-3}\ E_\mathrm{h}$ class with a median deviation of $1.6 \times 10^{-3}\ E_\mathrm{h}$ (Section S3.3).
- 58 *band-gap rows* on the experimental GPW route and 17 rows carrying a registered route defect, all periodic and all outside the claims of the paper.
- 29 *auxiliary-basis mismatches* in the MP2 family and 23 rows of a tracked DLPNO-CCSD solver defect.
- 19 OVGF rows that record method accuracy against an IP-EOM-CCSD reference and were never parity targets.
- The remaining 487 rows are individually recorded: correlated totals scored at the $1 \times 10^{-5}\ E_\mathrm{h}$ class

whose frozen-core or density-fitting settings were later matched in a registered rerun (the reruns are the values the paper quotes), the QUESTDB excitation energies outside their accuracy band, historical records requiring provenance reconciliation, and one ECP-labeled row whose VIBE-QC leg ran all-electron (Section S3.1).

The deviation-magnitude and tolerance tables below include only quantities explicitly reported in hartree. Excitation energies, barriers, gradients, charges, and other units remain in the machine-readable tables and are not combined with those energy statistics. Among the 885 failing hartree-valued comparisons, 448 lie within 1×10^{-4} E_h of their reference and 582 within 1×10^{-3} E_h.

The tolerances must be interpreted together with the convergence of the reference calculations. In the comparisons above, two reference programs differ by tens of millihartree for a periodic meta-GGA total energy. A molecular reference on a standard integration grid can also differ by hundreds of microhartree from its grid-converged value. These reference errors limit the precision with which agreement between implementations can be assessed. Some historical tolerances were assigned before these convergence tests; the corresponding results are therefore reported with their numerical deviations and convergence limitations.

Table S22: Tolerance classes for quantities explicitly reported in hartree. Other units and unit-ambiguous records are excluded. Tolerances are the recorded values, not rounded classes. A missed tolerance does not alone identify an implementation defect.

Tolerance class / E_h	Pass	Fail	Scored	Pass rate
1×10^{-12}	2	0	2	100%
1×10^{-9}	215	0	215	100%
1×10^{-8}	57	220	277	21%
5×10^{-8}	2	0	2	100%
1×10^{-6}	448	64	512	88%
2×10^{-6}	22	0	22	100%
3×10^{-6}	4	0	4	100%
4×10^{-6}	4	0	4	100%
5×10^{-6}	2	0	2	100%
6×10^{-6}	4	0	4	100%
8×10^{-6}	2	0	2	100%
1×10^{-5}	1	0	1	100%
1×10^{-5}	138	239	377	37%
5×10^{-5}	375	157	532	70%
0.0001	3	4	7	43%
0.000 15	9	0	9	100%
0.000 155	5	0	5	100%
0.0005	65	5	70	93%
0.001	181	106	287	63%
0.005	115	79	194	59%
0.0055	1	0	1	100%
no numeric tolerance	0	11	11	0%

Unscored comparisons. The 1073 held rows are dominated by three families of controlled data rather than by unresolved comparisons: 528 S66x8 interaction energies computed without counterpoise correction in def2-SVP against the counterpoise-corrected complete-basis-set reference, which are method-accuracy data and not parity data; 267 DLPNO-MP2 and DLPNO-CCSD(T) pair-natural-orbital threshold

Table S23: Failing comparisons binned by the absolute deviation from the reference, restricted to quantities explicitly reported in hartree. Other units are excluded.

$\|\Delta\|$	Rows	Share of fails
$< 10^{-6}$	25	3%
10^{-6} to 10^{-5}	85	10%
10^{-5} to 10^{-4}	338	38%
10^{-4} to 10^{-3}	134	15%
10^{-3} to 10^{-2}	219	25%
$\geq 10^{-2}$	84	9%
no numeric deviation recorded	0	0%
All fails	885	100%

ladders measured against the canonical result on both sides (the truncation errors quoted in the main text are drawn from them); and 130 QUESTDB entries excluded by principle, the doubly excited states and the fluorescence entries. The remainder are 70 sampled band gaps without an assigned tolerance class, 33 open-shell ladder rows, 27 sanity-band rows, and 18 rows held under a stated convention or gauge, including the periodic total energies of Section S4.

S9 Implementation details

This section collects the implementation detail that supports the code description of the main text: the per-job artifact bundle and the run-time citation mechanism, the documentation and tutorials, and the development toolchain under which the code was written.

S9.1 Reproducibility artifacts and automatic citations

The output and citation formats are described in Section S5. The bibliography uses a snapshot of the software citation catalogue plus additional methodological references. Citation-key and bibliography checks keep the paper's references consistent with the methods described here.

S9.2 Documentation, examples, and tutorials

VIBE-QC ships documentation, examples, and tutorials at `https://vibe-qc.com`. Beyond the API and user-guide reference, the tutorials are written to be read in sequence as an introduction to quantum chemistry. They progress from Hartree–Fock and basis sets through DFT, perturbation theory, coupled cluster, and multireference methods to periodic systems, each step pairing the theory with a runnable VIBE-QC input and an interpretation of the output.

S9.3 Development toolchain

The code was written with generative-AI assistance under direct human supervision. The rationale and the division of labor are the subject of Section 6.1 of the main text. The models were used in distinct roles: Anthropic Claude Opus 4.8 [14] (and Opus 4.7 earlier) [15], later joined by Claude Opus 5 and Claude Fable 5 [16, 17], as the main development chats, DeepSeek [18, 19] and later DeepSeek v4 Pro [20]

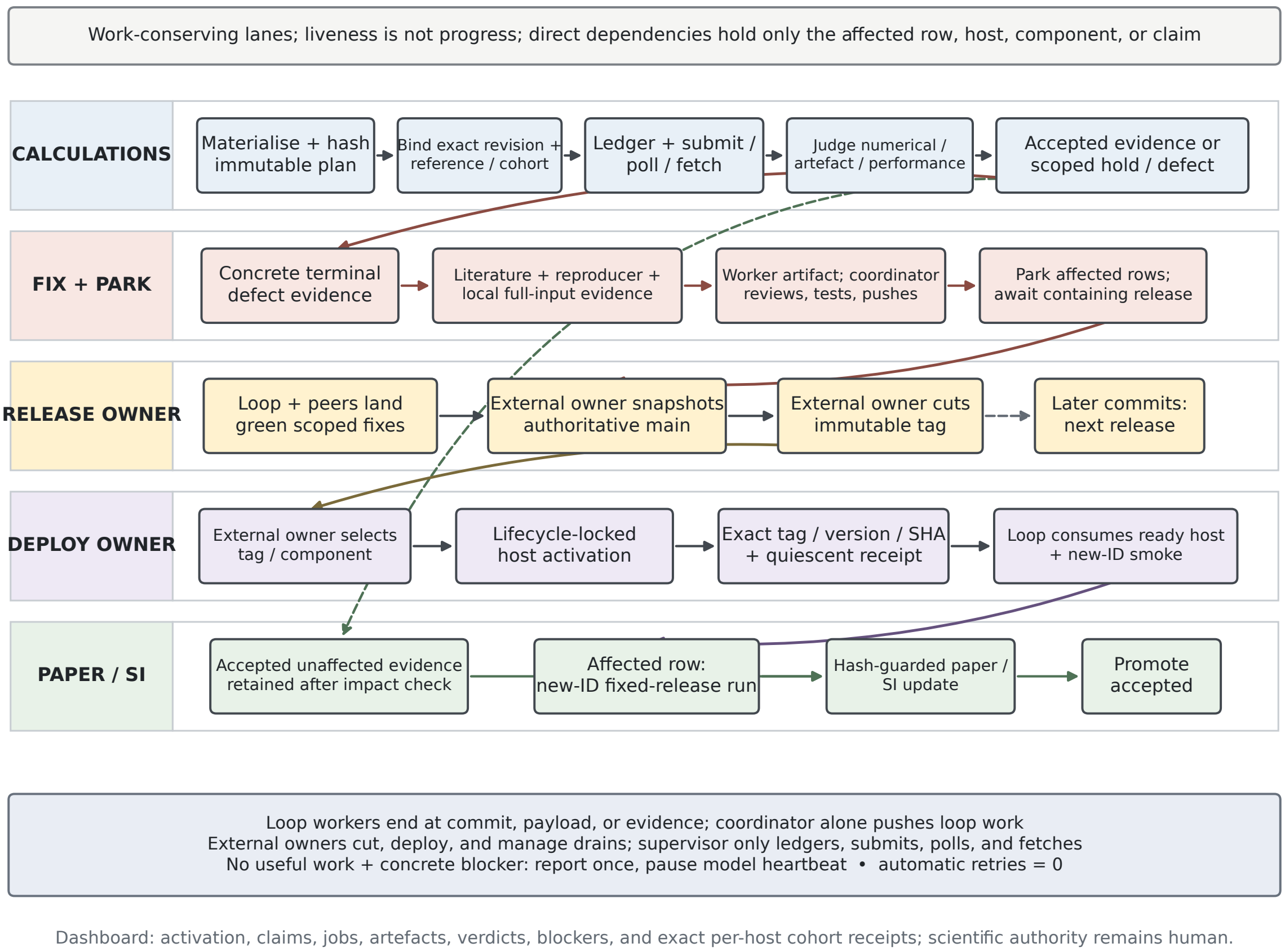

Figure S3: Concurrent validation tasks. The horizontal rows represent calculation, result collection, comparison, defect correction, and reporting. Arrows indicate dependencies. Calculations affected by a confirmed defect wait for correction and repetition; other calculations continue. The author controls activation and scientific decisions.

for high-volume routine generation, and OpenAI Codex 5.5 [21] together with ChatGPT 5.6 Sol [22] for debugging and refactoring, with the models frequently cross-checking one another's output.

Calculation submission, result collection, comparison with references, and defect correction were coordinated by an agentic controller. Independent tasks ran concurrently. A calculation affected by a confirmed defect waited for a corrected release, while other calculations continued. The controller required explicit activation and stopped when further work depended on unavailable inputs or unresolved defects. Scientific decisions remained with the author.

Before an energy comparison, the settings were checked for consistency: method, basis and ECP, charge, multiplicity, frozen-core treatment, quadrature, density fitting, integral approximation, symmetry, $\boldsymbol{k}$ sampling, and solver thresholds. For example, a water MP2 comparison initially used frozen-core orbitals in the reference and all-electron correlation in VIBE-QC. A matched reference calculation was required before assessing the difference.

The procedure retained program outputs, manifests, QVF records, and source identities. Corrected inputs received new calculation identifiers; automatic retries were disabled. Performance comparisons required matched hardware, resource allocations, and algorithms. Literature values were checked against the local reference library. Reference programs ran as separate processes. Figure S3 shows the tasks and their dependencies.

Table S24: Release history of VIBE-QC (patch releases omitted), with the foundational work each release's principal capability builds on.

Release	Date	Work the principal capability builds on
0.1.0	2026-04-18	Roothaan–Hall equations (C. C. J. Roothaan)
0.2.0†	—	Ewald summation (P. P. Ewald)
0.2.5†	—	Wigner D-matrices and group theory (E. Wigner)
0.3.0†	—	Molecular-orbital theory (R. S. Mulliken)
0.4.0	2026-04-27	Hellmann–Feynman theorem (H. Hellmann)
0.5.0	2026-04-30	FG-matrix vibrational analysis (E. B. Wilson)
0.6.0	2026-04-30	DIIS and analytic gradients (P. Pulay)
0.7.0	2026-05-02	Canonical orthogonalization (P.-O. Löwdin)
0.8.0	2026-05-17	DFT-D3/D4 dispersion (S. Grimme)
0.9.0	2026-05-23	Coupled cluster (P. J. Knowles)
0.10.0	2026-05-27	Periodic Hartree–Fock (C. Pisani)
0.11.0	2026-06-03	Range-separated density fitting (Q. Sun)
0.12.0	2026-06-11	Algorithmic analysis (D. E. Knuth)
0.13.0	2026-06-17	Generalized-regular $\boldsymbol{k}$ grids (P. Wisesa)
0.14.0	2026-06-22	CCSD(T) (R. J. Bartlett)
0.15.0	2026-06-26	DLPNO-CCSD(T) (F. Neese)
0.16.0	2026-09-08	Ab initio methods made usable by anyone (J. A. Pople)
0.17.0	2026-09-09	Groundwork for periodic local correlation (D. P. Tew)

† `0.2.0`, `0.2.5`, and `0.3.0` were internal milestones that were not separately tagged.

Standard build-time tooling (CMake, scikit-build-core, `pytest`, Sphinx) and a GitLab continuous-integration pipeline complete the toolchain. Development changes are checked with the relevant local tests and representative calculations. The release pipeline tests clean builds and integration. Regression tests that exceed the shared runner's memory are executed on a development workstation before a tagged release.

S9.4 Development chronology

S10 Method support and methodological details

Method-support descriptions refer to the original v0.17.1 source audit; the numerical comparisons retain their recorded build identities. These tables describe the methods for which this paper discusses numerical evidence. Availability of an energy does not establish availability of its derivatives. "Verified" refers to the documented cases, not to every combination of basis, spin, approximation, and system size. Finite-difference (FD) derivatives must use the requested method's own energy or, where appropriate, free-energy surface. Local-correlation methods are identified as pilot implementations. GPW/GAPW remains experimental and is not validated for production use. The restrictions in the tables and main-text Section 6.3 apply to the reported method and derivative support.

Table S25: Molecular methods with evidence discussed in this paper. R, U, RO: the method runs on a restricted closed-shell, an unrestricted, or a restricted open-shell reference. CAS and FCI spatial-orbital conventions are specified separately in footnote t. E: single-point energy. ∇: nuclear gradient, analytic (A) or finite-difference (FD). H: nuclear Hessian. RI: density fitting of the Coulomb and exchange builds (RI-J, RI-JK) or of the correlation integrals. COSX: RIJCOSX exchange with the GRID-X grid tiers. • available, ○ available with the stated qualification, – not available within the stated scope; derivative entries marked with superscript r are unclaimed rather than an assertion that every helper is absent. Verified denotes evidence on the stated cases; pilot denotes a measured implementation with restricted scope. The archived evidence behind each verified row is in Sections S3 and S4.

Method	R	U	RO	E	∇	H	RI	COSX	Status
Mean field									
RHF	•	–	–	•	A	FD[a]	•	•	verified
UHF	–	•	–	•	A[b]	FD[a]	•	•	verified
ROHF	–	–	•	•	A	FD	•	○[c]	verified
RKS (LDA, GGA, meta-GGA, global hybrids)	•	–	–	•	A[d]	FD[a]	•	•	verified
UKS	–	•	–	•	A[b,d]	FD[a]	•	•	verified
ROKS	–	–	•	•	FD	–	○[e]	○[c]	verified
Range-separated hybrids (ωB97X, ωB97X-V, ωB97M-V, CAM-B3LYP, HSE06), VV10	•	•	•	•	FD[f]	–	–	–	verified
Corrections and composite methods									
DFT-D3(BJ)	•	•	•	•	A	–			verified
DFT-D4	•	•	•	•	A[g]	–			verified
CPCM continuum solvation	•	•	–	•	A[h]	–			verified
HF-3c	•	–	–	•	○[i]	–			verified
Perturbation theory and double hybrids									
MP2, SCS-MP2, SOS-MP2	•	•	•	•	–[r]	–[r]	•[j]	○[k]	verified
B2PLYP	•	–	–	•	–[r]	–[r]	•	○[k]	verified
Coupled cluster									
CCSD, CCSD(T)	•	•	•	•	–[r]	–[r]	•[l]	○[k]	verified
FNO-CCSD(T)	•	–	–	•	–[r]	–[r]	•	○[k]	verified
DLPNO-MP2, DLPNO-CCSD, DLPNO-CCSD(T)	•	•	–	•	–[r]	–[r]	•	○[k]	pilot
Multireference									
CASCI	spatial[t]			•	FD[u]	–[r]	–	–	verified
CASSCF, SA-CASSCF	spatial[t]			•	A/FD[m]	–[r]	–	–	verified
NEVPT2 (strongly contracted)	spatial[t]			•	FD[v]	–	–	–	verified
FCI	spatial[t]			•	–[r]	–	–	–	verified
Excited states and propagators									
TDDFT (Casida, TDA), CIS	•	•	○[q]	•	–[s]	–	•	○[k]	verified
GF2, OVGF ionization energies	•	•	–	•	–[r]	–	–	–	verified

[a] The reported frequency-driver path differentiates gradients by finite differences. The ASE UKS path is also finite-difference. Routines labelled analytic contain numerical response steps or incompletely audited response terms and do not establish general analytic Hessian support. [b] Open-shell *f*-shell gradient accuracy has not been revalidated at the numerical baseline; the historical

defect report is not an enforced API guard. ROHF gradients refuse ECP and DF+COSX combinations. [c] RIJCOSX energies only. The RIJCOSX gradient fails closed for this reference. [d] LDA, GGA, global hybrids, and τ-meta-GGA functionals. Range-separated hybrids are listed separately. [e] Density fitting is reachable through the ROKS options for functionals without range separation. [f] Range-separated hybrids and VV10 run on the direct four-index path. Their analytic gradient is not implemented (the long-range exchange and the VV10 nonlocal terms are missing); the public gradient API refuses them, and geometry optimization with these functionals runs on central finite differences of the total energy. [g] Analytic through the reference `dftd4` backend. The native backend carries a partial derivative. [h] Analytic through the lower-level API and the ASE calculator. The `run_job` optimizer does not yet carry the term. [i] The composite gradient omits the short-range basis and counterpoise terms. Optimization on the composite surface is not offered. [j] RI-MP2 for restricted and unrestricted references. ROHF-MP2 is RI only. [k] RIJCOSX accelerates the reference SCF only. [l] Density-fitted by default. The conventional four-index route is selected by option and is the cross-code parity route. [m] The native optimizer admits analytic CASSCF for a singlet, one root and no numerical-gradient reference. Other native cases and the ASE CASSCF path use total-energy finite differences. [q] TDA only, on an ROHF reference. [r] The derivative indicated by this column is not claimed here. MP2, CCSD/(T), and DLPNO optimization and frequency requests pass the mean-field reference method to the derivative driver and add correlation afterward. Separate helpers require method-specific evidence. No correlated Hessian is validated here. [s] The optional excited-state finite-difference driver is restricted to closed-shell RHF/TDA (HF-CIS) at the numerical baseline. Its coordinate unit handling requires verification in a calculation. No excited-state gradient result is validated in this paper. [t] CAS methods use common spatial orbitals from RHF, UHF, UNO, or ROHF; the defaults are RHF for closed shells and UNO for open shells. This is not an unrestricted correlated-orbital formalism. FCI uses a separate determinant solver with RHF/UHF-derived orbitals. [u] CASCI uses total-energy finite differences through both the native optimizer and the ASE wavefunction path. [v] The native MRPT optimizer has a method-specific energy path. The ASE NEVPT2/CASPT2/FCI fallback uses mean-field forces and is excluded from the correlated-derivative scope.

Table S26: Periodic routes and corrections with evidence discussed in this paper. Dim.: dimensions with an energy route. Γ and $\boldsymbol{k}$: single-point energies at Γ and on admitted meshes. These entries do not imply every combination of reference, dimension, fitting variant, mesh, and derivative. XC: available functional families. ∇: nuclear gradient at fixed cell. Symbols as in Table S25. Variable-cell optimization is refused on the reported GDF and BIPOLE routes; no validated stress tensor is claimed.

Route or method	References	Dim.	Γ	$\boldsymbol{k}$	XC	∇	Status
GDF (range-separated and mixed density fitting)	RHF, RKS, UHF, UKS[a]	1, 3; 2 closed-shell	•	•	LDA, GGA, meta-GGA, global hybrids	A/FD[b]	verified at Γ^e; under review at multiple $\boldsymbol{k}$
BIPOLE (bipolar expansion, corrected Ewald gauge)	RHF, UHF, RKS, UKS[a]	3	•	•	LDA, GGA, meta-GGA, hybrids	FD[c]	selected Γ and LiF multi-$\boldsymbol{k}$ checks; other totals diagnostic
Periodic DFT-D3(BJ)	all routes	1, 2, 3	•	•		A[d]	verified

[a] Three-dimensional energy previews additionally admit ROHF on GDF and ROHF/ROKS on BIPOLE; GDF refuses ROKS. Their public gradients and optimization are refused. [b] Selected fixed-cell routes only; bulk multi-$\boldsymbol{k}$ KS gradients include a numerical moving-grid XC contribution. See Section S10.11 for fitting, mesh, reference, and occupation restrictions. [c] Production forces use central differences of the converged total energy at $T = 0$ or Mermin free energy with positive Fermi smearing. Finite-step and SCF errors remain. Analytic gradients have narrower research-preview domains (Section S10.12). [d] Standalone correction gradient and stress. Dispersion is not combined with a GDF or BIPOLE optimization at this release. [e] The Γ-point evidence is a cutoff ladder whose rungs are archived runs against a matched PySCF reference, not a scored comparison row. The validation database holds 1 periodic cross-code total-energy comparison scored as a pass at this cut (SI S8), RHF LiF/pob-TZVP-rev2 on a $2 \times 2 \times 2$ mesh on the bipolar route against PySCF with matched exchange conventions, agreeing to 0.25 mE_h; the Γ-point route reported here is not that comparison, so this status rests on run-level evidence that the database records but does not score.

S10.1 Molecular self-consistent field

Restricted (RHF, RKS), unrestricted (UHF, UKS), and restricted open-shell (ROHF, ROKS) references are dispatched from a common SCF driver solving the Roothaan–Hall [23], Pople–Nesbet [24], and Roothaan open-shell equations [25, 26]. In the AO basis the closed-shell Fock matrix is

$$F_{\mu\nu} = h_{\mu\nu} + \sum_{\lambda\sigma} D_{\lambda\sigma} \left[(\mu\nu|\lambda\sigma) - \tfrac{1}{2}(\mu\sigma|\lambda\nu) \right], \tag{S1}$$

with the two-electron repulsion integrals (ERIs) evaluated through LIBINT. For KS-DFT the exact-exchange term is scaled by the hybrid fraction and the exchange–correlation potential is added (Section S10.2). The two-electron build is selected from a polymorphic family: a direct four-index build with Schwarz screening [27, 28], a resolution-of-the-identity build for the Coulomb and exchange matrices (RI-J and RI-JK) [29–32], and the chain-of-spheres RIJCOSX build [33, 34] for exact exchange on larger systems, with four grid tiers (GRID-X) and an automatic selection. Auxiliary basis sets are the standard fitting sets [35–37], with an automatic auxiliary basis available [38].

The initial-guess option defaults to AUTO, whose choice depends on the reference and system. Available guesses include the superposition of atomic densities (SAD) [39], the superposition of atomic potentials (SAP) [40, 41], the core Hamiltonian, an extended Hückel guess [42, 43], a projected minimal-basis guess (MINAO), a density restart, broken-symmetry spin-flip guesses, and a fragment molecular-orbital guess (FRAGMO) that assembles a block-diagonal density from converged fragments. Convergence is accelerated by Pulay's direct inversion in the iterative subspace (DIIS) [44, 45] on the commutator residual $\boldsymbol{e} = \boldsymbol{FDS} - \boldsymbol{SDF}$, with the energy-DIIS (EDIIS) [46], augmented Roothaan–Hall (ADIIS) [47], and KDIIS [48] variants, Saunders–Hillier level shifting [49], static and dynamic damping [50], and Fock-matrix mixing. Three second-order finishers are available for the asymptotic regime: a full-Hessian quadratically convergent step [51], Neese's approximate second-order SCF (SOSCF) [52], and a trust-region augmented-Hessian (TRAH) solver [53]. The trust-region form is designed to avoid saddle points from a poor start. A reusable library for exactly this task has recently appeared [54], and is discussed as an alternative in main-text Section 6.2. A post-SCF stability analysis [55] is available for UHF and UKS. Near-linear dependence of the AO basis is removed by canonical orthogonalization [56] below a configurable overlap eigenvalue threshold, with a pivoted Cholesky alternative [57]. Lehtola, Blockhuys, and Van Alsenoy review these ingredients [58].

The conventional restricted open-shell ground-state KS ansatz uses shared spatial orbitals for doubly occupied $\alpha\beta$ pairs and separate singly occupied orbitals for unpaired electrons. Pople, Gill, and Handy identified its inability to represent negative spin densities [59]. This is a limitation of that ansatz, rather than a blanket exclusion of useful restricted open-shell approximations. The optional ROKS reference offers a controlled spin-restricted alternative; `run_job` selects UKS by default for open shells, and UKS is recommended when spin polarization is needed.

The term ROKS also covers modern low-spin excited-state methods, implemented in established codes such as CP2K and Q-Chem. These commonly use a spin-adapted two-determinant energy expression and have practical applications to excitation energies and excited-state dynamics [60, 61]. They must be distinguished from the conventional restricted ground-state reference used here. Orbital-rotation instabilities can drive unconstrained excited-state optimization toward spuriously low energies; successful use

depends on the state-targeting and orbital optimization procedure [61]. The present validation does not establish a two-determinant excited-state ROKS implementation.

S10.2 Exchange–correlation functionals

All functionals are evaluated through LIBXC [12] on atom-centered Becke fuzzy-Voronoi grids [62] with Treutler–Ahlrichs or Mura–Knowles radial [63, 64] and Lebedev angular [65] quadrature. The LDA [66, 67], GGA (PBE [8], PBEsol [68], BLYP [69, 70], PW91 [71]), τ-dependent meta-GGA (TPSS [72], M06-L [73], SCAN [74], r^2SCAN [75], and its constraint-restored variant r^2SCAN01 [76]), global-hybrid (B3LYP [77, 78], PBE0 [7], PW1PW [79], M06 [80]), and range-separated hybrid families (CAM-B3LYP [81], HSE06 [82, 83], ωB97X [84]) are supported, together with the VV10 nonlocal correlation kernel [85], which completes ωB97X-V [86] and the meta-GGA hybrid ωB97M-V [87]. Any other LIBXC functional in these families, and user-defined weighted sums, are accepted through the same resolver. Laplacian-dependent meta-GGAs are the one LIBXC family not supported: a functional that requires $\nabla^2\rho$ is rejected at construction rather than evaluated with a missing ingredient. Analytic gradients cover the LDA, GGA, global hybrid, and τ-meta-GGA rungs (Table S25).

The B3LYP keyword follows a convention that must be stated, because the literature carries two functionals under that name [88, 89]. The original three-parameter fit of Stephens and co-workers [78] used the VWN local correlation functional fitted to the random-phase-approximation data (the "VWN(III)" or VWN-RPA form), and that is what GAUSSIAN, LIBXC (`HYB_GGA_XC_B3LYP`), PYSCF, and PSI4 mean by B3LYP. ORCA, TURBOMOLE, CRYSTAL, and CP2K instead pair the same mixing with the VWN5 form fitted to the Ceperley–Alder data (LIBXC `HYB_GGA_XC_B3LYP5`). VIBE-QC follows the second group: the bare `b3lyp` keyword selects the VWN5 form, the explicit spellings `b3lyp5` and `b3lypg` select each flavor unambiguously, and every output names the LIBXC identifier that was evaluated. The difference between the two flavors is about 10 mE_h per heavy atom in the total energy, so any cross-code comparison of a B3LYP total energy must match the flavor first. The runtime citation manifest cites the paper of Hertwig and Koch for every B3LYP calculation for that reason.

S10.3 Nuclear gradients and Hessians

Analytic nuclear gradients [90, 91] are implemented for HF and KS-DFT across the LDA, GGA, global-hybrid, and τ-meta-GGA rungs. For a closed-shell reference,

$$\frac{\partial E}{\partial \boldsymbol{R}_A} = \sum_{\mu\nu} D_{\mu\nu}\frac{\partial h_{\mu\nu}}{\partial \boldsymbol{R}_A} + \frac{1}{2}\sum_{\mu\nu\lambda\sigma} D_{\mu\nu}D_{\lambda\sigma}\frac{\partial(\mu\nu\|\lambda\sigma)}{\partial \boldsymbol{R}_A} - \sum_{\mu\nu} W_{\mu\nu}\frac{\partial S_{\mu\nu}}{\partial \boldsymbol{R}_A} + \frac{\partial E_\mathrm{nuc}}{\partial \boldsymbol{R}_A}, \tag{S2}$$

with W the energy-weighted density and the ∂S term the Pulay force. Integral derivatives come from LIBINT and ECP derivatives from LIBECPINT. Analytic gradients are also available for ROHF and for a restricted native CASSCF path [92, 93]: singlet, one root, and no selection of the numerical-gradient reference. The native IC-CASPT2 analytic path [94] additionally requires an exact-CI reference, zero IPEA and imaginary shifts, and explicit small-space eligibility. These source-supported conditions do not establish gradient accuracy for all admitted calculations. Analytic gradients for range-separated hybrids and VV10 are not implemented: the long-range exchange derivative and the VV10 nonlocal gradient are missing, the public first-gradient wrappers refuse these functionals by default, and geometry

optimization with them runs on central finite differences of the total energy (Table S25). Derivative support depends on the optimization backend. Native CASCI and the ASE CASCI/CASSCF path use finite differences of the wavefunction energy. Native MRPT has its own energy and guarded gradient paths; ASE NEVPT2/CASPT2/FCI requests instead fall back to the mean-field calculator. Public MP2, CCSD/(T), and DLPNO optimization and frequency calls likewise use the resolved mean-field reference before adding correlation. They cannot be described as derivatives of the correlated total energy. Method-specific derivative checks must preserve the actual backend, reference, active space, and options.

The gradient is passed through the ASE calculator interface [95] for geometry optimization, which offers rational-function (RFO/P-RFO) [96, 97], GDIIS [98], and FIRE [99] steppers in delocalized internal coordinates [100, 101], alongside the native L-BFGS and Brent line-search optimizers.

The low-level frequency driver accepts RHF, UHF, ROHF, RKS, and UKS; a ROKS Hessian request is refused. The reported M14 frequencies use central finite differences of the analytic RHF gradient. Analytic and numerical differentiation must also be distinguished within a response solver. At the numerical baseline, the ASE UKS Hessian uses a finite-difference routine. Other routines named analytic contain numerical response steps, and the RKS response implementation does not establish complete GGA response. This paper therefore does not claim general analytic HF/KS Hessian coverage. Harmonic analysis follows the Wilson formulation [102]; the actual derivative path remains part of each calculation's provenance.

S10.4 Møller–Plesset theory and double hybrids

MP2 [103] is implemented in canonical and resolution-of-the-identity (RI-MP2) [104] forms for closed-shell and unrestricted references. Semicanonical ROHF-MP2 [105] is RI only and retains an unscaled singles term. The methods also include the spin-component-scaled (SCS-MP2) [106] and scaled-opposite-spin (SOS-MP2) [107] variants. The double hybrids B2PLYP [108], DSD-PBEP86 [109], PWPB95 [110], and revDSD-PBEP86-D4 [111] add a perturbative RI-MP2 correlation correction to a hybrid KS reference. The canonical and RI kernels are validated on small molecules. Their memory envelope at production scale has not been characterized, and no production-scale claim is made in this release. Native MP2 defaults to conventional integrals, whereas the standalone SCS/SOS wrappers default to density fitting; the API and options must therefore accompany each result.

Frozen-core selection uses an element-based occupied-orbital count, an explicit count, or zero to correlate all explicitly represented electrons. ECP-replaced cores are subtracted from the default when reference provenance is available. This count policy does not reproduce a reference program's orbital-character ordering. The CAS inactive partition and CASPT2 inactive orbitals excluded from correlation are separate conventions; the top-level frozen-core option is refused for CAS routes. Accepted comparisons must identify the frozen orbitals, ECP electron counts, active space, and method-specific auxiliary basis.

S10.5 Coupled cluster: canonical, local, and frozen-natural-orbital

The coupled-cluster methods [112] available are CCSD [113] and its perturbative-triples extension CCSD(T) [114, 115], with RHF, UHF, and ROHF references, density-fitted by default [116] and on exact four-index integrals by option. The local-correlation implementation uses the domain-based local pair-natural-orbital hierarchy [117–120] DLPNO-MP2 [121], DLPNO-CCSD, and DLPNO-CCSD(T).

Closed-shell local CCSD defaults to a semicanonical compatibility triples mode named `t1`; open-shell local CCSD defaults to `t0`. These defaults must not be conflated with the iterative triples algorithm [122]; the distinct `t1-iterative` mode is opt-in. Local engines and explicitly selected dense pilots have different size guards, none of which establish practical production-size coverage. Open-shell DLPNO-MP2 follows Saitow and co-workers [123], and open-shell DLPNO-CCSD(T) remains a correctness pilot. These local-correlation routes are included as pilot implementations, and the validation tables do not claim Orca TightPNO parity for them. Frozen natural orbitals [116, 124] accelerate singlet RHF density-fitted CCSD(T) with a ΔMP2 correction. Its no-truncation check requires the same density-fitted Hamiltonian; the difference from exact four-index CCSD(T) is a separate comparison. Canonical CCSD(T) remains a dense small-molecule validation route. Production-size bounded-memory coverage is not claimed for canonical or density-fitted triples.

S10.6 Multireference and non-mean-field methods

The active-space calculations include CASCI and CASSCF [125]. They also include strongly contracted NEVPT2 [126] and a small-space CASPT2 diagnostic [127, 128]. The CASPT2 residual is retained as a limitation, not as established parity. CAS methods use common spatial orbitals from RHF, UHF, UNO, or ROHF, with UNO the open-shell default. This orbital choice does not establish an unrestricted correlated-orbital formalism. NEVPT2/CASPT2 default to a CASCI reference on HF-derived orbitals; explicit CASSCF options select a CASSCF reference. Reference, root, active orbitals, CASPT2 variant and shift conventions must therefore remain in the calculation record. CASCI has dense and direct-CI backends with different determinant limits; these admission guards are not validated memory bounds. Exact-limit CI agreement is defined at fixed orbitals. Independently optimized CASSCF calculations can reach different stationary orbital solutions, including for a single state; neither stationarity nor CI solver agreement establishes a global minimum. The exact determinant-based FCI solver [129] provides internal checks. Other active-space solvers are outside the evidence reported here.

S10.7 Excited states and electron propagators

Vertical excitation energies are obtained from linear-response time-dependent DFT [130, 131], in the full Casida form and in the Tamm–Dancoff approximation [132, 133], for restricted and unrestricted references, and from configuration-interaction singles [134]. The adiabatic kernel covers LDA, GGA, and global hybrids. Range-separated kernels are refused. The driver prints a UV/Vis spectrum and writes natural transition orbitals [135] to the qvf archive. The optional finite-difference excited-state gradient is restricted to a closed-shell RHF/TDA reference and labels its HF-CIS surface. Its coordinate-unit handling still requires verification in a calculation, and no excited-state derivative is used as validation evidence here. The excitation-energy calculation is reachable from `run_job` with `tddft=True` and from the `vibeqc tddft` command-line entry point. Ionization energies and electron affinities are computed from the one-particle Green's function through the second-order (GF2) and renormalized outer-valence approximations [136, 137].

S10.8 Dispersion and composite methods

London dispersion is treated by DFT-D3 with Becke–Johnson damping [9, 10] for molecular and periodic systems, optionally with the Axilrod–Teller–Muto three-body term [138], and by the charge-dependent D4 model [139, 140] for molecules. The reference `dftd4` program is the default D4 backend, and a native MPL-2.0 backend with electronegativity-equilibration charges covers H–Ne. The composite "-3c" methods HF-3c [141], PBEh-3c [142], B97-3c [143], HSE-3c [144], r^2SCAN-3c [145], and ωB97X-3c [146] combine a small-basis SCF with a geometrical counterpoise correction [147], a short-range basis-incompleteness term, and dispersion. The counterpoise and short-range terms reproduce the reference `mctc-gcp` implementation to better than 1 nE_h. The D3(BJ) energy enters the S22 interaction energies and the PBE0-D3(BJ) geometries of main-text Section 5 on both sides of the comparison. A component-level comparison of the molecular D3(BJ) term against the reference `dftd3` program is tracked in the issue tracker and is not claimed here.

S10.9 Implicit solvation and effective core potentials

Continuum solvation uses CPCM [148, 149] and COSMO [150] within the polarizable-continuum family [151], with the smooth continuous-surface-charge switching cavity of Scalmani and Frisch [152, 153] and, for CPCM, an analytic gradient at the API level (Table S25). ECP integrals are evaluated by LIBECPINT [154] for the Stuttgart–Cologne [155–157] and Hay–Wadt [158] families and enter the SCF and gradient paths, with automatic assignment for basis sets that carry an ECP by definition. ECPs are read from the Gaussian-94 and ORCA text formats as well as from the CRYSTAL input format. Selected three-dimensional GDF RHF, RKS, UHF, and UKS paths consume lattice-summed ECPs. BIPOLE and incompatible paths refuse ECP bases; no periodic ECP parity result is claimed. The CRYSTAL format matters here only because the pob basis families [5, 159] are distributed in it, and the Basis Set Exchange [160] converts between the formats in any case. No periodic-ECP validation is claimed (Table S26).

S10.10 Periodic SCF: crystal orbitals and $\boldsymbol{k}$ sampling

VIBE-QC implements the Bloch-summed Gaussian crystal-orbital ansatz [161] for HF and KS-DFT in one, two, and three dimensions. The periodic AO is the lattice sum

$$\phi_{\mu\boldsymbol{k}}(\boldsymbol{r}) = \sum_{\boldsymbol{R}} e^{i\boldsymbol{k}\cdot\boldsymbol{R}}\,\chi_\mu(\boldsymbol{r}-\boldsymbol{R}), \tag{S3}$$

and the Fock and density matrices carry a crystal-momentum label. The Brillouin zone is sampled on Monkhorst–Pack [162] or generalized regular [163] meshes, with the irreducible set generated by SPGLIB [164]. Mesh acceptance is route-specific: bulk GDF can expand an irreducible mesh to its full parent mesh, which does not itself reduce exchange work; the separate native irreducible-mesh exchange path excludes gradients. The slab GDF adapter accepts only a full, unshifted Γ-centered integer mesh $(n_1, n_2, 1)$, without custom weights. An automatic recommender selects the mesh density, optionally through a gated starter machine-learning predictor following the $\boldsymbol{k}$-spacing model of Choudhary and Tavazza [165]. Metallic occupations use Fermi–Dirac, Methfessel–Paxton [166], or Marzari–Vanderbilt cold smearing [167], or parameter-free Gilat–Raubenheimer tetrahedron integration [168], subject to backend restrictions. Positive-temperature Fermi–Dirac occupations use the Mermin free energy [169].

Two-dimensional systems use a vacuum-free slab Ewald summation [170, 171]. The shared infrastructure does not imply that every backend admits each mesh, occupation scheme, or derivative.

S10.11 GDF: periodic Gaussian density fitting

The GDF route fits the periodic density in an auxiliary Gaussian basis using the range-separated Gaussian-density-fitting construction [172]. The three-index integrals are fitted against the two-index lattice metric, Cholesky-decomposed, and contracted with the density to assemble the Hartree and exchange matrices. Admitted three-dimensional full-range-exchange calculations apply the Born–von-Kármán supercell Madelung correction once through the exchange matrix [173, 174]; this convention does not extend automatically to wires or slabs. A mixed-density-fitting variant [175] supplements the Gaussian fit with a plane-wave correction for heavy-core systems. Automatic routing selects GDF for ordinary three-dimensional closed shells and BIPOLE for UHF, UKS, and ROHF; two-dimensional AUTO uses the separate slab-Ewald route. Explicit GDF supports $\boldsymbol{\Gamma}$ and multi-$\boldsymbol{k}$ RHF, RKS, UHF, and UKS in bulk, and a closed-shell slab adapter. Accuracy depends on real-space domains, reciprocal fitting tails and meshes, and screening and quadrature tolerances. Section S4 separates the dominant real- and reciprocal-space errors in the retained comparisons.

Fixed-cell gradients cover selected Γ compcell/rsgdf paths, bulk multi-$\boldsymbol{k}$ rsgdf, and the separate closed-shell slab adapter. Bulk multi-$\boldsymbol{k}$ gradients require cached, fitted full-range exchange, uniform full-Brillouin-zone weights, and supported Aufbau or Fermi–Dirac occupations. Native irreducible-mesh exchange, DFT+U, range-separated or screened hybrids, and zero-temperature closed-shell band-overlap ensembles are excluded. The bulk multi-$\boldsymbol{k}$ KS force adds a numerical moving-grid XC correction at fixed accepted density (default central-difference half-step $1 \times 10^{-3}\, a_0$); its total is therefore not wholly analytic. MDF, legacy molecular-limit paths, and one-dimensional wires do not have public gradients in this scope.

S10.12 Bipolar-expansion Fock build

The second periodic route evaluates the two-electron lattice sum directly in real space under an exact Ewald split [176] of the Coulomb operator,

$$\frac{1}{r_{12}} = \frac{\mathrm{erfc}(\omega r_{12})}{r_{12}} + \frac{\mathrm{erf}(\omega r_{12})}{r_{12}}, \tag{S4}$$

whose split point ω leaves the energy invariant only in the limit of converged real- and reciprocal-space sums. SCF convergence alone does not establish this invariance. The short-range erfc channel of both the Coulomb and exchange builds is summed exactly over direct-lattice cells. The long-range erf channel is evaluated in reciprocal space together with the neutralizing background, so the reciprocal channel plays the role of the far-field model of the density. This reproduces the bielectronic-zone and monoelectronic-zone partition of the Crystal Coulomb machinery [161, 177–180] in the Ewald-split gauge. An optional restricted exact-ERI zone requires corrected exchange, a padded short-range domain, and a radius below the operator cutoff; it excludes pair-domain reduction and does not establish a negligible omitted tail. A quartet-level bipolar multipole far field is implemented but not certified: it does not preserve the three-translation periodic Fock domain of the exact contraction and is refused before the SCF.

For admitted three-dimensional full-range-exchange cases, GDF and BIPOLE can use matched corrected Ewald exchange conventions. Under Ewald Coulomb summation this is the BIPOLE default for ordinary complete meshes; an explicitly corrected request requires complete mesh metadata. Automatic selection for ad-hoc RHF lists can fall back to the legacy gauge, and uncorrected diagnostic paths remain reachable. Matching the convention permits comparison but does not establish equality at finite cutoffs. Route-to-route agreement is reported per case (Section S4). The bipolar-expansion route supports multi-$\boldsymbol{k}$ RHF, UHF, RKS, and UKS using direct lattice ERIs; symmetry reduction is route-dependent. Production atomic forces and fixed-cell relaxation use central differences of the converged total energy at $T = 0$, or Mermin free energy at positive Fermi smearing (default half-step $1 \times 10^{-3}\ a_0$). SCF and step convergence remain necessary. Analytic BIPOLE gradients are a research preview with restricted spin and domain combinations: exact-zone derivatives, fractional UHF, and fractional KS with padding are refused. No general fractional-occupation analytic-force accuracy is claimed here. Variable-cell optimization fails closed pending a certified strain convention. The route is deliberately narrow at its boundaries. Configurations whose Hamiltonian it cannot execute exactly, including effective-core-potential bases, periodic force constants, linear response, non-Fermi–Dirac occupation broadening, variable-cell and restart-preserving optimization, low-dimensional cells, and non-Monkhorst–Pack $\boldsymbol{k}$ sets under the corrected gauge, fail before the SCF starts rather than substituting a surrogate operator, and an unconverged SCF is a hard failure in both the manifest and the process exit code. It is the route benchmarked against Crystal 23 [181] and, at the Γ point, against PySCF in Section S4.

S10.13 Multi-$\boldsymbol{k}$ convergence aids and density mixing

At multiple $\boldsymbol{k}$ the DIIS residual is the Frobenius-norm-weighted concatenation of the per-$\boldsymbol{k}$ commutators. Beyond DIIS and EDIIS [46], the periodic driver offers reciprocal-space density mixing: Anderson/Pulay mixing [182], a limited-memory Broyden second-method mixer [183–185], and a Kerker preconditioner [186] for long-wavelength charge sloshing in metals. The mixers operate on the per-$\boldsymbol{k}$ density matrices and preserve the electron count exactly.

S10.14 Periodic Becke partition for XC grids

Real-space quadrature of the exchange–correlation energy in a Gaussian-basis periodic code goes back to the work of Towler, Zupan, and Causà [187]. For periodic KS-DFT vibe-qc extends the molecular Becke partition [62] by partitioning each home-cell grid point among the union of the home-cell atom and its periodic images within a cutoff. The weights sum to unity per grid point, and the partition-weighted density integrated over the home cell recovers the electron count per cell. This avoids the double counting of a naive scheme and the site-symmetry breaking that a molecular grid imposes on a high-symmetry cell. The periodic XC quadrature assembles the full cross-cell density at each grid point rather than the home-cell contribution alone.

S10.15 Space-group symmetry

spglib [164] identifies the space group. Admitted operations use AO permutations and real spherical-harmonic Wigner rotations checked against explicit rotations. Character tables project the AO basis into irreducible representations (SALCs) [188]. Cell-pair orbit reconstruction can reduce Fock and overlap

builds when the lattice domain and AO mapping permit; some BIPOLE paths require full direct builds. The same machinery supports geometry symmetrization, primitive-cell reduction, and irreducible-$\boldsymbol{k}$ generation.

S10.16 Population analysis, bonding analysis, and properties

Population analysis is provided in the Mulliken [189], Löwdin [190], and Hirshfeld [191] schemes, together with Mayer bond orders [192, 193], dipole moments, and the static polarizability, all computed from the converged density. Mulliken and Löwdin charges have no basis-set limit and are printed because users expect them, not because we recommend them. The code also computes intrinsic atomic orbitals (IAOs) [194]. QVF IAO analysis requires a converged closed-shell all-electron result and coverage by the Huzinaga MINI reference basis [195] with pure shells; ECPs and elements above $Z = 86$ are excluded. IAO partial charges are stored alongside IBO, Boys [196], and Pipek–Mezey [197] localized orbitals. This archival support does not establish basis stability for every admitted input. IAO charges are not included in the population text/JSON sidecars; those outputs are outside the IAO claim made here.

For the validated BIPOLE bulk cases, Mulliken and Löwdin charges and Mayer bond orders are evaluated from the same lattice density and overlap convention as the SCF energy. Ordinary electric dipole moments for three-dimensional bulk cells remain gated until a polarization convention is implemented. Periodic density volumes are evaluated from the density the SCF returned, with the Born–von-Kármán transform verified against every source block and against the primitive-cell electron count before export. A volume that cannot be produced truthfully is omitted rather than approximated.

Bonding analysis includes crystal-orbital overlap and Hamilton populations (COOP [198] and COHP [199, 200], including spin-polarized), Mayer bond orders for periodic systems, the electron localization function [201], the reduced density gradient for non-covalent-interaction analysis [202], and a quantum-theory-of-atoms-in-molecules topological analysis [203, 204] with critical-point search and bond-path tracing from the analytic density Hessian. For solids, VIBE-QC produces band structures along crystallographic $\boldsymbol{k}$ paths [205], fat (orbital-projected) bands, total and projected densities of states by tetrahedron or Gaussian broadening (with a Gilat–Raubenheimer variant for metals), and Fermi-surface exports.

References

[1] P. Jurečka, J. Šponer, J. Černý, and P. Hobza. "Benchmark database of accurate (MP2 and CCSD(T) complete basis set limit) interaction energies of small model complexes, DNA base pairs, and amino acid pairs". In: *Phys. Chem. Chem. Phys.* 8.17 (2006), pp. 1985–1993. DOI: 10.1039/B600027D.

[2] M. Véril, A. Scemama, M. Caffarel, F. Lipparini, M. Boggio-Pasqua, D. Jacquemin, and P.-F. Loos. "QUESTDB: A database of highly accurate excitation energies for the electronic structure community". In: *WIREs Comput. Mol. Sci.* 11.5 (2021), e1517. DOI: 10.1002/wcms.1517.

[3] V. K. Prasad, A. Otero-de-la-Roza, and G. A. DiLabio. “BH9, a New Comprehensive Benchmark Data Set for Barrier Heights and Reaction Energies: Assessment of Density Functional Approximations and Basis Set Incompleteness Potentials”. In: *J. Chem. Theory Comput.* 18.1 (2021), pp. 151–166. DOI: 10.1021/acs.jctc.1c00694.

[4] D. G. Liakos, Y. Guo, and F. Neese. “Comprehensive Benchmark Results for the Domain Based Local Pair Natural Orbital Coupled Cluster Method (DLPNO-CCSD(T)) for Closed- and Open-Shell Systems”. In: *J. Phys. Chem. A* 124.1 (2019), pp. 90–100. DOI: 10.1021/acs.jpca.9b05734.

[5] M. F. Peintinger, D. Vilela Oliveira, and T. Bredow. “Consistent Gaussian basis sets of triple-zeta valence with polarization quality for solid-state calculations”. In: *Journal of Computational Chemistry* 34.6 (2013), pp. 451–459. DOI: 10.1002/jcc.23153.

[6] F. Weigend and R. Ahlrichs. “Balanced basis sets of split valence, triple zeta valence and quadruple zeta valence quality for H to Rn: design and assessment of accuracy”. In: *Physical Chemistry Chemical Physics* 7.18 (2005), pp. 3297–3305. DOI: 10.1039/b508541a.

[7] C. Adamo and V. Barone. “Toward reliable density functional methods without adjustable parameters: the PBE0 model”. In: *The Journal of Chemical Physics* 110.13 (1999), pp. 6158–6170. DOI: 10.1063/1.478522.

[8] J. P. Perdew, K. Burke, and M. Ernzerhof. “Generalized gradient approximation made simple”. In: *Physical Review Letters* 77.18 (1996), pp. 3865–3868. DOI: 10.1103/PhysRevLett.77.3865.

[9] S. Grimme, J. Antony, S. Ehrlich, and H. Krieg. “A consistent and accurate ab initio parametrization of density functional dispersion correction (DFT-D) for the 94 elements H–Pu”. In: *The Journal of Chemical Physics* 132.15 (2010), p. 154104. DOI: 10.1063/1.3382344.

[10] S. Grimme, S. Ehrlich, and L. Goerigk. “Effect of the damping function in dispersion corrected density functional theory”. In: *Journal of Computational Chemistry* 32.7 (2011), pp. 1456–1465. DOI: 10.1002/jcc.21759.

[11] E. F. Valeev. *Libint: a library for the evaluation of molecular integrals of many-body operators over Gaussian functions*. https://github.com/evaleev/libint. Version 2.13.1. 2025.

[12] S. Lehtola, C. Steigemann, M. J. T. Oliveira, and M. A. L. Marques. “Recent developments in libxc — a comprehensive library of functionals for density functional theory”. In: *SoftwareX* 7 (2018), pp. 1–5. DOI: 10.1016/j.softx.2017.11.002.

[13] E. Posenitskiy, V. G. Chilkuri, A. Ammar, M. Hapka, K. Pernal, R. Shinde, E. J. Landinez Borda, C. Filippi, K. Nakano, O. Kohulák, S. Sorella, P. de Oliveira Castro, W. Jalby, P. L. Ríos, A. Alavi, and A. Scemama. “TREXIO: A file format and library for quantum chemistry”. In: *The Journal of Chemical Physics* 158.17 (2023), p. 174801. DOI: 10.1063/5.0148161.

[14] Anthropic. *Claude Opus 4.8*. https://www.anthropic.com/claude. Large language model. Used as a code-generation collaborator under human supervision. 2026.

[15] Anthropic. *Claude Opus 4.7*. https://www.anthropic.com/claude. Large language model. Used as a code-generation collaborator under human supervision. 2026.

[16] Anthropic. *Claude Opus 5*. https://www.anthropic.com/claude. Large language model. Used as a code-generation collaborator under human supervision. 2026.

[17] Anthropic. *Claude Fable 5*. https://www.anthropic.com/news/claude-fable-5-mythos-5. Large language model. Used as a code-generation collaborator under human supervision. 2026.

[18] DeepSeek. *DeepSeek Flash*. https://www.deepseek.com. Large language model used as a secondary code-generation and review aid. 2026.

[19] DeepSeek. *DeepSeek Pro*. https://www.deepseek.com. Large language model used as a secondary code-generation and review aid. 2026.

[20] DeepSeek. *DeepSeek v4 Pro*. https://www.deepseek.com. Large language model used as a secondary code-generation and review aid. 2026.

[21] OpenAI. *Codex 5.5*. https://openai.com. Code-generation model used as a secondary implementation aid. 2026.

[22] OpenAI. *ChatGPT 5.6 Sol*. https://openai.com. Large language model used as a secondary debugging and refactoring aid. 2026.

[23] C. C. J. Roothaan. "New developments in molecular orbital theory". In: *Reviews of Modern Physics* 23.2 (1951), pp. 69–89. DOI: 10.1103/RevModPhys.23.69.

[24] J. A. Pople and R. K. Nesbet. "Self-consistent orbitals for radicals". In: *The Journal of Chemical Physics* 22.3 (1954), pp. 571–572. DOI: 10.1063/1.1740120.

[25] C. C. J. Roothaan. "Self-consistent field theory for open shells of electronic systems". In: *Reviews of Modern Physics* 32.2 (1960), pp. 179–185. DOI: 10.1103/RevModPhys.32.179.

[26] M. F. Guest and V. R. Saunders. "On methods for converging open-shell Hartree-Fock wavefunctions". In: *Molecular Physics* 28.3 (1974), pp. 819–828. DOI: 10.1080/00268977400102171.

[27] J. Almlöf, K. J. Fægri, and K. Korsell. "Principles for a direct SCF approach to LCAO-MO ab-initio calculations". In: *Journal of Computational Chemistry* 3.4 (1982), pp. 385–399. DOI: 10.1002/jcc.540030314.

[28] M. Häser and R. Ahlrichs. "Improvements on the direct SCF method". In: *Journal of Computational Chemistry* 10.1 (1989), pp. 104–111. DOI: 10.1002/jcc.540100111.

[29] J. L. Whitten. "Coulombic potential energy integrals and approximations". In: *Journal of Chemical Physics* 58.10 (1973), pp. 4496–4501. DOI: 10.1063/1.1679012.

[30] B. I. Dunlap, J. W. D. Connolly, and J. R. Sabin. "On some approximations in applications of Xα theory". In: *Journal of Chemical Physics* 71.8 (1979), pp. 3396–3402. DOI: 10.1063/1.438728.

[31] K. Eichkorn, O. Treutler, H. Öhm, M. Häser, and R. Ahlrichs. "Auxiliary basis sets to approximate Coulomb potentials". In: *Chemical Physics Letters* 240.4 (1995), pp. 283–290. DOI: 10.1016/0009-2614(95)00621-A.

[32] F. Weigend. "Hartree-Fock exchange fitting basis sets for H to Rn". In: *Journal of Computational Chemistry* 29.2 (2008), pp. 167–175. DOI: 10.1002/jcc.20702.

[33] F. Neese, F. Wennmohs, A. Hansen, and U. Becker. "Efficient, approximate and parallel Hartree–Fock and hybrid DFT calculations. A "chain-of-spheres" algorithm for the Hartree–Fock exchange". In: *Chemical Physics* 356.1-3 (2009), pp. 98–109. DOI: 10.1016/j.chemphys.2008.10.036.

[34] R. Izsák and F. Neese. "An overlap fitted chain of spheres exchange method". In: *Journal of Chemical Physics* 135.14 (2011), p. 144105. DOI: 10.1063/1.3646921.

[35] F. Weigend, A. Köhn, and C. Hättig. "Efficient use of the correlation consistent basis sets in resolution of the identity MP2 calculations". In: *Journal of Chemical Physics* 116.8 (2002), pp. 3175–3183. DOI: 10.1063/1.1445115.

[36] F. Weigend. "A fully direct RI-HF algorithm: Implementation, optimised auxiliary basis sets, demonstration of accuracy and efficiency". In: *Physical Chemistry Chemical Physics* 4.18 (2002), pp. 4285–4291. DOI: 10.1039/B204199P.

[37] C. Hättig. "Optimization of auxiliary basis sets for RI-MP2 and RI-CC2 calculations: Core-valence and quintuple-ζ basis sets for H to Ar and QZVPP basis sets for Li to Kr". In: *Physical Chemistry Chemical Physics* 7.1 (2005), pp. 59–66. DOI: 10.1039/B415208E.

[38] G. L. Stoychev, A. A. Auer, and F. Neese. "Automatic Generation of Auxiliary Basis Sets". In: *Journal of Chemical Theory and Computation* 13.2 (2017), pp. 554–562. DOI: 10.1021/acs.jctc.6b01041.

[39] J. H. Van Lenthe, R. Zwaans, H. J. J. Van Dam, and M. F. Guest. "Starting SCF calculations by superposition of atomic densities". In: *Journal of Computational Chemistry* 27.8 (2006), pp. 926–932. DOI: 10.1002/jcc.20393.

[40] S. Lehtola. "Assessment of Initial Guesses for Self-Consistent Field Calculations. Superposition of Atomic Potentials: Simple yet Efficient". In: *Journal of Chemical Theory and Computation* 15.3 (2019), pp. 1593–1604. DOI: 10.1021/acs.jctc.8b01089.

[41] S. Lehtola, L. Visscher, and E. Engel. "Efficient implementation of the superposition of atomic potentials initial guess for electronic structure calculations in Gaussian basis sets". In: *Journal of Chemical Physics* 152.14 (2020), p. 144105. DOI: 10.1063/5.0004046.

[42] M. Wolfsberg and L. Helmholz. "The Spectra and Electronic Structure of the Tetrahedral Ions MnO4-, CrO4–, and ClO4-". In: *Journal of Chemical Physics* 20.5 (1952), pp. 837–843. DOI: 10.1063/1.1700580.

[43] R. Hoffmann. "An Extended Hückel Theory. I. Hydrocarbons". In: *Journal of Chemical Physics* 39.6 (1963), pp. 1397–1412. DOI: 10.1063/1.1734456.

[44] P. Pulay. "Convergence acceleration of iterative sequences. The case of SCF iteration". In: *Chemical Physics Letters* 73.2 (1980), pp. 393–398. DOI: 10.1016/0009-2614(80)80396-4.

[45] P. Pulay. "Improved SCF convergence acceleration". In: *Journal of Computational Chemistry* 3.4 (1982), pp. 556–560. DOI: 10.1002/jcc.540030413.

[46] K. N. Kudin, G. E. Scuseria, and E. Cancès. "A black-box self-consistent field convergence algorithm: one step closer". In: *The Journal of Chemical Physics* 116.19 (2002), pp. 8255–8261. DOI: 10.1063/1.1470195.

[47] X. Hu and W. Yang. "Accelerating self-consistent field convergence with the augmented Roothaan-Hall energy function". In: *Journal of Chemical Physics* 132.5 (2010), p. 054109. DOI: 10.1063/1.3304922.

[48] C. Kollmar. "Convergence optimization of restricted open-shell self-consistent field calculations". In: *International Journal of Quantum Chemistry* 62.6 (1997), pp. 617–637. DOI: 10.1002/(SICI)1097-461X(1997)62:6<617::AID-QUA5>3.0.CO;2-Z.

[49] V. R. Saunders and I. H. Hillier. "A "level-shifting" method for converging closed shell Hartree–Fock wave functions". In: *International Journal of Quantum Chemistry* 7.4 (1973), pp. 699–705. DOI: 10.1002/qua.560070407.

[50] M. C. Zerner and M. Hehenberger. “A dynamical damping scheme for converging molecular SCF calculations”. In: *Chemical Physics Letters* 62.3 (1979), pp. 550–554. DOI: 10.1016/0009-2614(79)80761-7.

[51] G. B. Bacskay. “A quadratically convergent Hartree-Fock (QC-SCF) method. Application to closed shell systems”. In: *Chemical Physics* 61.3 (1981), pp. 385–404. DOI: 10.1016/0301-0104(81)85156-7.

[52] F. Neese. “Approximate second-order SCF convergence for spin unrestricted wavefunctions”. In: *Chemical Physics Letters* 325.1–3 (2000), pp. 93–98. DOI: 10.1016/S0009-2614(00)00662-X.

[53] B. Helmich-Paris. “A trust-region augmented Hessian implementation for restricted and unrestricted Hartree-Fock and Kohn-Sham methods”. In: *Journal of Chemical Physics* 154.16 (2021), p. 164104. DOI: 10.1063/5.0040798.

[54] J. Greiner, I.-M. Høyvik, S. Lehtola, and J. J. Eriksen. “A reusable library for second-order orbital optimization using the trust region method”. In: *Journal of Chemical Theory and Computation* 22 (2026), pp. 881–895. DOI: 10.1021/acs.jctc.5c01576.

[55] R. Seeger and J. A. Pople. “Self-consistent molecular orbital methods. XVIII. Constraints and stability in Hartree-Fock theory”. In: *Journal of Chemical Physics* 66.7 (1977), pp. 3045–3050. DOI: 10.1063/1.434318.

[56] P.-O. Löwdin. “On the Nonorthogonality Problem”. In: *Advances in Quantum Chemistry* 5 (1970), pp. 185–199. DOI: 10.1016/S0065-3276(08)60339-1.

[57] S. Lehtola. “Curing basis set overcompleteness with pivoted Cholesky decompositions”. In: *Journal of Chemical Physics* 151.24 (2019), p. 241102. DOI: 10.1063/1.5139948.

[58] S. Lehtola, F. Blockhuys, and C. Van Alsenoy. “An Overview of Self-Consistent Field Calculations Within Finite Basis Sets”. In: *Molecules* 25.5 (2020), p. 1218. DOI: 10.3390/molecules25051218.

[59] J. A. Pople, P. M. W. Gill, and N. C. Handy. “Spin-unrestricted character of Kohn–Sham orbitals for open-shell systems”. In: *International Journal of Quantum Chemistry* 56.4 (1995), pp. 303–305. DOI: 10.1002/qua.560560414.

[60] I. Frank, J. Hutter, D. Marx, and M. Parrinello. “Molecular dynamics in low-spin excited states”. In: *The Journal of Chemical Physics* 108.10 (1998), pp. 4060–4069. DOI: 10.1063/1.475804.

[61] R. Büchel, L. Álvarez, J. Grage, D. Maniscalco, and I. Frank. “On the Simulation of Photoreactions Using Restricted Open-Shell Kohn–Sham Theory”. In: *Molecules* 29.18 (2024), p. 4509. DOI: 10.3390/molecules29184509.

[62] A. D. Becke. “A multicenter numerical integration scheme for polyatomic molecules”. In: *The Journal of Chemical Physics* 88.4 (1988), pp. 2547–2553. DOI: 10.1063/1.454033.

[63] O. Treutler and R. Ahlrichs. “Efficient molecular numerical integration schemes”. In: *The Journal of Chemical Physics* 102.1 (1995), pp. 346–354. DOI: 10.1063/1.469408.

[64] M. E. Mura and P. J. Knowles. “Improved radial grids for quadrature in molecular density-functional calculations”. In: *Journal of Chemical Physics* 104.24 (1996), pp. 9848–9858. DOI: 10.1063/1.471749.

[65] V. I. Lebedev. “Quadratures on a sphere”. In: *USSR Computational Mathematics and Mathematical Physics* 16.2 (1976), pp. 10–24. DOI: 10.1016/0041-5553(76)90100-2.

[66] J. C. Slater. "A Simplification of the Hartree-Fock Method". In: *Physical Review* 81.3 (1951), pp. 385–390. DOI: 10.1103/PhysRev.81.385.

[67] S. H. Vosko, L. Wilk, and M. Nusair. "Accurate spin-dependent electron liquid correlation energies for local spin density calculations: a critical analysis". In: *Canadian Journal of Physics* 58.8 (1980), pp. 1200–1211. DOI: 10.1139/p80-159.

[68] J. P. Perdew, A. Ruzsinszky, G. I. Csonka, O. A. Vydrov, G. E. Scuseria, L. A. Constantin, X. Zhou, and K. Burke. "Restoring the Density-Gradient Expansion for Exchange in Solids and Surfaces". In: *Physical Review Letters* 100.13 (2008), p. 136406. DOI: 10.1103/PhysRevLett.100.136406.

[69] A. D. Becke. "Density-functional exchange-energy approximation with correct asymptotic behavior". In: *Physical Review A* 38.6 (1988), pp. 3098–3100. DOI: 10.1103/PhysRevA.38.3098.

[70] C. Lee, W. Yang, and R. G. Parr. "Development of the Colle-Salvetti correlation-energy formula into a functional of the electron density". In: *Physical Review B* 37.2 (1988), pp. 785–789. DOI: 10.1103/PhysRevB.37.785.

[71] J. P. Perdew, J. A. Chevary, S. H. Vosko, K. A. Jackson, M. R. Pederson, D. J. Singh, and C. Fiolhais. "Atoms, molecules, solids, and surfaces: Applications of the generalized gradient approximation for exchange and correlation". In: *Physical Review B* 46.11 (1992), pp. 6671–6687. DOI: 10.1103/PhysRevB.46.6671.

[72] J. Tao, J. P. Perdew, V. N. Staroverov, and G. E. Scuseria. "Climbing the Density Functional Ladder: Nonempirical Meta-Generalized Gradient Approximation Designed for Molecules and Solids". In: *Physical Review Letters* 91.14 (2003), p. 146401. DOI: 10.1103/PhysRevLett.91.146401.

[73] Y. Zhao and D. G. Truhlar. "A new local density functional for main-group thermochemistry, transition metal bonding, thermochemical kinetics, and noncovalent interactions". In: *Journal of Chemical Physics* 125.19 (2006), p. 194101. DOI: 10.1063/1.2370993.

[74] J. Sun, A. Ruzsinszky, and J. P. Perdew. "Strongly Constrained and Appropriately Normed Semilocal Density Functional". In: *Physical Review Letters* 115.3 (2015), p. 036402. DOI: 10.1103/PhysRevLett.115.036402.

[75] J. W. Furness, A. D. Kaplan, J. Ning, J. P. Perdew, and J. Sun. "Accurate and numerically efficient r^2SCAN meta-generalized gradient approximation". In: *The Journal of Physical Chemistry Letters* 11.19 (2020), pp. 8208–8215. DOI: 10.1021/acs.jpclett.0c02405.

[76] J. W. Furness, A. D. Kaplan, J. Ning, J. P. Perdew, and J. Sun. "Construction of meta-GGA functionals through restoration of exact constraint adherence to regularized SCAN functionals". In: *The Journal of Chemical Physics* 156.3 (2022), p. 034109. DOI: 10.1063/5.0073623.

[77] A. D. Becke. "Density-functional thermochemistry. III. The role of exact exchange". In: *The Journal of Chemical Physics* 98.7 (1993), pp. 5648–5652. DOI: 10.1063/1.464913.

[78] P. J. Stephens, F. J. Devlin, C. F. Chabalowski, and M. J. Frisch. "Ab initio calculation of vibrational absorption and circular dichroism spectra using density functional force fields". In: *The Journal of Physical Chemistry* 98.45 (1994), pp. 11623–11627. DOI: 10.1021/j100096a001.

[79] T. Bredow and A. R. Gerson. "Effect of exchange and correlation on bulk properties of MgO, NiO, and CoO". In: *Physical Review B* 61.8 (2000), pp. 5194–5201. DOI: 10.1103/PhysRevB.61.5194.

[80] Y. Zhao and D. G. Truhlar. “The M06 suite of density functionals for main group thermochemistry, thermochemical kinetics, noncovalent interactions, excited states, and transition elements: two new functionals and systematic testing of four M06-class functionals and 12 other functionals”. In: *Theoretical Chemistry Accounts* 120.1–3 (2008), pp. 215–241. DOI: 10.1007/s00214-007-0310-x.

[81] T. Yanai, D. P. Tew, and N. C. Handy. “A new hybrid exchange-correlation functional using the Coulomb-attenuating method (CAM-B3LYP)”. In: *Chemical Physics Letters* 393.1–3 (2004), pp. 51–57. DOI: 10.1016/j.cplett.2004.06.011.

[82] J. Heyd, G. E. Scuseria, and M. Ernzerhof. “Hybrid functionals based on a screened Coulomb potential”. In: *Journal of Chemical Physics* 118.18 (2003), pp. 8207–8215. DOI: 10.1063/1.1564060.

[83] A. V. Krukau, O. A. Vydrov, A. F. Izmaylov, and G. E. Scuseria. “Influence of the exchange screening parameter on the performance of screened hybrid functionals”. In: *Journal of Chemical Physics* 125.22 (2006), p. 224106. DOI: 10.1063/1.2404663.

[84] J.-D. Chai and M. Head-Gordon. “Systematic optimization of long-range corrected hybrid density functionals”. In: *The Journal of Chemical Physics* 128.8 (2008), p. 084106. DOI: 10.1063/1.2834918.

[85] O. A. Vydrov and T. Van Voorhis. “Nonlocal van der Waals density functional: the simpler the better”. In: *The Journal of Chemical Physics* 133.24 (2010), p. 244103. DOI: 10.1063/1.3521275.

[86] N. Mardirossian and M. Head-Gordon. “ωB97X-V: A 10-parameter, range-separated hybrid, generalized gradient approximation density functional with nonlocal correlation, designed by a survival-of-the-fittest strategy”. In: *Physical Chemistry Chemical Physics* 16.21 (2014), pp. 9904–9924. DOI: 10.1039/C3CP54374A.

[87] N. Mardirossian and M. Head-Gordon. “ωB97M-V: a combinatorially optimized, range-separated hybrid, meta-GGA density functional with VV10 nonlocal correlation”. In: *The Journal of Chemical Physics* 144.21 (2016), p. 214110. DOI: 10.1063/1.4952647.

[88] R. H. Hertwig and W. Koch. “On the parameterization of the local correlation functional. What is B3LYP?” In: *Chemical Physics Letters* 268.5–6 (1997), pp. 345–351. DOI: 10.1016/S0009-2614(97)00207-8.

[89] S. Lehtola and M. A. L. Marques. “Reproducibility of density functional approximations: How new functionals should be reported”. In: *The Journal of Chemical Physics* 159.11 (2023), p. 114116. DOI: 10.1063/5.0167763.

[90] P. Pulay. “Ab initio calculation of force constants and equilibrium geometries in polyatomic molecules. I. Theory”. In: *Molecular Physics* 17.2 (1969), pp. 197–204. DOI: 10.1080/00268976900100941.

[91] J. A. Pople, R. Krishnan, H. B. Schlegel, and J. S. Binkley. “Derivative studies in Hartree–Fock and Møller–Plesset theories”. In: *International Journal of Quantum Chemistry* 16.S13 (1979), pp. 225–241. DOI: 10.1002/qua.560160825.

[92] N. C. Handy and H. F. I. Schaefer. “On the evaluation of analytic energy derivatives for correlated wave functions”. In: *Journal of Chemical Physics* 81.11 (1984), pp. 5031–5033. DOI: 10.1063/1.447489.

[93] J. Stålring, A. Bernhardsson, and R. Lindh. “Analytical gradients of a state average MCSCF state and a state average diagnostic”. In: *Molecular Physics* 99.2 (2001), pp. 103–114. doi: 10.1080/002689700110005642.

[94] P. Celani and H.-J. Werner. “Analytical energy gradients for internally contracted second-order multireference perturbation theory”. In: *Journal of Chemical Physics* 119.10 (2003), pp. 5044–5057. doi: 10.1063/1.1597672.

[95] A. Hjorth Larsen, J. Jørgen Mortensen, J. Blomqvist, I. E. Castelli, R. Christensen, M. Dułak, J. Friis, M. N. Groves, B. Hammer, C. Hargus, E. D. Hermes, P. C. Jennings, P. Bjerre Jensen, J. Kermode, J. R. Kitchin, E. Leonhard Kolsbjerg, J. Kubal, K. Kaasbjerg, S. Lysgaard, J. Bergmann Maronsson, T. Maxson, T. Olsen, L. Pastewka, A. Peterson, C. Rostgaard, J. Schiøtz, O. Schütt, M. Strange, K. S. Thygesen, T. Vegge, L. Vilhelmsen, M. Walter, Z. Zeng, and K. W. Jacobsen. “The atomic simulation environment — a Python library for working with atoms”. In: *Journal of Physics: Condensed Matter* 29.27 (2017), p. 273002. doi: 10.1088/1361-648X/aa680e.

[96] A. Banerjee, N. Adams, J. Simons, and R. Shepard. “Search for stationary points on surfaces”. In: *The Journal of Physical Chemistry* 89.1 (1985), pp. 52–57. doi: 10.1021/j100247a015.

[97] J. Baker. “An algorithm for the location of transition states”. In: *Journal of Computational Chemistry* 7.4 (1986), pp. 385–395. doi: 10.1002/jcc.540070402.

[98] P. Császár and P. Pulay. “Geometry optimization by direct inversion in the iterative subspace”. In: *Journal of Molecular Structure* 114 (1984), pp. 31–34. doi: 10.1016/S0022-2860(84)87198-7.

[99] E. Bitzek, P. Koskinen, F. Gähler, M. Moseler, and P. Gumbsch. “Structural relaxation made simple”. In: *Physical Review Letters* 97.17 (2006), p. 170201. doi: 10.1103/PhysRevLett.97.170201.

[100] J. Baker, A. Kessi, and B. Delley. “The generation and use of delocalized internal coordinates in geometry optimization”. In: *The Journal of Chemical Physics* 105.1 (1996), pp. 192–212. doi: 10.1063/1.471864.

[101] C. Peng, P. Y. Ayala, H. B. Schlegel, and M. J. Frisch. “Using redundant internal coordinates to optimize equilibrium geometries and transition states”. In: *Journal of Computational Chemistry* 17.1 (1996), pp. 49–56. doi: 10.1002/(SICI)1096-987X(19960115)17:1<49::AID-JCC5>3.0.CO;2-0.

[102] E. B. Wilson, J. C. Decius, and P. C. Cross. *Molecular Vibrations: The Theory of Infrared and Raman Vibrational Spectra*. McGraw-Hill, 1955.

[103] C. Møller and M. S. Plesset. “Note on an approximation treatment for many-electron systems”. In: *Physical Review* 46.7 (1934), pp. 618–622. doi: 10.1103/PhysRev.46.618.

[104] M. Feyereisen, G. Fitzgerald, and A. Komornicki. “Use of approximate integrals in ab initio theory. An application in MP2 energy calculations”. In: *Chemical Physics Letters* 208.5 (1993), pp. 359–363. doi: 10.1016/0009-2614(93)87156-W.

[105] P. J. Knowles, J. S. Andrews, R. D. Amos, N. C. Handy, and J. A. Pople. “Restricted Møller-Plesset theory for open-shell molecules”. In: *Chemical Physics Letters* 186.2 (1991), pp. 130–136. doi: 10.1016/S0009-2614(91)85118-G.

[106] S. Grimme. “Improved second-order Møller–Plesset perturbation theory by separate scaling of parallel- and antiparallel-spin pair correlation energies”. In: *The Journal of Chemical Physics* 118.20 (2003), pp. 9095–9102. DOI: 10.1063/1.1569242.

[107] Y. Jung, R. C. Lochan, A. D. Dutoi, and M. Head-Gordon. “Scaled opposite-spin second order Møller–Plesset correlation energy: an economical electronic structure method”. In: *The Journal of Chemical Physics* 121.20 (2004), pp. 9793–9802. DOI: 10.1063/1.1809602.

[108] S. Grimme. “Semiempirical hybrid density functional with perturbative second-order correlation”. In: *The Journal of Chemical Physics* 124.3 (2006), p. 034108. DOI: 10.1063/1.2148954.

[109] S. Kozuch and J. M. L. Martin. “DSD-PBEP86: in search of the best double-hybrid DFT with spin-component scaled MP2 and dispersion corrections”. In: *Physical Chemistry Chemical Physics* 13.45 (2011), pp. 20104–20107. DOI: 10.1039/C1CP22592H.

[110] L. Goerigk and S. Grimme. “Efficient and Accurate Double-Hybrid-Meta-GGA Density Functionals - Evaluation with the Extended GMTKN30 Database for General Main Group Thermochemistry, Kinetics, and Noncovalent Interactions”. In: *Journal of Chemical Theory and Computation* 7.2 (2011), pp. 291–309. DOI: 10.1021/ct100466k.

[111] G. Santra, N. Sylvetsky, and J. M. L. Martin. “Minimally empirical double-hybrid functionals trained against the GMTKN55 database: revDSD-PBEP86-D4, revDOD-PBE-D4, and DOD-SCAN-D4”. In: *The Journal of Physical Chemistry A* 123.24 (2019), pp. 5129–5143. DOI: 10.1021/acs.jpca.9b03157.

[112] J. Čížek. “On the Correlation Problem in Atomic and Molecular Systems. Calculation of Wavefunction Components in Ursell-Type Expansion Using Quantum-Field Theoretical Methods”. In: *Journal of Chemical Physics* 45.11 (1966), pp. 4256–4266. DOI: 10.1063/1.1727484.

[113] G. D. Purvis and R. J. Bartlett. “A full coupled-cluster singles and doubles model: the inclusion of disconnected triples”. In: *The Journal of Chemical Physics* 76.4 (1982), pp. 1910–1918. DOI: 10.1063/1.443164.

[114] M. Urban, J. Noga, S. J. Cole, and R. J. Bartlett. “Towards a full CCSDT model for electron correlation”. In: *Journal of Chemical Physics* 83.8 (1985), pp. 4041–4046. DOI: 10.1063/1.449067.

[115] K. Raghavachari, G. W. Trucks, J. A. Pople, and M. Head-Gordon. “A fifth-order perturbation comparison of electron correlation theories”. In: *Chemical Physics Letters* 157.6 (1989), pp. 479–483. DOI: 10.1016/S0009-2614(89)87395-6.

[116] A. E. DePrince and C. D. Sherrill. “Accuracy and efficiency of coupled-cluster theory using density fitting / Cholesky decomposition, frozen natural orbitals, and a t_1-transformed Hamiltonian”. In: *Journal of Chemical Theory and Computation* 9.6 (2013), pp. 2687–2696. DOI: 10.1021/ct400250u.

[117] F. Neese, A. Hansen, and D. G. Liakos. “Efficient and accurate approximations to the local coupled cluster singles doubles method using a truncated pair natural orbital basis”. In: *Journal of Chemical Physics* 131.6 (2009), p. 064103. DOI: 10.1063/1.3173827.

[118] C. Riplinger and F. Neese. “An efficient and near linear scaling pair natural orbital based local coupled cluster method”. In: *The Journal of Chemical Physics* 138.3 (2013), p. 034106. DOI: 10.1063/1.4773581.

[119] C. Riplinger, B. Sandhoefer, A. Hansen, and F. Neese. “Natural triple excitations in local coupled cluster calculations with pair natural orbitals”. In: *The Journal of Chemical Physics* 139.13 (2013), p. 134101. DOI: 10.1063/1.4821834.

[120] C. Riplinger, P. Pinski, U. Becker, E. F. Valeev, and F. Neese. “Sparse maps—A systematic infrastructure for reduced-scaling electronic structure methods. II. Linear scaling domain based pair natural orbital coupled cluster theory”. In: *Journal of Chemical Physics* 144.2 (2016), p. 024109. DOI: 10.1063/1.4939030.

[121] P. Pinski, C. Riplinger, E. F. Valeev, and F. Neese. “Sparse maps—A systematic infrastructure for reduced-scaling electronic structure methods. I. An efficient and simple linear scaling local MP2 method that uses an intermediate basis of pair natural orbitals”. In: *Journal of Chemical Physics* 143.3 (2015), p. 034108. DOI: 10.1063/1.4926879.

[122] Y. Guo, C. Riplinger, U. Becker, D. G. Liakos, Y. Minenkov, L. Cavallo, and F. Neese. “Communication: an improved linear scaling perturbative triples correction for the domain based local pair-natural orbital based singles and doubles coupled cluster method [DLPNO-CCSD(T)]”. In: *The Journal of Chemical Physics* 148.1 (2018), p. 011101. DOI: 10.1063/1.5011798.

[123] M. Saitow, U. Becker, C. Riplinger, E. F. Valeev, and F. Neese. “A new near-linear scaling, efficient and accurate, open-shell domain-based local pair natural orbital coupled cluster singles and doubles theory”. In: *The Journal of Chemical Physics* 146.16 (2017), p. 164105. DOI: 10.1063/1.4981521.

[124] A. G. Taube and R. J. Bartlett. “Frozen natural orbital coupled-cluster theory: Forces and application to decomposition of nitroethane”. In: *Journal of Chemical Physics* 128.16 (2008), p. 164101. DOI: 10.1063/1.2902285.

[125] B. O. Roos, P. R. Taylor, and P. E. M. Siegbahn. “A complete active space SCF method (CASSCF) using a density matrix formulated super-CI approach”. In: *Chemical Physics* 48.2 (1980), pp. 157–173. DOI: 10.1016/0301-0104(80)80045-0.

[126] C. Angeli, R. Cimiraglia, S. Evangelisti, T. Leininger, and J.-P. Malrieu. “Introduction of n-electron valence states for multireference perturbation theory”. In: *The Journal of Chemical Physics* 114.23 (2001), pp. 10252–10264. DOI: 10.1063/1.1361246.

[127] K. Andersson, P.-Å. Malmqvist, B. O. Roos, A. J. Sadlej, and K. Wolinski. “Second-order perturbation theory with a CASSCF reference function”. In: *Journal of Physical Chemistry* 94.14 (1990), pp. 5483–5488. DOI: 10.1021/j100377a012.

[128] K. Andersson, P.-Å. Malmqvist, and B. O. Roos. “Second-order perturbation theory with a complete active space self-consistent field reference function”. In: *The Journal of Chemical Physics* 96.2 (1992), pp. 1218–1226. DOI: 10.1063/1.462209.

[129] P. J. Knowles and N. C. Handy. “A new determinant-based full configuration interaction method”. In: *Chemical Physics Letters* 111.4-5 (1984), pp. 315–321. DOI: 10.1016/0009-2614(84)85513-X.

[130] E. Runge and E. K. U. Gross. “Density-Functional Theory for Time-Dependent Systems”. In: *Physical Review Letters* 52.12 (1984), pp. 997–1000. DOI: 10.1103/PhysRevLett.52.997.

[131] M. E. Casida. “Time-Dependent Density Functional Response Theory for Molecules”. In: *Recent Advances in Density Functional Methods, Part I (World Scientific)* (1995), pp. 155–192. DOI: 10.1142/9789812830586_0005.

[132] S. Hirata and M. Head-Gordon. “Time-dependent density functional theory within the Tamm-Dancoff approximation”. In: *Chemical Physics Letters* 314.3–4 (1999), pp. 291–299. DOI: 10.1016/S0009-2614(99)01149-5.

[133] R. Bauernschmitt and R. Ahlrichs. “Treatment of electronic excitations within the adiabatic approximation of time dependent density functional theory”. In: *Chemical Physics Letters* 256.4-5 (1996), pp. 454–464. DOI: 10.1016/0009-2614(96)00440-X.

[134] J. B. Foresman, M. Head-Gordon, J. A. Pople, and M. J. Frisch. “Toward a systematic molecular orbital theory for excited states”. In: *The Journal of Physical Chemistry* 96.1 (1992), pp. 135–149. DOI: 10.1021/j100180a030.

[135] R. L. Martin. “Natural transition orbitals”. In: *The Journal of Chemical Physics* 118.11 (2003), pp. 4775–4777. DOI: 10.1063/1.1558471.

[136] L. S. Cederbaum. “One-body Green’s function for atoms and molecules: theory and application”. In: *Journal of Physics B: Atomic and Molecular Physics* 8.2 (1975), pp. 290–303. DOI: 10.1088/0022-3700/8/2/018.

[137] W. von Niessen, J. Schirmer, and L. S. Cederbaum. “Computational methods for the one-particle Green’s function”. In: *Computer Physics Reports* 1.2 (1984), pp. 57–125. DOI: 10.1016/0167-7977(84)90002-9.

[138] B. M. Axilrod and E. Teller. “Interaction of the van der Waals Type Between Three Atoms”. In: *Journal of Chemical Physics* 11.6 (1943), pp. 299–300. DOI: 10.1063/1.1723844.

[139] E. Caldeweyher, C. Bannwarth, and S. Grimme. “Extension of the D3 dispersion coefficient model”. In: *Journal of Chemical Physics* 147.3 (2017), p. 034112. DOI: 10.1063/1.4993215.

[140] E. Caldeweyher, S. Ehlert, A. Hansen, H. Neugebauer, S. Spicher, C. Bannwarth, and S. Grimme. “A generally applicable atomic-charge dependent London dispersion correction”. In: *The Journal of Chemical Physics* 150.15 (2019), p. 154122. DOI: 10.1063/1.5090222.

[141] R. Sure and S. Grimme. “Corrected small basis set Hartree–Fock method for large systems”. In: *Journal of Computational Chemistry* 34.19 (2013), pp. 1672–1685. DOI: 10.1002/jcc.23317.

[142] S. Grimme, J. G. Brandenburg, C. Bannwarth, and A. Hansen. “Consistent structures and interactions by density functional theory with small atomic orbital basis sets”. In: *Journal of Chemical Physics* 143.5 (2015), p. 054107. DOI: 10.1063/1.4927476.

[143] J. G. Brandenburg, C. Bannwarth, A. Hansen, and S. Grimme. “B97-3c: A revised low-cost variant of the B97-D density functional method”. In: *Journal of Chemical Physics* 148.6 (2018), p. 064104. DOI: 10.1063/1.5012601.

[144] J. G. Brandenburg, E. Caldeweyher, and S. Grimme. “Screened exchange hybrid density functional for accurate and efficient structures and interaction energies”. In: *Physical Chemistry Chemical Physics* 18.23 (2016), pp. 15519–15523. DOI: 10.1039/c6cp01697a.

[145] S. Grimme, A. Hansen, S. Ehlert, and J.-M. Mewes. “r^{2}SCAN-3c: a “Swiss army knife” composite electronic-structure method”. In: *The Journal of Chemical Physics* 154.6 (2021), p. 064103. DOI: 10.1063/5.0040021.

[146] M. Müller, A. Hansen, and S. Grimme. “ωB97X-3c: A composite range-separated hybrid DFT method with a molecule-optimized polarized valence double-ζ basis set”. In: *Journal of Chemical Physics* 158.1 (2023), p. 014103. DOI: 10.1063/5.0133026.

[147] H. Kruse and S. Grimme. “A geometrical correction for the inter- and intra-molecular basis set superposition error in Hartree–Fock and density functional theory calculations for large systems”. In: *The Journal of Chemical Physics* 136.15 (2012), p. 154101. DOI: 10.1063/1.3700154.

[148] V. Barone and M. Cossi. “Quantum calculation of molecular energies and energy gradients in solution by a conductor solvent model”. In: *The Journal of Physical Chemistry A* 102.11 (1998), pp. 1995–2001. DOI: 10.1021/jp9716997.

[149] M. Cossi, N. Rega, G. Scalmani, and V. Barone. “Energies, structures, and electronic properties of molecules in solution with the C-PCM solvation model”. In: *Journal of Computational Chemistry* 24.6 (2003), pp. 669–681. DOI: 10.1002/jcc.10189.

[150] A. Klamt and G. Schüürmann. “COSMO: a new approach to dielectric screening in solvents with explicit expressions for the screening energy and its gradient”. In: *Journal of the Chemical Society, Perkin Transactions 2* 5 (1993), pp. 799–805. DOI: 10.1039/P29930000799.

[151] J. Tomasi, B. Mennucci, and R. Cammi. “Quantum mechanical continuum solvation models”. In: *Chemical Reviews* 105.8 (2005), pp. 2999–3094. DOI: 10.1021/cr9904009.

[152] G. Scalmani and M. J. Frisch. “Continuous surface charge polarizable continuum models of solvation. I. General formalism”. In: *J. Chem. Phys.* 132.11 (2010), p. 114110. DOI: 10.1063/1.3359469.

[153] A. W. Lange and J. M. Herbert. “A smooth, nonsingular, and faithful discretization scheme for polarizable continuum models: the switching/Gaussian approach”. In: *The Journal of Chemical Physics* 133.24 (2010), p. 244111. DOI: 10.1063/1.3511297.

[154] R. A. Shaw and J. G. Hill. “Prescreening and efficiency in the evaluation of integrals over ab initio effective core potentials”. In: *The Journal of Chemical Physics* 147.7 (2017), p. 074108. DOI: 10.1063/1.4986887.

[155] D. Andrae, U. Häußermann, M. Dolg, H. Stoll, and H. Preuß. “Energy-adjusted ab initio pseudopotentials for the second and third row transition elements”. In: *Theoretica Chimica Acta* 77.2 (1990), pp. 123–141. DOI: 10.1007/BF01114537.

[156] B. Metz, H. Stoll, and M. Dolg. “Small-core multiconfiguration-Dirac–Hartree–Fock-adjusted pseudopotentials for post-d main group elements: application to PbH and PbO”. In: *The Journal of Chemical Physics* 113.7 (2000), pp. 2563–2569. DOI: 10.1063/1.1305880.

[157] K. A. Peterson, D. Figgen, E. Goll, H. Stoll, and M. Dolg. “Systematically convergent basis sets with relativistic pseudopotentials. II. Small-core pseudopotentials and correlation consistent basis sets for the post-d group 16-18 elements”. In: *Journal of Chemical Physics* 119.21 (2003), pp. 11113–11123. DOI: 10.1063/1.1622924.

[158] P. J. Hay and W. R. Wadt. “Ab initio effective core potentials for molecular calculations. Potentials for the transition metal atoms Sc to Hg”. In: *Journal of Chemical Physics* 82.1 (1985), pp. 270–283. DOI: 10.1063/1.448799.

[159] D. Vilela Oliveira, J. Laun, M. F. Peintinger, and T. Bredow. “BSSE-correction scheme for consistent Gaussian basis sets of double- and triple-zeta valence with polarization quality for solid-state calculations”. In: *Journal of Computational Chemistry* 40.27 (2019), pp. 2364–2376. DOI: 10.1002/jcc.26013.

[160] B. P. Pritchard, D. Altarawy, B. Didier, T. D. Gibson, and T. L. Windus. "New Basis Set Exchange: An Open, Up-to-Date Resource for the Molecular Sciences Community". In: *Journal of Chemical Information and Modeling* 59.11 (2019), pp. 4814–4820. DOI: 10.1021/acs.jcim.9b00725.

[161] C. Pisani, R. Dovesi, and C. Roetti. *Hartree–Fock Ab Initio Treatment of Crystalline Systems*. Vol. 48. Lecture Notes in Chemistry. Berlin, Heidelberg: Springer, 1988. DOI: 10.1007/978-3-642-93385-1.

[162] H. J. Monkhorst and J. D. Pack. "Special points for Brillouin-zone integrations". In: *Physical Review B* 13.12 (1976), pp. 5188–5192. DOI: 10.1103/PhysRevB.13.5188.

[163] P. Wisesa, K. A. McGill, and T. Mueller. "Efficient generation of generalized Monkhorst-Pack grids through the use of informatics". In: *Physical Review B* 93.15 (2016), p. 155109. DOI: 10.1103/PhysRevB.93.155109.

[164] A. Togo and I. Tanaka. "Spglib: a software library for crystal symmetry search". In: *arXiv preprint* (2018). arXiv: 1808.01590.

[165] K. Choudhary and F. Tavazza. "Convergence and machine learning predictions of Monkhorst-Pack k-points and plane-wave cut-off in high-throughput DFT calculations". In: *Computational Materials Science* 161 (2019), pp. 300–308. DOI: 10.1016/j.commatsci.2019.02.006.

[166] M. Methfessel and A. T. Paxton. "High-precision sampling for Brillouin-zone integration in metals". In: *Physical Review B* 40.6 (1989), pp. 3616–3621. DOI: 10.1103/PhysRevB.40.3616.

[167] N. Marzari, D. Vanderbilt, A. De Vita, and M. C. Payne. "Thermal contraction and disordering of the Al(110) surface". In: *Physical Review Letters* 82.16 (1999), pp. 3296–3299. DOI: 10.1103/PhysRevLett.82.3296.

[168] G. Gilat and L. J. Raubenheimer. "Accurate Numerical Method for Calculating Frequency-Distribution Functions in Solids". In: *Physical Review* 144 (1966), pp. 390–395. DOI: 10.1103/PhysRev.144.390.

[169] N. D. Mermin. "Thermal Properties of the Inhomogeneous Electron Gas". In: *Physical Review* 137.5A (1965), A1441–A1443. DOI: 10.1103/PhysRev.137.A1441.

[170] D. E. Parry. "The electrostatic potential in the surface region of an ionic crystal". In: *Surface Science* 49 (1975), pp. 433–440. DOI: 10.1016/0039-6028(75)90362-3.

[171] S. W. de Leeuw and J. W. Perram. "Electrostatic lattice sums for semi-infinite lattices". In: *Molecular Physics* 37.4 (1979), pp. 1313–1322. DOI: 10.1080/00268977900100951.

[172] H.-Z. Ye and T. C. Berkelbach. "Fast periodic Gaussian density fitting by range separation". In: *J. Chem. Phys.* 154.13 (2021), p. 131104. DOI: 10.1063/5.0046617.

[173] F. Gygi and A. Baldereschi. "Self-consistent Hartree-Fock and screened-exchange calculations in solids: Application to silicon". In: *Physical Review B* 34 (1986), pp. 4405–4408. DOI: 10.1103/PhysRevB.34.4405.

[174] J. Spencer and A. Alavi. "Efficient calculation of the exact exchange energy in periodic systems using a truncated Coulomb potential". In: *Physical Review B* 77 (2008), p. 193110. DOI: 10.1103/PhysRevB.77.193110.

[175] Q. Sun, T. C. Berkelbach, J. D. McClain, and G. K.-L. Chan. "Gaussian and plane-wave mixed density fitting for periodic systems". In: *The Journal of Chemical Physics* 147.16 (2017), p. 164119. DOI: 10.1063/1.4998644.

[176] P. P. Ewald. “Die Berechnung optischer und elektrostatischer Gitterpotentiale”. In: *Annalen der Physik* 369.3 (1921), pp. 253–287. DOI: 10.1002/andp.19213690304.

[177] C. Pisani and R. Dovesi. “Exact-exchange Hartree-Fock calculations for periodic systems. I. Illustration of the method”. In: *International Journal of Quantum Chemistry* 17.3 (1980), pp. 501–516. DOI: 10.1002/qua.560170311.

[178] R. Dovesi, C. Pisani, C. Roetti, and V. R. Saunders. “Treatment of Coulomb interactions in Hartree-Fock calculations of periodic systems”. In: *Phys. Rev. B* 28.10 (1983), pp. 5781–5792. DOI: 10.1103/PhysRevB.28.5781.

[179] M. Causà, R. Dovesi, R. Orlando, C. Pisani, and V. R. Saunders. “Treatment of the exchange interactions in Hartree-Fock LCAO calculations of periodic systems”. In: *The Journal of Physical Chemistry* 92 (1988), pp. 909–913. DOI: 10.1021/j100315a010.

[180] V. R. Saunders, C. Freyria-Fava, R. Dovesi, L. Salasco, and C. Roetti. “On the electrostatic potential in crystalline systems where the charge density is expanded in Gaussian functions”. In: *Molecular Physics* 77 (1992), pp. 629–665. DOI: 10.1080/00268979200102671.

[181] A. Erba, J. K. Desmarais, S. Casassa, B. Civalleri, L. Donà, I. J. Bush, B. Searle, L. Maschio, L. Edith-Daga, A. Cossard, C. Ribaldone, E. Ascrizzi, N. L. Marana, J.-P. Flament, and B. Kirtman. “CRYSTAL23: a program for computational solid state physics and chemistry”. In: *Journal of Chemical Theory and Computation* 19.20 (2023), pp. 6891–6932. DOI: 10.1021/acs.jctc.2c00958.

[182] D. G. Anderson. “Iterative procedures for nonlinear integral equations”. In: *Journal of the ACM* 12.4 (1965), pp. 547–560. DOI: 10.1145/321296.321305.

[183] C. G. Broyden. “A class of methods for solving nonlinear simultaneous equations”. In: *Mathematics of Computation* 19.92 (1965), pp. 577–593. DOI: 10.1090/S0025-5718-1965-0198670-6.

[184] D. D. Johnson. “Modified Broyden’s method for accelerating convergence in self-consistent calculations”. In: *Physical Review B* 38.18 (1988), pp. 12807–12813. DOI: 10.1103/PhysRevB.38.12807.

[185] G. Kresse and J. Furthmüller. “Efficient iterative schemes for ab initio total-energy calculations using a plane-wave basis set”. In: *Physical Review B* 54.16 (1996), pp. 11169–11186. DOI: 10.1103/PhysRevB.54.11169.

[186] G. P. Kerker. “Efficient iteration scheme for self-consistent pseudopotential calculations”. In: *Physical Review B* 23.6 (1981), pp. 3082–3084. DOI: 10.1103/PhysRevB.23.3082.

[187] M. D. Towler, A. Zupan, and M. Causà. “Density functional theory in periodic systems using local Gaussian basis sets”. In: *Computer Physics Communications* 98.1–2 (1996), pp. 181–205. DOI: 10.1016/0010-4655(96)00078-1.

[188] R. Dovesi. “On the role of symmetry in the ab initio Hartree-Fock linear-combination-of-atomic-orbitals treatment of periodic systems”. In: *International Journal of Quantum Chemistry* 29 (1986), pp. 1755–1774. DOI: 10.1002/qua.560290608.

[189] R. S. Mulliken. “Electronic Population Analysis on LCAO-MO Molecular Wave Functions. I”. In: *Journal of Chemical Physics* 23.10 (1955), pp. 1833–1840. DOI: 10.1063/1.1740588.

[190] P.-O. Löwdin. “On the Non-Orthogonality Problem Connected with the Use of Atomic Wave Functions in the Theory of Molecules and Crystals”. In: *Journal of Chemical Physics* 18.3 (1950), pp. 365–375. DOI: 10.1063/1.1747632.

[191] F. L. Hirshfeld. "Bonded-atom fragments for describing molecular charge densities". In: *Theoretica Chimica Acta* 44.2 (1977), pp. 129–138. DOI: 10.1007/BF00549096.

[192] I. Mayer. "Charge, bond order and valence in the AB initio SCF theory". In: *Chemical Physics Letters* 97.3 (1983), pp. 270–274. DOI: 10.1016/0009-2614(83)80005-0.

[193] K. B. Wiberg. "Application of the Pople-Santry-Segal CNDO method to the cyclopropylcarbinyl and cyclobutyl cation and to bicyclobutane". In: *Tetrahedron* 24.3 (1968), pp. 1083–1096. DOI: 10.1016/0040-4020(68)88057-3.

[194] G. Knizia. "Intrinsic Atomic Orbitals: An Unbiased Bridge between Quantum Theory and Chemical Concepts". In: *Journal of Chemical Theory and Computation* 9.11 (2013), pp. 4834–4843. DOI: 10.1021/ct400687b.

[195] S. Huzinaga, J. Andzelm, M. Klobukowski, E. Radzio-Andzelm, Y. Sakai, and H. Tatewaki. *Gaussian Basis Sets for Molecular Calculations*. Elsevier, 1984.

[196] J. M. Foster and S. F. Boys. "Canonical Configurational Interaction Procedure". In: *Reviews of Modern Physics* 32.2 (1960), pp. 300–302. DOI: 10.1103/RevModPhys.32.300.

[197] J. Pipek and P. G. Mezey. "A fast intrinsic localization procedure applicable for ab initio and semiempirical linear combination of atomic orbital wave functions". In: *Journal of Chemical Physics* 90.9 (1989), pp. 4916–4926. DOI: 10.1063/1.456588.

[198] T. Hughbanks and R. Hoffmann. "Chains of trans-edge-sharing molybdenum octahedra: metal-metal bonding in extended systems". In: *Journal of the American Chemical Society* 105.11 (1983), pp. 3528–3537. DOI: 10.1021/ja00349a027.

[199] R. Dronskowski and P. E. Blöchl. "Crystal orbital Hamilton populations (COHP): energy-resolved visualization of chemical bonding in solids based on density-functional calculations". In: *The Journal of Physical Chemistry* 97.33 (1993), pp. 8617–8624. DOI: 10.1021/j100135a014.

[200] V. L. Deringer, A. L. Tchougréeff, and R. Dronskowski. "Crystal orbital Hamilton population (COHP) analysis as projected from plane-wave basis sets". In: *Journal of Physical Chemistry A* 115.21 (2011), pp. 5461–5466. DOI: 10.1021/jp202489s.

[201] A. D. Becke and K. E. Edgecombe. "A simple measure of electron localization in atomic and molecular systems". In: *Journal of Chemical Physics* 92.9 (1990), pp. 5397–5403. DOI: 10.1063/1.458517.

[202] E. R. Johnson, S. Keinan, P. Mori-Sánchez, J. Contreras-García, A. J. Cohen, and W. Yang. "Revealing Noncovalent Interactions". In: *Journal of the American Chemical Society* 132.18 (2010), pp. 6498–6506. DOI: 10.1021/ja100936w.

[203] R. F. W. Bader. "Atoms in molecules". In: *Accounts of Chemical Research* 18.1 (1985), pp. 9–15. DOI: 10.1021/ar00109a003.

[204] R. F. W. Bader. *Atoms in Molecules: A Quantum Theory*. Vol. 22. International Series of Monographs on Chemistry. Oxford: Oxford University Press, 1990.

[205] Y. Hinuma, G. Pizzi, Y. Kumagai, F. Oba, and I. Tanaka. "Band structure diagram paths based on crystallography". In: *Computational Materials Science* 128 (2017), pp. 140–184. DOI: 10.1016/j.commatsci.2016.10.015.
